# New Insights into the Phylogeny and Molecular Classification of Nicotinamide Mononucleotide Deamidases

**DOI:** 10.1371/journal.pone.0082705

**Published:** 2013-12-05

**Authors:** Guiomar Sánchez-Carrón, Ana Belén Martínez-Moñino, Agustín Sola-Carvajal, Hideto Takami, Francisco García-Carmona, Álvaro Sánchez-Ferrer

**Affiliations:** 1 Department of Biochemistry and Molecular Biology-A, Faculty of Biology, Regional Campus of International Excellence “Campus Mare Nostrum”, University of Murcia, Campus Espinardo, Murcia, Spain; 2 Murcia Biomedical Research Institute (IMIB), Murcia, Spain; 3 Microbial Genome Research Group, Institute of Biogeosciences, Japan Agency for Marine-Earth Science and Technology, Yokosuka, Kanagawa, Japan; University of Florida, United States of America

## Abstract

Nicotinamide mononucleotide (NMN) deamidase is one of the key enzymes of the bacterial pyridine nucleotide cycle (PNC). It catalyzes the conversion of NMN to nicotinic acid mononucleotide, which is later converted to NAD^+^ by entering the Preiss-Handler pathway. However, very few biochemical data are available regarding this enzyme. This paper represents the first complete molecular characterization of a novel NMN deamidase from the halotolerant and alkaliphilic bacterium *Oceanobacillus iheyensis* (OiPncC). The enzyme was active over a broad pH range, with an optimum at pH 7.4, whilst maintaining 90 % activity at pH 10.0. Surprisingly, the enzyme was quite stable at such basic pH, maintaining 61 % activity after 21 days. As regard temperature, it had an optimum at 65 °C but its stability was better below 50 °C. OiPncC was a Michaelian enzyme towards its only substrate NMN, with a *K*_*m*_ value of 0.18 mM and a *k_cat_/K*_*m*_ of 2.1 mM^-1^ s^-1^. To further our understanding of these enzymes, a complete phylogenetic and structural analysis was carried out taking into account the two Pfam domains usually associated with them (MocF and CinA). This analysis sheds light on the evolution of NMN deamidases, and enables the classification of NMN deamidases into 12 different subgroups, pointing to a novel domain architecture never before described. Using a Logo representation, conserved blocks were determined, providing new insights on the crucial residues involved in the binding and catalysis of both CinA and MocF domains. The analysis of these conserved blocks within new protein sequences could permit the more efficient data curation of incoming NMN deamidases.

## Introduction

**Figure 1:**
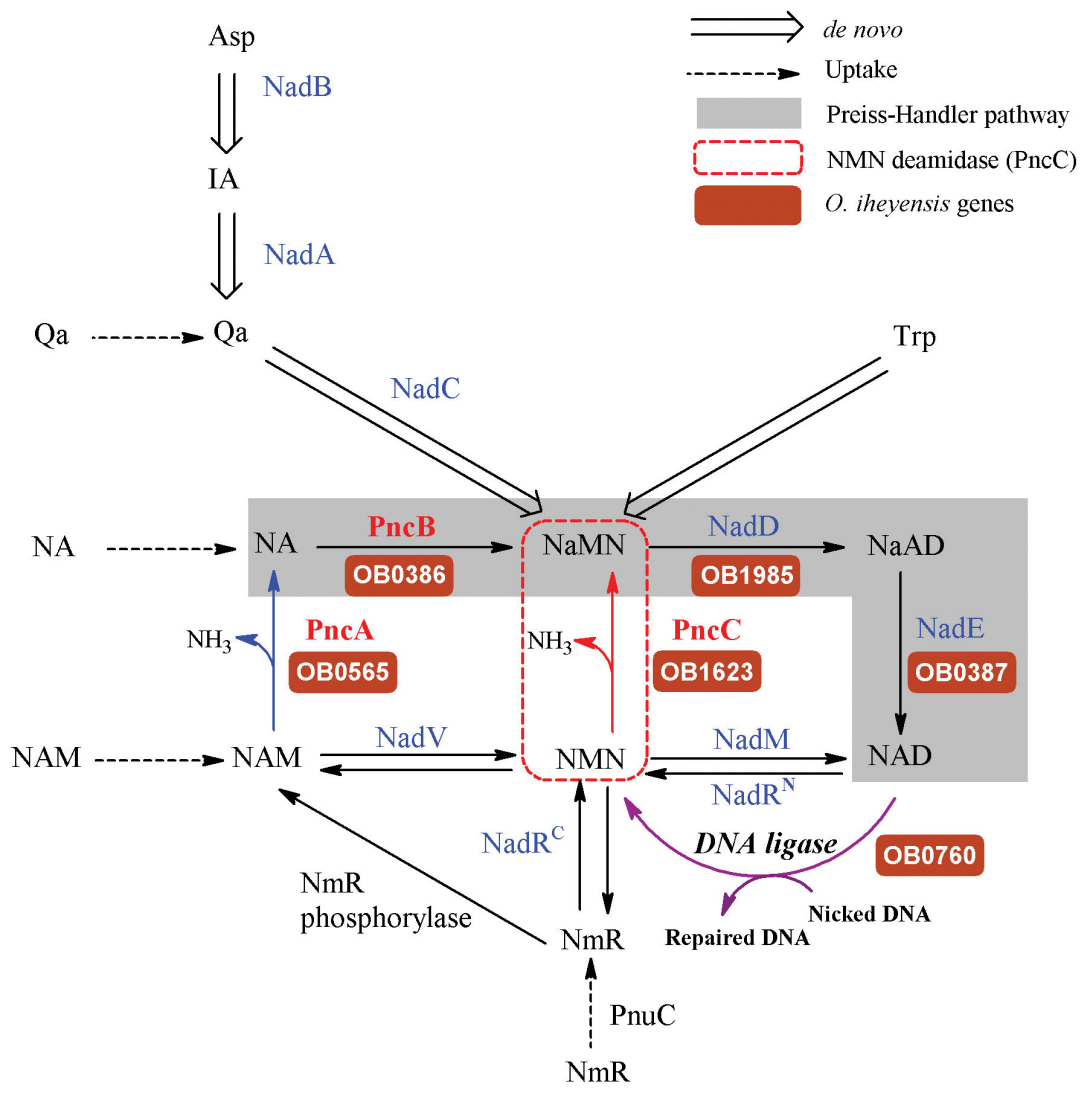
Pyridine nucleotide cycle and NAD^+^ biosynthetic routes. The routes known to be functional across diverse bacterial species are shown by solid lines. Preiss-Handler pathway is shadowed. Dashed and hollow arrows relate to uptake and *de* *novo* NaMN synthesis, respectively. Blue arrow corresponds to nicotinamidase activity (PncA), Red arrow corresponds to nicotinamide mononucleotide deamidase activity (PncC) and green arrow corresponds to NAD^+^-dependent DNA ligase. Enzymes are indicated as the acronym used to identify the corresponding gene locus: NadA, quinolinate synthetase; NadB, L-aspartate oxidase; NadC, quinolinate phosphoribosyl transferase; NadD, NaMN adenylyltransferase; NadE, NAD synthetase; NadM, NMN adenyltransferase; NadR^C^, NmR kinase; NadR^N^, NMN adenylyltransferase; NadV, Nm phosphoribosyltransferase; PncA, Nam deamidase; PncB, Na phosphoribosyltransferase; PncC, nicotinamide mononucleotide deamidase; PncU, nucleoside permease. The gene name of the enzymes existing in *O. iheyensis* is highlighted in brown. Abbreviations: NAD, nicotinamide adenine dinucleotide; IA, α-iminosuccinate; Qa, quinolonic acid; Asp, aspartate; Trp, tryptophan; NAM, nicotinamide; NA: nicotinic acid; NMN, nicotinic acid mononucleotide; NaAD, nicotinic acid adenine dinucleotide; NmR, nicotinamide riboside; DNA, deoxyribonucleic acid.

The enzyme nicotinamide mononucleotide (NMN) deamidase (EC 3.5.1.42) catalyzes the conversion of NMN to nicotinic acid mononucleotide (NaMN) and ammonia (Figure 1, red line). Such activity was first described in *Salmonella typhimurium* and *Escherichia coli* in the late 70s, as one of the reactions implicated in the Pyridine Nucleotide Cycle (PNC), which in turn, is associated with bacterial NAD^+^ salvage (Figure 1) [1-3]. In fact, its product NaMN is the connection point between *de novo* routes and the most commonly occurring NAD^+^ salvage pathway, the Preiss-Handler pathway (Figure 1, shadowed reactions). This NMN deamidase has also been related to the prevention of the inhibition of bacterial NAD^+^-dependent DNA ligase by its product (NMN) [4,5] (Figure 1, magenta line). The gene responsible for this activity, named *pncC* [6], belonged until recently to the group of “orphan enzymes”. However, in 2011 Galeazzi et al. isolated the native enzyme from the marine bacterium *Shewanella oneidensis* [7]. Surprisingly, its sequence corresponded to a protein annotated in protein databases as competence/damage-inducible protein A (CinA). CinA had been proposed to be involved in bacterial competence induction, given that it is a component of the *recA* operon [8,9]. However, this biological role is not totally established, since *Bacillus subtilis* CinA deletion mutants did not show a pronounced reduction in transformation efficiency and did not affect the localization of RecA [10]. This, together with its localization in the nucleoid [10] and the recent phenotype analysis of deletion mutants in *S. oneidensis*, suggest a possible role in maintaining low NMN levels and NAD^+^ recycling to ensure continued NAD^+^ supply to the ligase reaction [7]. 

**Figure 2:**
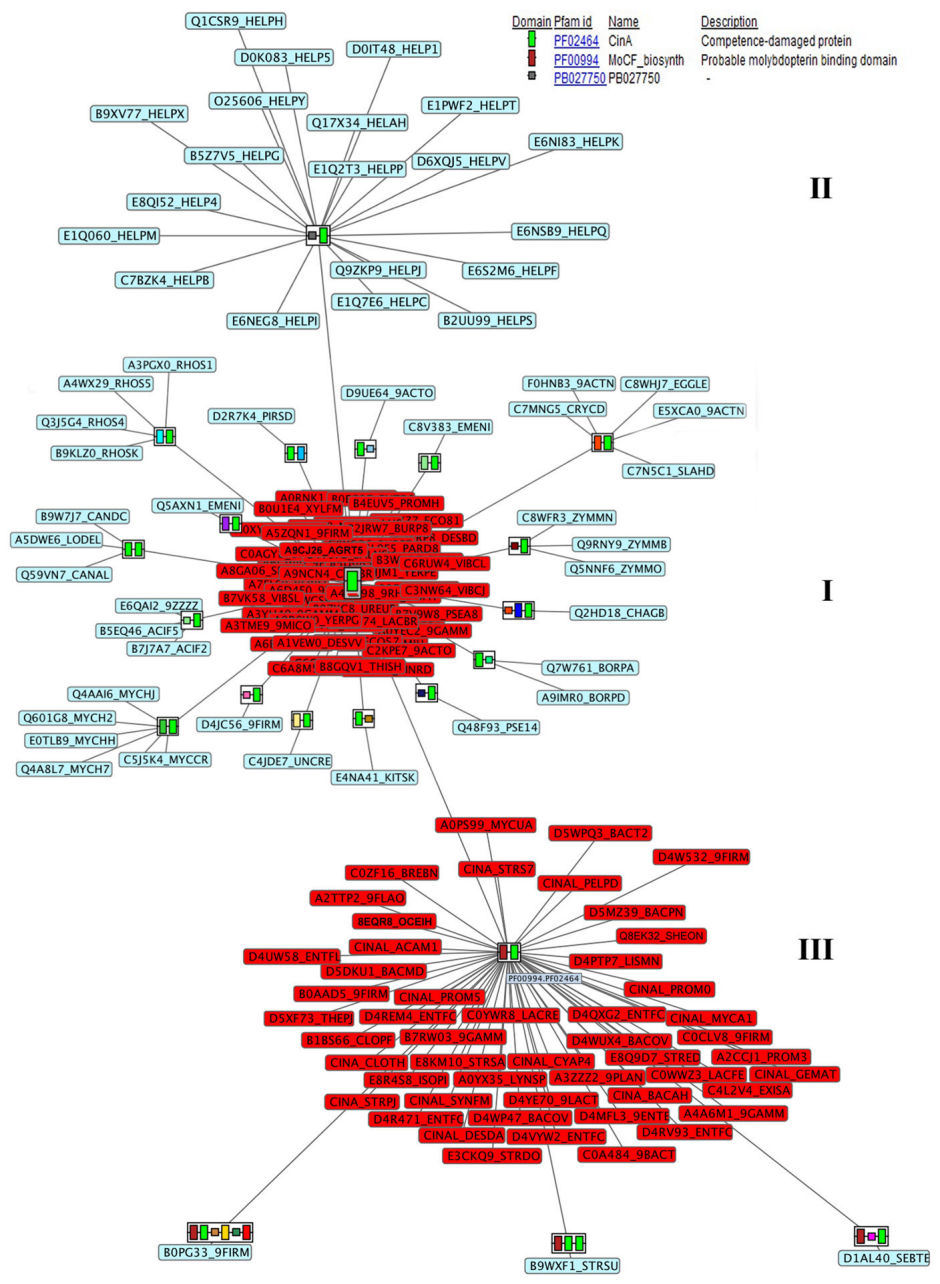
Plot of the different Pfam domain architectures found for PncC enzymes using ArchSchema. Green rectangles represent the CinA domain (Pfam ID: PF02464). Red rectangles represent the MocF domain (Pfam ID: PF00994). Other colored rectangles and squares represent other Pfam domains. Labels represent UniProt codes of enzymes belonging to each architecture.

From the structural point of view, only two structures, both designated as CinA, have been crystallized by Midwest Center for Structural Genomics (http://www.mcsg.anl.gov/) and deposited in the Protein Data Bank (http://www.rcsb.org/), one from *Agrobacterium tumefaciens* (PDB code: 2A9S, UniProt code: A9CJ26) and another from the archaea *Thermoplasma acidophilum* (PDB code: 3KBQ, UniProt code: Q9HKV6). However, structural and biochemical analysis showed these enzymes to be very different from each other: 2A9S contains the conserved residues responsible for NMN deamidase activity [7], whereas 3KBQ has been recently described as a new class of pyrophosphatase (eggNOG code: COG1058) [11]. When the structure of *A. tumefaciens* CinA (2A9S) was studied in detail using ArchSchema [12], a tool for interactive graphing of related Pfam domain architectures, three well-defined groups could be identified attending to domain composition (Figure 2). The first covers enzymes with the same architecture than 2A9S, showing only a CinA domain (Pfam code: PF02464), whereas the second shows enzymes containing one CinA domain bound to a N-terminal sequence tail of unknown function, generally associated with strains of *Helicobacter pylori* (Pfam code: PB027750) and a few in α- and γ-proteobacteria. Finally enzymes with a CinA domain fused to an N-terminal MocF domain (Pfam code: PF00994) formed a third group, which exhibits some degree of homology with the enzymes involved in the last steps of molybdenum cofactor biosynthesis [7,13]. In addition, this analysis showed that the CinA domain was occasionally fused with other domains and structures of known and unknown function. However, those proteins were not predicted to be Cin-A proteins from a search in UniProt database, and thus, they were not taken into consideration for further analysis.

Of these three main structural groups obtained by ArchSchema, two were also described by Galeazzi [7]: the NMN deamidases formed by one CinA domain, and the structural group of enzymes containing a MocF domain fused to a CinA domain. As described in [7], both single and two-domain NMN deamidases can be functional or nonfunctional, and more than one NMN deamidase of different architecture is occasionally found within the same microorganism. For example, *Escherichia coli* have three PncC homologues: YfaY, where a CinA domain is fused with a conserved MocF domain, as in *S. oneidensis*, and two paralogs, YgaD and YdeJ, comprising only the CinA domain [7]. This work also confirmed that NMN deamidase activity was retained in the CinA domain, which was re-named as PncC domain, since only *E. coli* YgaD showed activity towards NMN, whereas YfaY, which includes a MocF-domain, showed no activity [7]. The enzyme from *S. oneidensis*, which has a two-domain architecture, was also characterized and demonstrated to be functional [7]. This microorganism possesses genes involved in *de novo* biosynthesis of NAD^+^ from aspartic acid (via NadA, NadB and NadC, Figure 1), as well as *nadV* gene coding for the enzyme nicotinamide phosphoribosyltransferase (NadV), which initiates the amidated salvage/recycling of NAM [14]. However, *S. oneidensis* lacks the genes PncA and PncB (Figure 1), coding for the enzymes supporting conversion of NAM to NaMN via nicotinic acid (NA), which enters the Preiss-Handler pathway that leads to the salvage of NAD^+^. Unlike in the case of *S. oneidensis*, the genome of the chosen organism for this paper, *Oceanobacillus iheyensis*, does not contain the required enzymes for the *de novo* synthesis of NAD^+^, or for its salvage from NMN or NAM through the amidation pathway (via NadV, NadM, NadR^C^, Figure 1). Consequently, *O. iheyensis* relies exclusively on recycling routes based on the use of PncA and/or PncC, which both catalyze a similar deamidation reaction within the cell (Figure 1, blue and red lines). 

The aim of this paper was to biochemically characterize a new NMN deamidase from *O. iheyensis* (OiPncC), a deep-sea, extremely halotolerant and alkaliphilic bacteria isolated from 1050 m depth on the Iheya ridge [15], thus complementing our previous study on its corresponding nicotinamidase (OiPncA or OiNIC) [16]. Surprisingly, OiPncC was Michaelian enzyme with a high stability at basic pHs and with an optimum temperature of 65°C. The phylogenetic analysis showed that OiPncC has a different ancestral origin from previously characterized NMN deamidases, including that of *S. oneidensis* PncC. In addition, structural analysis of the sequences used in the above phylogenetic analysis showed the complete topology of the conserved blocks in both CinA and MocF domains. As a result of these two analyses, a new classification is proposed, dividing the NMN deamidases into two large groups and 12 subgroups. Such classification should permit a more efficient data curation, and provide a new nomenclature for the classification of incoming sequences.

## Materials and Methods

### Strains, plasmids, and chemicals

 Genomic DNA was isolated from *Oceanobacillus iheyensis* HTE831 strain deposited in JAMSTEC (Japan) [15]. The pET24b cloning vector was from Novagen (EMD Bioscience Inc. Madison, WI, USA). QIAquick PCR purification kit and QIAprep spin miniprep kit were from Qiagen (Valencia, CA, USA). KapaHiFi polymerase was from KapaBiosystems (Boston, MA, USA). NADPH was from Carbosynth (Berkshire, UK), nicotinamide mononucleotide was from Santa Cruz Biotechnology (Heidelberg, Germany). Other reagents were from Sigma-Aldrich (Madrid, Spain).

### Cloning of the OiPncC gene

 The cloning and transformation techniques used were essentially those previously described [17]. Genomic DNA from *Oceanobacillus iheyensis* HTE831 was used as the source of nicotinamide mononucleotide deamidase gen (UniProt code: Q8EQR8). The 417 bp gene was amplified by PCR using forward primer 5’-GCGGGCTAGCATGAAAAATTATCAAGCTGAAATAGTAG-3’ and reverse primer 5’-GCCGCTCGAGGCTTTTACTTTTTAAATATTGATATATTAGTTC-3’ (restriction enzymes cleavage sites are italicized). The resulting PCR product was purified and digested with NdeI and XhoI restriction enzymes, ligated to the digested pET24b, which carries a C-terminal His_6_-tag, and transformed into competent *E. coli* Rosetta2 (DE3) cells (Novagen). A selected clone harboring the correct sequence was denoted as pET24-OiPncC. 

### Expression and purification

 The above *E. coli* cells harbouring the recombinant plasmid pET24-OiPncC were grown for 2 hours at 37 °C in 100 mL of LB kan-Chlor before being transferred to a 2.5-L culture flask containing 1 L of Terrific Broth supplemented with antibiotics. This culture was allowed to grow for 3h at 37 °C, and then induced by adding 0.4 mM isopropyl-β-D-thiogalactoside (IPTG) for 14 hours at 20 °C with constant agitation. The culture was diafiltered through a 500 kDa membrane (GE Life Sciences, Uppsala, Sweden) and cleaned with 50 mM potassium phosphate buffer pH 8.0. After cell disruption in a homogenizer (MiniZetaII, Netzsch), the purification was performed in two steps, starting with tangential ultrafiltration with a 50-kDa cutoff membrane on a QuixStand system (GE Life Sciences). After centrifugation at 40,000*g*, the resulting supernatant was purified by Ni^2+^-chelating affinity chromatography (ÄKTA Prime Plus, GE Life Sciences) onto a HiPrep IMAC 16/10 FF 20 mL column (GE Life Sciences). The fractions containing the nicotinamide deamidase activity were pooled, desalted, concentrated and stored at -20 °C with 20% glycerol.

Gel filtration (Superdex 200, GE Life Sciences) was used to confirm the homogeneity and the molecular mass of the purified enzyme. In addition, mass spectrometry was performed using HPLC/ESI/ion trap system [17]. The protein concentration was determined using Bradford´s reagent (Bio-Rad) and BSA as standard.

### Enzyme assay

 Nicotinamide mononucleotide deamidation was determined both spectrophotometrically and by HPLC. In the first method, NMN deamidase activity was coupled with a NADPH-dependant glutamate dehydrogenase from *Bacillus halodurans* (BhGDH), to combine the ammonia released by NMN deamidase with α-ketoglutarate, rendering glutamate with the concomitant NADPH oxidation (Ɛ_360 nm_= 4320 M^-1^ cm^-1^). The standard reaction medium (200 μL) for the above assay at 37 °C, which was measured in a Synergy HT 96-well plate reader (Biotek), contained 300 μM NADPH, 9.7 μg BhGDH, 0.5 mM NMN, 10 mM α-ketoglutarate and 15 μg of purified OiPncC in 50 mM phosphate buffer pH 7.5. A control assay without NMN was also carried out to determine the presence of any other NADPH-consuming enzymes. One unit of activity is defined as the amount of enzyme consuming 1 μmol of NADPH in 1 min at pH 7.3 and 37 °C. Kinetic parameters were obtained after three repeated experiments. Activity was also measured spectrophotometrically when nicotinamide (NAM), pyrazimamide (PZA) and 5-methylnicotinamide were used as possible alternative substrates.

OiPncC activity was also measured by HPLC from the decrease of area of the nicotinamide mononucleotide (NMN) peak, using a C_18_ column (Phenomenex Gemini C_18_, 4.6 x 250 mm) and mobile phase (20 mM ammonium acetate pH 6.9) running at 1 mL/min. Under these conditions, the retention time (R_T_) for NMN and nicotinic acid mononucleotide (NaMN) were 3.2 and 2.9 min, respectively. One unit of activity was defined as the amount of enzyme required to cleave 1 μmol of NMN releasing 1 μmol of NaMN in 1 min. The standard reaction medium (1 mL) for the HPLC reaction at 37 °C was 0.5 mM NMN and 75 μg purified OiPncC in 50 mM phosphate buffer pH 7.5. Reactions were stopped by addition of TFA to a final pH of 3.0. Kinetic parameters were obtained after three repeated experiments. Activity was also measured by HPLC for NAM, PZA, 5-methylnicotinamide, methylnicotinate and ethylnicotinate. 

### Stability assays

OiPncC pH-stability was spectrophotometrically assayed measuring the residual activity of OiPncC after incubation at different pHs at 37 °C. Heat-stability assay was carried out by incubating the enzyme at pH 7.3 from 4 to 60 °C using a water bath. Aliquots of 50 µL were taken at different times, cooled on ice and 10 µL of these aliquots were then spectrophotometrically assayed in the standard reaction media.

### Structural, topological and phylogenetic analysis

 BLAST searches [18] and structural alignment with Consurf [19] were used to identify homologues of PncC. The sequences were aligned using ClustalW [20] and ESPript [21]. Protein sequences were 3D modeled with Geno3D [22] and ModWeb [23]. Molecular visualizations were performed with PyMOL (http://pymol.org) [24] and Chimera [25]. Domain composition analysis of NMN deamidases was carried out using Pfam database [26] and ArchSchema [12]. Distribution analysis of PncCs was carried out using the HMMER web server [27] and UniProt database [28]. A sequence significance E-value threshold of 1e^-45^ (Hit: 3e^-45^) was chosen, in order to eliminate false non-homologous results. Tree-building method used was Neighbor Joining (NJ) as implemented in Archaeopteryx [29]. The Bootstrap values for NJ trees were obtained after 1000 generations [30]. Display and manipulation of phylogenetic trees was made by Interactive Tree of Life (iTOL) [31]. 16S rRNA sequences were obtained from Silva database [32]. Docking was performed with Molegro Virtual Docker (CLC Bio, Aarhus, Denmark) using MolDock option and the default settings [33]. Conserved blocks were detected using WebLogo3 [34].

## Results

### Amino acid sequence comparison

*Oceanobacillus iheyensis* HTE831 NMN deamidase-encoding protein (OiPncC) was found in UniProt database (Q8EQR8) as a 417 amino acids putative competence-damage inducible protein (CinA protein). When it was used as template for a BLAST search [18], the protein showed a moderate identity with other putative competence-damage inducible proteins, such as those from *Geobacillus kaustophilus* (UniProt entry: Q5L0F7), *Bacillus subtilis* (UniProt entry: E8VBF4), and *Listeria monocitogenes* (UniProt entry: D2P222), with 48 %, 47 %, and 46 % amino acid sequence identity, respectively. OiPncC also showed 34 % identity with the functionally characterized NMN deamidase from *Shewanella oneidensis* (UniProt entry: Q8EK32) [7].

Four types of NMN deamidases have been described [7] according to functionality and domain-composition. In most cases, such as in the case of the NMN deamidase from *Agrobacterium tumefaciens* (UniProt code: A9CJ26; PBD code: 2A9S) or *Escherichia coli* YgaD (UniProt code: P0A6G3), NMN deamidases are formed by just one domain containing the deamidase activity, the so-called PncC domain or CinA domain (Figure S1). In other cases, PncC domain is fused in its N-terminal with a MocF domain, which exhibits some degree of homology with enzymes involved in the last step of molybdenum cofactor (Moco) biosynthesis (gephyrin, MogA, plant Cnx1 and MoeA [13]. This is the case of the described *S. oneidensis* PncC (UniProt code: Q8EK32) [7] and also the enzyme from *O. iheyensis* (UniProt code: Q8EQR8) (Figure S1). In addition, OiPncC seems to have all the described amino acids forming part of the active site [7]: E281, S282, T284, G286, S299, Y309, K314, S/T356, G357 and R394 (Figure S1, filled triangles, OiPncC numbering). However, non-functional versions of the one- and two- domain enzymes, such as *E. coli* YdeJ (UniProt code: P31131) and *E. coli* YfaY (UniProt code: P77808) show mutations in one or more of the above amino acids (Figure S1).

### Cloning, overexpression and purification of OiPncC

The annotated *CinA* gene from *O. iheyensis* HTE831 (OiPncC) was amplified and inserted downstream of the IPTG inducible promoter of pET24b vector, which includes a 6×His-tag in C-terminal. Recombinant vector pET24b-OiPncC was transformed and induced in *E. coli* Rosetta2 (DE3). From this soluble cell extract obtained at 20 °C, recombinant protein was purified in two single steps, as described in Materials and Methods. The purified OiPncC appeared on SDS-PAGE as a single band (Figure S2, lane 3), which corresponds to a molecular weight of about 47 kDa. This datum together with those obtained by HPLC/MS/ESI (47.32 kDa) and gel filtration (96.1 kDa) (data not shown), confirmed the dimeric nature of OiPncC.

### Biochemical characterization of recombinant OiPncC

The enzyme was active towards NMN rendering NaMN, as measured by HPLC (Figure S3A). When NMN (retention time 3.2 min) was incubated in the presence of purified OiPncC, a new peak appeared with retention time at 2.9 min, which corresponded to a commercial standard NaMN (Figure S3A). This activity was also demonstrated using a spectrophotometric enzyme-coupled method, in which the NH_3_ produced by OiPncC was coupled with the reductive amination of α-ketoglutarate to produce glutamate in the presence of NADPH, catalyzed by *Bacillus halodurans* NADPH-dependent glutamate dehydrogenase (BhGDH), leading to the concomitant decrease in absorbance at 360 nm (Figure S3B). No activity was found in absence of OiPncC, NADPH or NMN. This method, used before to measure sirtuins and/or nicotinamidases [35], has never been applied before for the measurement of NMN deamidase activity. The result obtained with the spectrophotometric method was the same as with HPLC (see below), but with the clear advantage, for the first time, of a cost-effective determination in a microtiter plate scale (200 µL).

**Figure 3:**
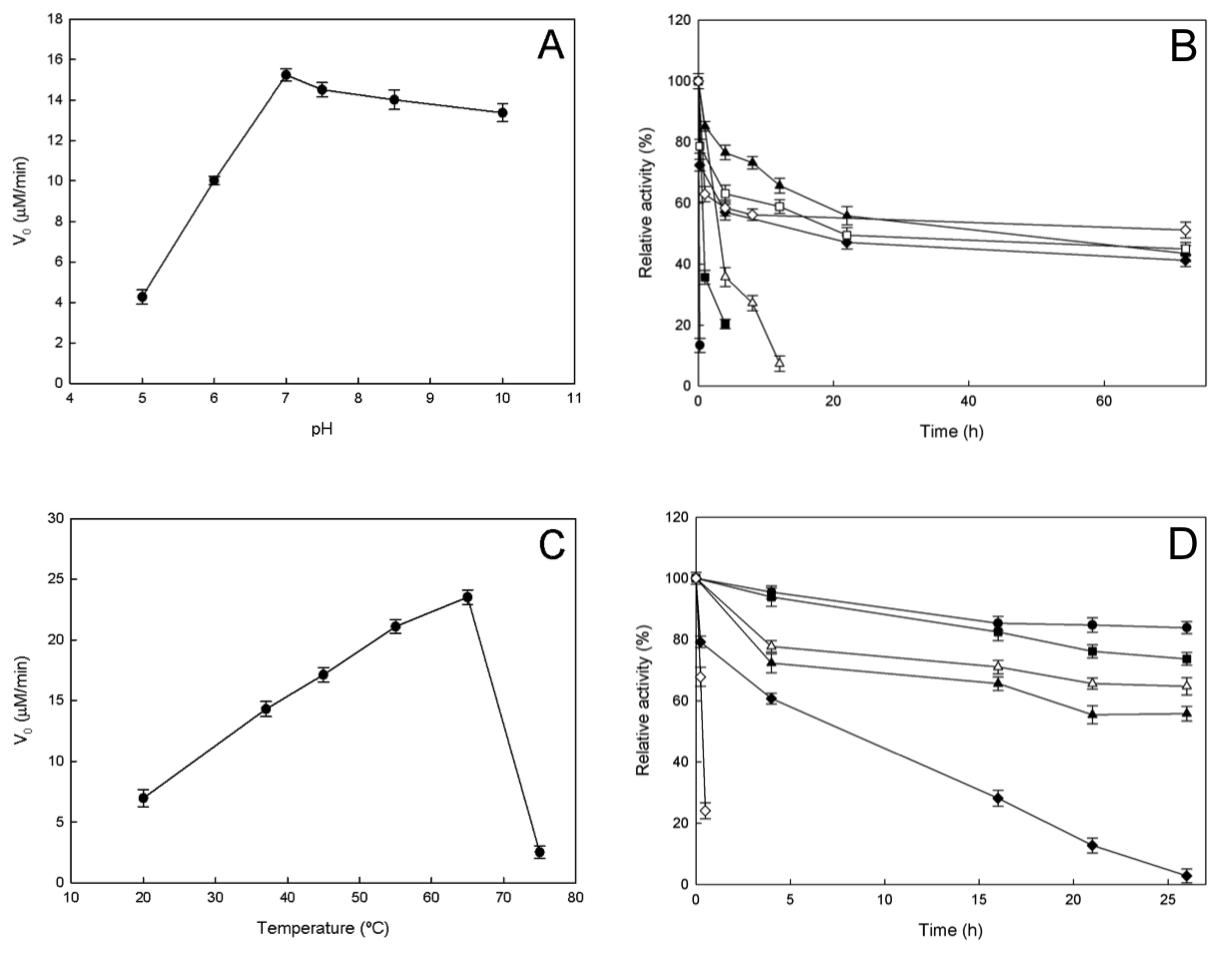
Effect of pH and temperature on OiPncC. **A**) Effect of pH on OiPncC activity measured by the HPLC Assay. The buffers used were 50 mM sodium acetate (pH 5.0), 50 mM potassium phosphate buffer (pH 6.0-7.4) and glycine-NaOH (pH 8.5-10.0). **B**) pH-stability. Aliquots of enzyme incubated at different pHs were removed and relative activity was measured using the enzyme-coupled assay at different times. The buffers used (50mM) were sodium acetate pH 5.0 (●), potassium phosphate pH 6.5 (■), pH 7.0 (Δ), pH 8.0 (▲), Tris-HCl pH 9.0 (♦), glycine pH 10 (□) and pH 10.5 (◊). **C**) Effect of temperature on OiPncC activity measured by the HPLC assay. **D**) Thermostability assay. Aliquots of enzyme incubated at different temperatures [4 °C (●), 20 °C (■), 37 °C (Δ), 45 °C (▲), 50 °C (♦) and 60 °C (◊)] were removed at different times and relative activity was measured using the enzyme-coupled assay. Standard assay conditions were used in all cases.

The activity of OiPncC was pH-dependent, displaying an optimum pH around 7.0-7.5 (Figure 3A). This analysis was performed by HPLC in order to avoid any possible effect arising from the pH sensitivity of the coupled assay. The enzyme activity decreased drastically at acidic pHs, however it maintained 88 % activity at pH 10.0. This data contrast with those described for *S. oneidensis* PncC, which showed a broad optimum pH (pH 5.0-9.0) and 72 % residual activity at pH 10.0 [7]. In general, the described PncCs have basic optimum pH, such as *E. coli* PncC (pH 9.0) [3] and *Salmonella typhimurium* PncC (pH 8.5-8.7) [1,36], except for one acidic example found in *Propionobacterium shermanii* (pH 5.6) [37].

A detailed study of pH-stability of NMN-deamidases has not been previously reported. When OiPncC was incubated at 37 °C at different pH values and the residual activity measured using the standard spectrophotometric method, OiPncC appeared to be very stable at basic pHs from pH 8.0 to 10.0, maintaining around 50 % of its activity at pHs above 7.0 for 72 hours (Figure 3B). Activity was also detected after 21 days of incubation at pH 10.0 (61 %) and pH 10.5 (22 %) (data not shown). On the other hand, the enzyme was quickly inactivated after a few hours at acidic pHs, ranging from 5.0 to 6.0 (Figure 3B).

When the effect of temperature on OiPncC was studied (Figure 3C), the activity increased with temperature up to 65 °C, above which activity fell dramatically. This optimum temperature was similar to the only one datum available in the bibliography for *Azotobacter vinelandii* (64 °C) [38]. This optimum temperature contrasted with its low stability at this temperature. At 60 °C the enzyme lost activity in few minutes (Figure 3D, open diamonds), whereas at 50 °C, the enzyme displayed a half-life of 8 hours (Figure 3D, closed diamonds). The stability increased below 50 °C showing half-life greater than 24 hours (Figure 3D). 

### Substrate specificity and kinetic constants of OiPncC

The substrate specificity of OiPncC was assayed with different substrates: NMN, nicotinamide, 5-methylnicotinamide, ethylnicotinate, methylnicotinate and pyrazinamide. The enzyme was highly specific for NMN, since no detectable deamidase activity was observed using the alternative substrates. Kinetic parameters for OiPncC were determined both by HPLC and by the enzyme-coupled spectrophotometric assay. The *K*_*m*_ calculated for NMN by HPLC was 0.18 ± 0.02 mM (Figure S4A) with a *k*_cat_ of 0.38 ± 0.01 s^-1^ and a *k*_cat_/*K*_*m*_ of 2.1 mM^-1^ s^-1^. These values are very close to those spectrophotometrically determined, which revealed a *K*_*m*_ of 0.20 ± 0.01 mM (Figure S4B) with a slightly lower *k*_cat_ of 0.4 ± 0.02 s^-1^ and a *k*_cat_/ *K*_*m*_ of 2 mM^-1^ s^-1^. In both cases, the plot of the initial velocity of the enzyme-catalyzed reaction versus NMN concentration showed a Michaelis-Menten curve (Figure S4). This contrast with the available data for *S. typhimurium* and S. oneidensis PncCs, which have been described as allosteric enzymes, since they showed sigmoidal profile as NMN concentration increased [1,7,36]. The Hill´s coefficient was determined to be 2.6 for *S. oneidensis*, indicating a strong positive cooperativity, with a S_0.5_ value of 6 μM and a *k*_cat_ value of 3.3 s^-1^ [7]. In contrast, *E. coli* PncC (*K*_*m*_ 140 μM), *Propionibacterium shermanii* PncC (*K*_*m*_ 70 μM) and *Azotobacter vinelandii* PncC (*K*_*m*_ 1 mM) were described as non-allosteric in the bibliography [3,37,38]. 

### NMN deamidases distribution and phylogenetic analysis

**Figure 4:**
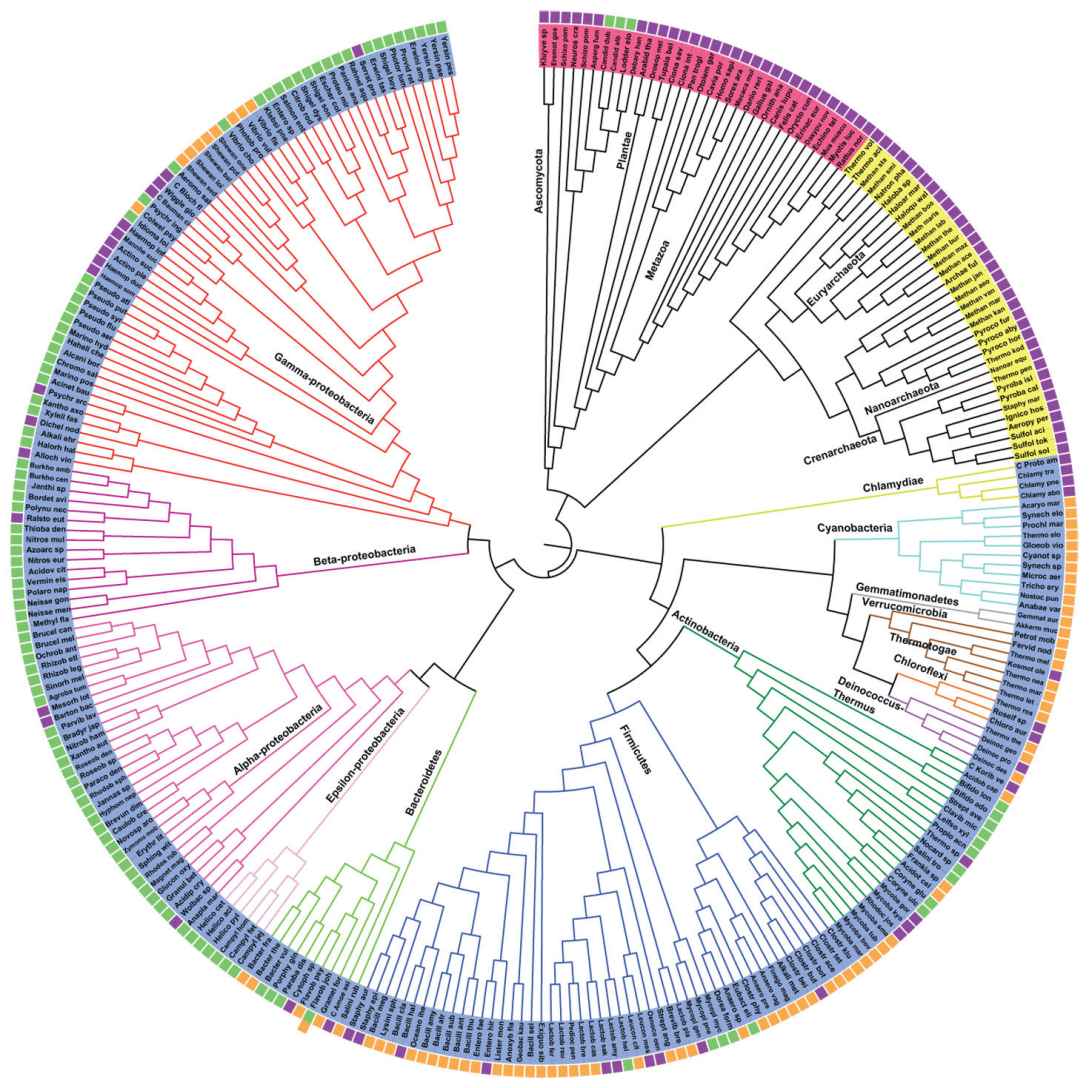
Distribution analysis of NMN deamidases. The figure shows a representative Tree of Life based on 16S rRNA. Species of Kingdom Eukarya are coloured red, those of Kingdom Archaea are coloured yellow and those of Kingdom Bacteria are coloured blue. The box next to each species represent the absence of OiPncC (purple), the presence of the functional one-domain enzyme (green) or the presence of a functional two-domain enzyme (orange) in the organism. The image was generated with iTOL [31]. .

An exhaustive search for the presence/absence of two-domain (MocF-CinA) and single domain (CinA) PncCs across biology was carried out. To visualize the results, a tree of life was generated using 16S rRNA (Figure 4), in which every organism shows a colour code corresponding to the absence of PncC (purple box), the presence of single functional domain (green box) or a two-domain functional PncC (orange box) in its own genome (Figure 4). In a few cases, more than one type of PncCs were found in one organism (i.e., *E. coli, Salmonella typhimurium, Flavobacterium johnsoniae*, *Photobacterium profundum, Vibrio cholerae*, etc), although not all of these enzymes are functional (as shown in Figure 4). For example, *Escherichia coli* or *Salmonella* contain one functional PncC, but also non-functional single or two-domain enzymes (active *E. coli* YgaD *vs* YdeJ or YfaY, respectively) [7].

 As expected, a vast majority of PncCs are found in the Bacterial Kingdom (Figure 4, blue), with just a few curious exceptions of sequences discovered in Archaea (Figure 4, yellow) and in the Eukaryotic Kingdom (Figure 4, red), mostly in Fungi. Among Bacteria, it seems clear that most two-domain NMN deamidase sequences (Figure 4, orange boxes) are found among Gram positive bacteria of the Phyla Firmicutes (45 %) and Actinobacteria (9 %). The main representatives among Gram negative bacteria were Cyanobacteria (14 %), Proteobacteria (11.5 %) and Bacteroidetes (7.5 %). In contrast, when studying single domain PncCs (Figure 4, green boxes), the tree revealed that approximately 79 % of the sequences found belong to Gram negative bacteria of Phylum Proteobacteria, 9 % to Actinobacteria, 4.5 % to Firmicutes and 4 % to Bacteroidetes. According to HMMER web server [27], around 1600 sequences in the database show the single CinA domain architecture, whilst around 1000 sequences display the MocF-CinA domain architecture. This contrasted with the results obtained from the Pfam database, which pointed to 2911 sequences corresponding to 1 domain NMN deamidases and 1484 corresponding to two-domain enzymes. Therefore, the HMMER web server [27] seemed to be more selective and was chosen for further analysis, in order to eliminate false non-homologous results.

**Figure 5:**
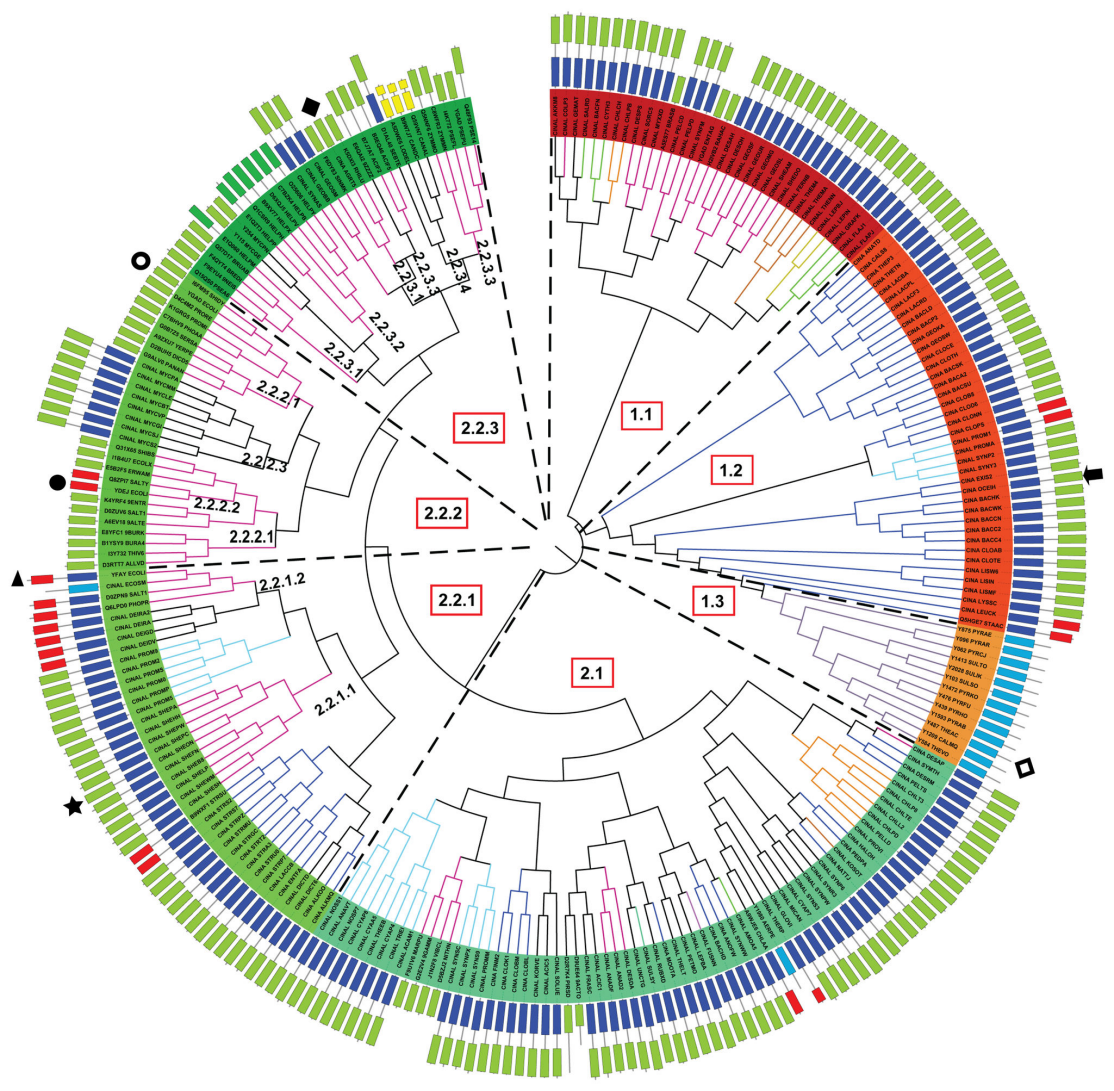
Phylogenetic analysis of NMN deamidases. The structures behind each organism name represent domain composition of the enzyme: MocF domain (dark blue); functional CinA domain (green); non-functional CinA domain (red); Eukaryotic PncCs (yellow). Special domains with only MocF domain are shown in light blue. C-terminal section of the protein is the outer part of the domain representation. Branch colours represent the same phylum as in Figure 4. The tree was built using Archaeopteryx [29], bootstrap values were obtained after 1000 generations. The arrow and the star indicate the position of OiPncC and *S. oneidensis* PncC, respectively. Other symbols are: *E. coli* YFAY (▲), *E. coli* YDEJ (●), *E. coli* YGAD (○), *T. acidophilum* CinA (◊) and *A. tumefaciens* CinA (♦).

 In order to shed light on the classification and phylogeny of PncCs, BLAST searches [18] and structural alignments with Consurf [19] using the only two crystals in PDB (3KBQ and 2A9S) were carried out. A representative number of PncCs sequences (255 sequences) were cured by hand, and then, checked with the domains shown in Pfam database [26] and ArchSchema [12] to build a new phylogenetic tree of NMN deamidases (Figure 5, Table S1). Coloured rectangles next to enzyme names represent the domain composition of each enzyme in N-ter to C-ter direction (Figure 5). The branches are also coloured according to the Phylum of each species shown in Figure 4. 

Two well-defined branches were noticeable, suggesting two different origins for bacterial PncCs. The first branch included enzymes with an active CinA domain (Figure 5, light green rectangles) fused to a MocF domain (Figure 5, dark blue rectangles), with a few single domain (Figure 5) and four inactive two-domain enzymes (Figure 5, red rectangles). This first origin could be divided into three different lineages. Lineage 1.1 was heterogeneous regarding Phylum composition and included enzymes from Proteobacteria, Bacteroidetes, Spirochaetes and Themotogae, whereas lineage 1.2 comprised bacteria belonging to Phylum Firmicutes, forming a conserved clade, where OiPncC was found (Figure 5, arrow). Curiously, branching apart from Lineage 1.2, a group of enzymes belonging to Archaea was found, forming Lineage 1.3 (Figure 5, light blue rectangles). All these archaea sequences are annotated in the protein databases as Cin-A proteins, including the crystallized one from *Thermoplasma acidophilum* (PDB code: 3KBQ, UniProt code: Q9HKV6; Figure 5, square), which is in fact annotated in the PDB as a “CinA protein with unknown function”. However, a close study of its sequence and domain composition revealed that these proteins (including 3KBQ) only showed the MocF domain (Figure 5) and lack the functional CinA domain. In nature, the MocF domain is found fused to a great number of other domains in different proteins in the Pfam database. Recently, 3KBQ has been described as a member of a new pyrophosphatase family and designated by its eggNOG (evolutionary genealogy of genes: Non supervised Orthologous Groups; http://eggnog.embl.de) code as *Thermoplasma acidophilum* COG1058 [11]. However, these archaea sequences found in Figure 5 seem to be a particular case, since the high similarity between the MocF domain found in archaea and the MocF domain of PncC proteins from Firmicutes suggests that archaea might have obtained the two-domain PncC gene from Firmicutes, while a subsequent deletion could have removed the CinA domain. Alternatively, the MocF domain could be transferred to archaea by horizontal gene transfer from Firmicutes.

The second origin of PncCs pointed to greater diversity in Phylum and domain-composition types. Of note was the fact that most single-domain enzymes appeared in this branch, suggesting a possible evolution from two-domain functional PncCs in this lineage. In addition, all the biochemically characterized NMN deamidases until now belonged to this second branch (Figure 5, symbols except diamonds). Overall, two main lineages could be described (Lineage 2.1 and 2.2). The greatest part of lineage 2.1 was formed by two-domain functional enzymes (Figure 5) of Phyla Cyanobacteria, Firmicutes, Aquificae, Fusobacteria and Chlorobi. Lineage 2.2 included three different domain architectures. Lineage 2.2.1 was also formed by functional two-domain enzymes (Figure 5), such as the characterized enzyme from *Shewanella oneidensis* (Lineage 2.2.1.1) (Figure 5, star). Curiously, a branch of inactive two-domain enzymes also belongs to this clade (Figure 5, lineage 2.2.1.2) represented by the characterized YfaY enzyme from *E. coli* (Figure 5, triangle) [7]. Lineage 2.2.2 showed enzymes with different domain architectures: active single-domain (Figure 5, lineage 2.2.2.1), inactive single-domain (Figure 5, Lineage 2.2.2.2) and active two-domain proteins belonging to *Mycobacterium* species (Lineage 2.2.2.3). Interestingly, the characterized *E. coli* proteins YgaD (active, Figure 5, open circle) and YdeJ (inactive, Figure 5, closed circle) were included in lineage 2.2.2. Finally, lineage 2.2.3 is essentially formed by active one-domain enzymes (Figure 5, Lineage 2.2.3.1), such as crystallized *Agrobacterium tumefaciens* CinA (CINA_AGRT5; PDB: 2A9S) (Figure 5, diamond). A close analysis of this lineage revealed, for the first time, the existence of PncCs with a domain architecture different from the four above described architectures (functional one- and two-domains enzymes and nonfunctional one- and two-domains enzymes). This new architecture consisted of a CinA with a short or a long N-terminal extension (Lineage 2.2.3.2 or Lineage 2.2.3.3, respectively). This extension resulted in enzymes with approximately 220 amino acid residues, which was larger than single-domain enzymes (around 160-170 residues), but shorter than the two-domain enzymes (around 420 residues). Lineage 2.2.3.2 was formed by enzymes from different strains of *Helicobacter pylori* and Lineage 2.2.3.3 comprised enzymes from Gram negative Proteobacteria. Surprisingly, another new PncC architecture appeared almost at the end of the tree (Lineage 2.2.3.4) formed by three sequences belonging to Phylum Fungi (Figure 5, yellow rectangles) that are annotated in the database as probable CinA proteins. The Pfam database and ArchSchema indicated the presence of two separated PncC domains in these proteins. However, an in-depth study revealed that those enzymes only have one PncC domain, which has been split in two parts by a spacing sequence, probably due to an insertion of a mobile genetic element.

### OiPncC structural analysis of conserved blocks

**Figure 6:**
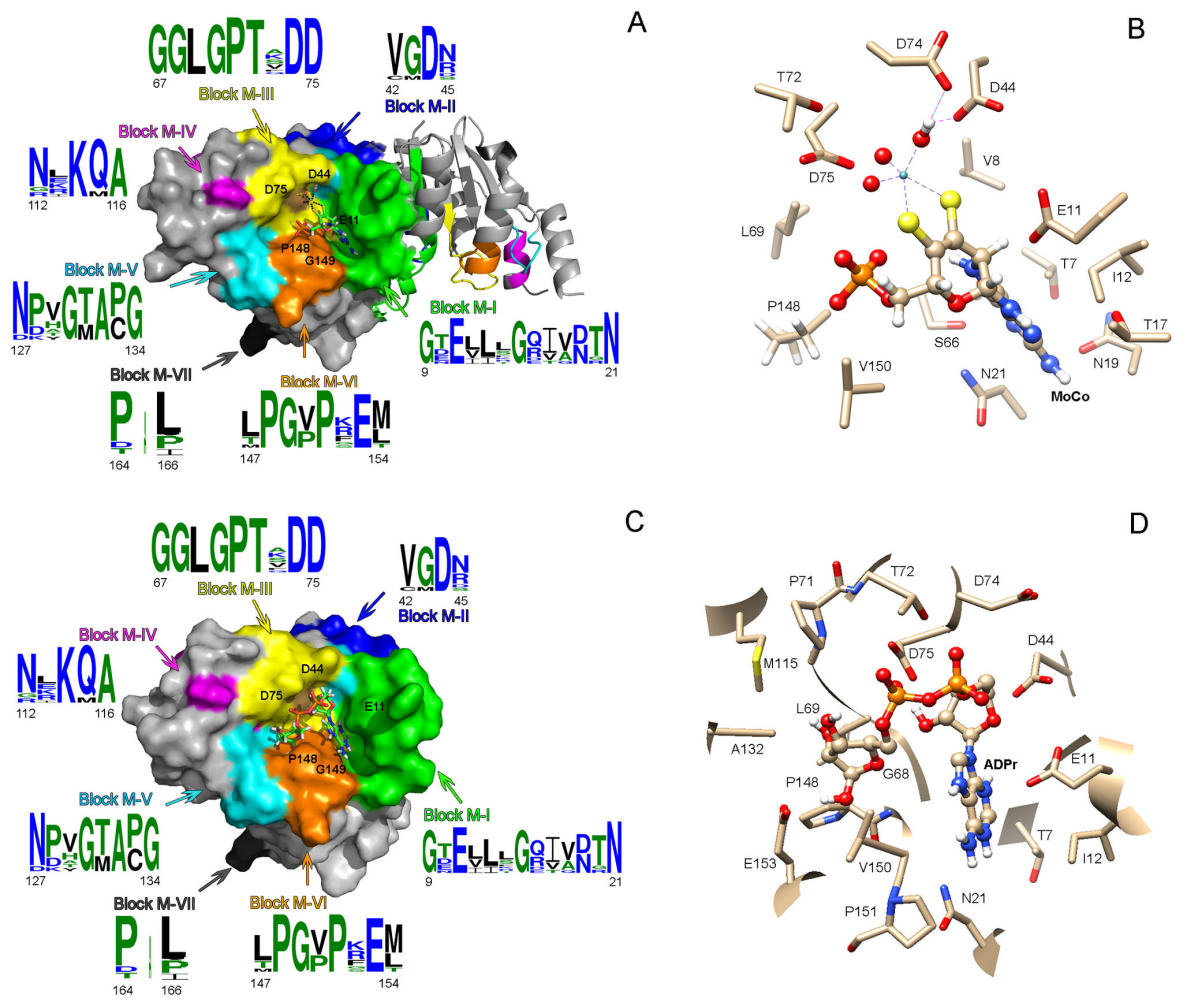
Structural analysis of MocF domain. A) Surface (subunit A) and ribbon (subunit B) representation of the dimeric *Thermoplasma acidophilum* CinA protein (PDB code: 3KBQ); conserved blocks forming the binding site are colored, and its consensus sequence shown as generated by WebLogo [34]. A Molybdenum cofactor molecule (Moco) in the proposed binding site is shown in ball and stick representation. B) Detailed view of the amino acids involved in the interaction between 3KBQ and Moco rendered by Chimera [25]. C) An ADPr molecule in the proposed binding site (subunit A) is shown in ball and stick representation. D) Detailed view of the amino acids involved in the interaction between 3KBQ and ADPr.

**Figure 7:**
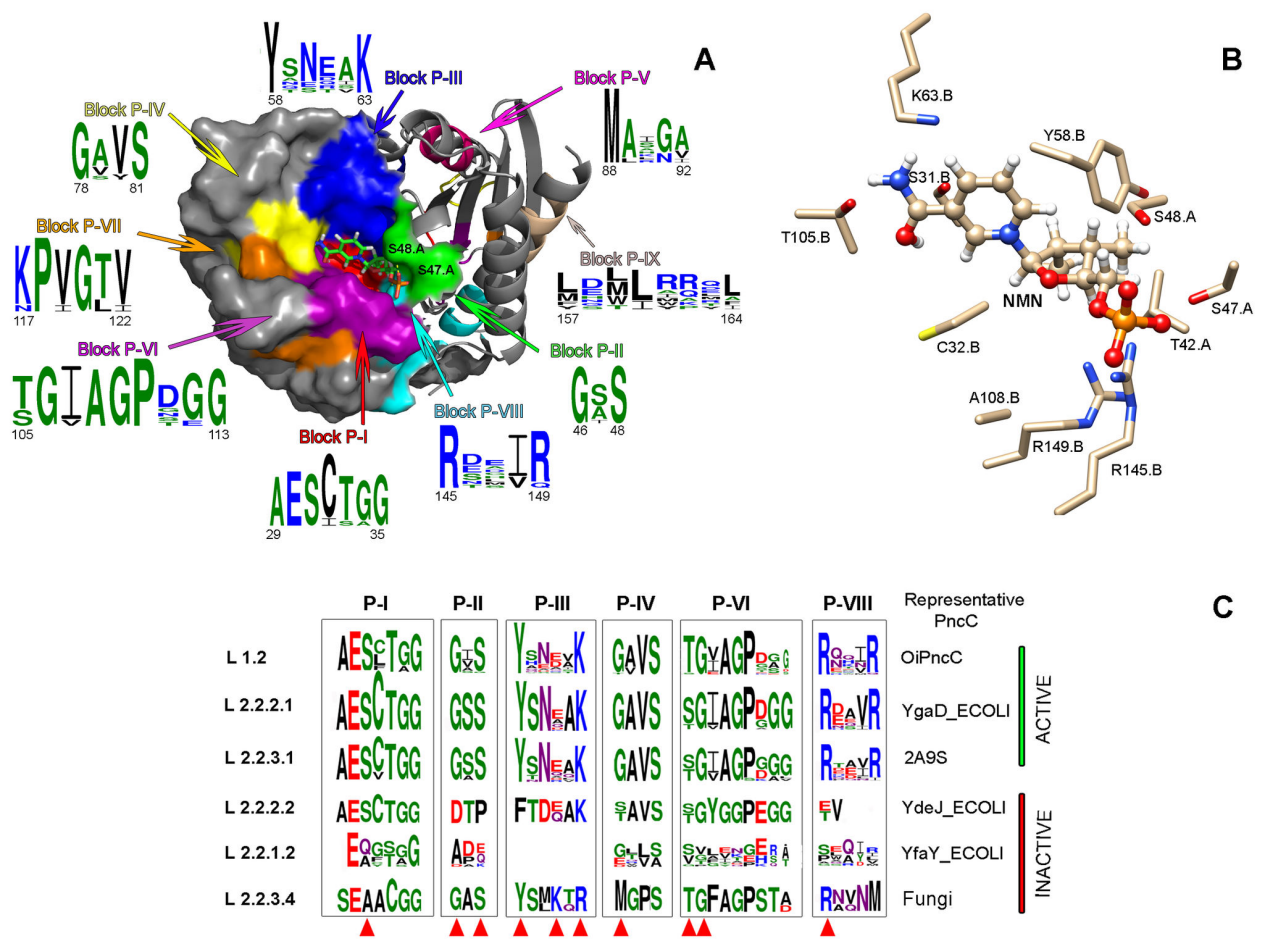
Structural analysis of CinA domain. A) Surface and ribbon representation of the *Agrobacterium tumefaciens* CinA dimer (PDB code: 2A9S); conserved blocks forming the binding site are colored and its consensus sequence are shown. A NMN molecule in the proposed binding site is shown in ball and stick representation. B) Detailed view of the amino acids involved in the interaction between 2A9S and NMN. C) Logo representations of the multiple alignments of the conserved blocks of CinA domain in representative active and inactive PncCs. The key residues involved in the catalytic process are marked with a red triangle.

 In order to fully understand this new classification of PncCs, a detailed study of conserved blocks was carried out using WebLOGO [34] and ESPript [21] representations of the different PncC lineages (Figures S5-S8), and subsequent mapping in the two known crystallized structures: 3KBQ for MocF domain and 2A9S for PncC/CinA domain (Figures 6 and 7).

Seven conserved blocks were found in the MocF domain (blocks M-I to M-VII, Figure S5, Figures S7-S8). Block M-I started with the sequence **G** T/D **E**xxx**G**xxx D/N Tx**N** and ranged from the end of β1 to the first three residues of α2 (3KBQ secondary structure; Figure S7, Lineage 1.3). This block had a highly conserved signature in all lineages. As shown in the Figure 6A (green colored), this block formed one of the walls of the pocket where the molibdopterin ring of Molybdenum cofactor (Moco) apparently binds. This block was also involved in the dimerization of the two subunits along with block M-II (Figure 6A blue), which was found between β2 and α3 (Figure S7, Lineage 1.3), and has the consensus sequence VG**D**NxxxH. Curiously, this D44 (*T. acidophilum* 3KBQ numbering) was the only strictly conserved residue across all lineages, and was also the only residue of the block that formed part of the binding pocket in the same position as catalytic D49, D61 and D228 in MogA, human gephyrin and MoeA, respectively [39-41]. The third block on the MocF domain (Figure S5, Block M-III) was well defined and strictly conserved across all lineages, closely resembling the conserved sequence of MogA and gephyrin, known as GGTG motif (G_86_GTG_89_ in Gephyrin numbering) [39,40]. In fact, this block with sequence **GGLGPT**x**DD**xT was located in the loop between β3 and α4 (Figure S7, lineage 1.3) and formed a large fraction of the binding pocket (Figure 6A, yellow). In particular, D75 (the last **D** of this block) together with D44 of block M-II could have a similar function in catalysis/binding of substrate as in MogA, MoeA and gephyrin. In addition, they are located close to the crystallized sulphate molecule in 3KBQ, which resembles the Molybdenum insertion position of Moco (Figure 6B) [40]. Unlike block M-III, block M-IV (Figure 6A, magenta; Figure S5) was less conserved between the different lineages, presenting a consensus sequence of Nx K/R QA. However, lineage 1.3 showed a clear fingerprint sequence for this block (**R**x**KMA**xx**P**), which was greatly conserved across all the archaea forming this lineage (Figure S7, lineage 1.3). The K114 and M115 of this block also faced the substrate binding surface. The sequence **N**xx**G**x**A**P**G** made up the conserved block M-V, which laid between β6 and the beginning of β7 (Figure 6A, cyan; Figure S7, lineage 1.3). This sequence formed the opposite wall to block M-I in the binding pocket. The last wall of the surface depression (Block M-VI) lies between β8 and α7, with a consensus sequence **PG** V/P **P**x**E** M/L (Figure 6A, orange; Figure S7, lineage 1.3) and was highly conserved across all lineages. This block included the PG conserved sequence (P_148_G_149_, 3KBQ numbering), which was also found in MogA and Gephyrin (P_109_G_110_), and that was involved in phosphate binding (Figure 6A orange and 6B) [39,40]. Block M-VII represented a small consensus sequence (PxL) that identified out the end of the MocF domain (Figure 6B, dark grey; Figure S7).

All the above described blocks are also compatible with the recently described results in which MocF domain, called COG1058 by Raffaelli’s group [11], is endowed with a novel Co^2+^- and K^+^-dependent ADP-ribose (APDr) pyrophosphatase activity. In fact, ADPr docking in 3KBQ renders a similar picture (Figure 6C and D) to that obtained for Moco (Figure 6A and B), but with different interactions between the protein and the ligand. D75 (Block M-III) seemed to be involved in pyrophosphate binding, whereas E11 (Block M-I) and D44 (Block M-II) seemed to be related to adenosine binding (Figure 6C and D). In addition, P148 and G149 (Block M-VI) were probably associated with the ribose moiety. The MolDock scoring function from Molegro Virtual Docker [33], which is derived from the PLP (Piecewise Linear Potential) scoring function [42], was used to obtain the binding energy of Moco and ADPr to the MocF binding site shown in Figure 6. The values of -87.612 kcal/mol and -117.923 kcal/mol were obtained, respectively. Although Moco binding energy seems to be lower than that of ADPr, when the LE1 function, which represents the Ligand Efficiency (MolDock Score divided by heavy atoms count), was considered; both compounds showed similar values (-3.129 *vs* -3.275, respectively). 

No information on the structure of the interdomain segment is available since no crystals of two-domain PncCs have been described. However, two relatively conserved blocks have been identified (Figure S5; Figures S7-S8). The first interdomain block i-I had a consensus sequence GhG**E**S, which in the archaea lineage 1.3 is followed with a conserved xh**AP**. The block i-II was conserved in all lineages with a consensus sequence EVxhR/Kh, except for lineage 1.3. 

Structural analysis of PncC/CinA domain, using crystallized *Agrobacterium tumefaciens* CinA (PDB code 2A9S) (Figure 7A, Figures S6-S8, Lineage 2.2.3.1), showed nine conserved blocks (blocks P-I to P-IX) lining the walls of a cavity found on the surface of the molecule, which has been described as a putative NMN binding site [7]. Block P-I was located between β1 and α2 (Figure S7, Lineage 2.2.3.1) and its sequence (**AES**CTG**G**) was highly conserved in all lineages, including the inactive single-domain form of the enzyme (Figure S6, Lineage 2.2.2.2). These residues were located at the bottom of the active site pocket (Figure 7A, red). Residue S31 (2A9S numbering) has been proposed to interact with the NMN amide group (Figure 7B) [7]. This residue was replaced by alanine in the non-functional two-domain enzymes of lineage 2.2.1.2 (consensus sequence E Q/A C/G S/T G/A G, Figure 7C) and in the eukaryotic enzymes of lineage 2.2.3.4 (Figure 7C; Figures S6-S8), with the consensus sequence SEAACGG. Block P-II contains two of the important residues of NMN deamidases, G46 and S48, inserted in the consensus sequence **G** A/S S (Figure 7A, green). These two residues, which formed one of the walls of the cavity, belonged to subunit A, while the rest of residues forming the pocket were from subunit B (Figure 7A). G46 and S48 are not present in the inactive one-domain enzymes (Lineage 2.2.2.2, sequence **DTP**, Figure 7C), or in the inactive two-domains enzymes (lineage 2.2.1.2, sequence **A** D/P E/Q) (Figure 7C). Block P-III (Figure 7A, blue) covered a broad surface of the active site between β2 and α3 (Figure S7, lineage 2.2.3.1), and included two of the strictly conserved residues related to NMN deamidase activity, Y58 and K63 (**Y**xNxx**K**; Figure 7C; Figures S6-S8). In the enzymes of lineage 2.2.2.2, such as one-domain inactive *E. coli* YdeJ, two point mutations (Y58F and N60D) appeared to be responsible of the enzyme inactivity (Figure 7C). However, in the two domain non-functional enzymes of lineage 2.2.1.2, this block has disappeared, probably due to a deletion of a number of residues, as shown in Figure S1. Fungal CinA proteins of lineage 2.2.3.4 also showed a particular fingerprint in this block P-III (YS M/L K T/Q R), where they conserved the tyrosine but not the lysine (Figure 7C). Residues of block P-IV were found between α4 and α5, and covered the outer most part of the surface depression (Figure 7A, yellow). The sequence **G** A/V V S was conserved in all lineages, except in the inactive enzymes (sequence S/T AVS in homologues to YdeJ, G/E x V/L S/A in YfaY homologues, and MGPS in eukaryotic CinA; Figure 7C). Block P-V, despite being relatively conserved across NMN deamidases (**M**Ax**G**x), was not involved in active site formation (Figure 6C, pink). However, this block might have a structural function in the protein, since it was not found in two-domain inactive enzymes such as *E. coli* YfaY (Figure S1, Figures S6-S8). Block P-VI residues between β3 and β4 formed a wall of the active center facing Blocks P-II and P-III. This was one of the most conserved blocks (Figure 7A, magenta; Figure 7C) of the CinA domain. Most enzymes shared the S/T G I/V **AGP**x**GG** sequence, in which S/T105 and G106 were probably interacting with the amino group of NMN [7] (Figure 7B). This sequence contrasted with those of inactive enzymes of lineage 2.2.1.2 (S/VxxxxxE/H) and lineage 2.2.2.2 (S/T GYGGP) (Figure 7C; Figure S6). A strictly conserved glycine (G120) was found in block P-VII (KPV**G**TV) (Figure S6); which, as shown in Figure 7A (orange), was near to the binding pocket, close to the Block P-IV. This glycine was conserved in the inactive one-domain enzymes of lineage 2.2.2.2, however the whole block is absent in two-domain inactive PncCs (Figure S6, Lineage 2.2.1.2). Closing the active site pocket, the conserved R145 of block P-VIII was located in the last α-helix of the protein (Figure 7A, cyan), whose consensus sequence was Rxx V/I R (Figure S6). This residue might have a role in the binding of phosphate group (Figure 7B) and was not conserved in the inactive lineages 2.2.2.2 and 2.2.1.2 (Figure 7C). Finally, all NMN deamidases share a common sequence at the long and curved last α-helix of the protein with the fingerprint **L**xx**L**xxx**L** (Figure 7A, brown; Figure S6). This sequence appeared far from the active center, however might have a function in maintaining the structure of the PncC domain. The MolDock score (-122.053 kcal/moL) and ligand efficiency LE1 (-5.548) calculated for NMN to this binding site were also in the range of the above values for ADPr and Moco.

## Discussion

Nicotinamide adenine dinucleotide is an ancient and ubiquitous metabolite, which plays a crucial role linking biosynthetic pathways (acting as a cofactor of numerous enzymes) and regulatory pathways (acting as substrate for numerous protein- and nucleic acid-modifying reactions). Regardless the origin of these NAD^+^-degrading enzymes, there are several enzymes involved in NAD^+^ regeneration according to the Pyridine Nucleotide Cycle and NAD^+^ biosynthetic pathways shown in Figure 1. Among them, the enzyme nicotinamide mononucleotide (NMN) deamidase plays an important role, especially in microorganisms such as *Oceanobacillus iheyensis*, where no other enzymes, such as NadV, NadM or NadR are present (Figure 1), relying the fine control of NMN levels to it, in order to maintain the RecA DNA ligase activity [4].

In addition, NMN deamidase activity in this deep-sea microorganism runs in parallel with another key enzyme in the PNC pathway, nicotinamidase or PncA (Figure 1). We have previously investigated the distinct structural and biochemical characteristics of PncA [16]. In the current study, a comprehensive bioinformatic analysis of the entire NMN deamidase family was performed, and the evolutionary, structural and functional relationships of its members have been identified. These enzymes were mainly found in bacteria, with a few examples (3 putative sequences) in fungi (Figure 4). Interestingly, their structural alignment revealed, for the first time, that in addition to the four previously anticipated classes (active/inactive CinA, active/inactive CinA), a new domain architecture was also observed (Figure 5) [7]. This new domain architecture included several types of N-terminal extensions of variable length coupled to an active CinA domain.

A detailed phylogenetic analysis allowed us to classify NMN deamidases into 12 lineages, which have arisen from multiple evolutionary events, including domain fusion, gene duplication, horizontal transfers, gene decay and domain deletion. The last event could be the case of Lineage 1.3, whereupon archaea lost the CinA domain probably through a deletion after obtaining a two-domain functional PncC by horizontal gene transfer from a Firmicutes of Lineage 1.2 (Figure 5). Of note was the gene decay of Lineage 2.2.1.2 (Figure 7C, Figure S8, Lineage 2.2.1.2), represented by *E. coli* YfaY, where only block P-I, P-VIII and P-IX are relatively conserved, while the rest of them strongly differed from the CinA domain, indicating a massive accumulation of mutations or, more plausibly, a insertion of a mobile genetic element in the middle of CinA motif. A milder form of gene decay seems to have occurred in Lineage 2.2.2.2 (Figure 7C, Figure S8, lineage 2.2.2.2), represented by *E. coli* YdeJ, where blocks related to phosphate binding (P-II and P-VIII) and the tyrosine of block P-III were mutated. A particular case is also Lineage 2.2.3.4 (Figure 7C, Lineage 2.2.3.4), where it can be hypothesized that only two single mutations, one in block P-I (A→S) and one in block P-III (R→K) seems to be sufficient to recover activity in these fungal CinA.

Finally, the present work revealed some important gaps in the study of these NMN deamidases. Firstly, this work highlights the need for crystals structure of a functional two-domain PncC to be compared with that of one MocF domain enzymes (like 3KBQ) or one domain CinA enzymes (like 2A9S). In addition, the role of the interdomain sequence in the final structure of two-domain PncCs is still unknown. Moreover, further experiments using MPT-AMP as possible substrate for MocF domain are required to fully explore the catalytic role of MocF-containing enzymes. The recent work assigning a pyrophosphatase function towards ADPr to those enzymes [11] establishes a clear parallelism between the cleavage of the pyrophosphate bond found in ADPr and that found in the advanced intermediate MPT-AMP used as substrate by structurally similar proteins, such as MoeA and the eukaryotic orthologous gephyrinE or plant Cnx1E [11]. In fact, the COG1058 gene product of *A. tumefaciens* (Uniprot code: Q7CYN5) and *S. oneidensis* COG1058/PncC (Uniprot code: Q8EK32) were only active towards ADPr in the presence of Co^2+^ and K^+^ ions, but not with Mg^2+^, which also required Molybdate (MoO_4_^2-^) in the MPT-AMP cleavage carried out by MoeA, gephyrin and Cnx1 to give Moco [13]. The above possible dual substrate (ADPr and MPT-AMP) activity in MocF domain was also supported by the docking binding energies and ligand efficiencies shown in this paper. Answers to these open questions will hopefully be found in the near future when new MocF/CinA enzymes are characterized.

## Supporting Information

Figure S1 **Multiple sequence alignment for *O. iheyensis* (OiPncC) and related nicotinamide mononucleotide deamidases.** ESPript outputs [21] obtained with the sequences from *Oceanobacillus iheyensis* PncC (functional two-domains, UniProt code: Q8EQR8), *Shewanella oneidensis* PncC (functional two-domains, UniProt code: Q8EK32), *Agrobacterium tumefaciens* PncC (functional one-domain, UniProt code: A9CJ26), *Escherichia coli* YGAD PncC (functional one-domain, UniProt code: P0A6G3), *E. coli* YDEJ PncC (non-functional one-domain, UniProt code: P31131) and *E. coli* YFAY PncC (non-functional two-domains, UniProt code: P77808) were aligned with CLUSTAL-W [20]. Residues strictly conserved across NMN deamidase enzymes have a dark background. Symbols above blocks of sequences represent the secondary structure of *O. iheyensis* NMN deamidase, springs represent helices and arrows represent β-strands. Conserved residues of the MocF domain are marked with a blue triangle. Conserved residues of the inter-domain segment are marked with a green triangle. Conserved residues of the CinA domain are marked with a red triangle.
(TIF)

Figure S2 **SDS-PAGE of the OiPncC purification.** M: molecular weight standards (New England Biolabs: P7708S). Lane 1: cell extract after disruption. Lane 2: cell extract after 50 kDa tangential ultrafiltration. Lane 3: OiPncC after HisTrap column step (purified protein is about 47 kDa). Each lane contained 20 µg of protein.
(TIF)

Figure S3 **Enzymatic activity of OiPncC.** A) Assayed by HPLC. Aliquots of the reaction were removed and stopped at 0 minutes (solid line), 10 minutes (dashed-line) and 15 minutes (dotted line) of the reaction course. B) Assayed by enzyme-coupled assay using glutamate dehydrogenase. The standard reaction conditions at 37 °C were used.
(TIF)

Figure S4 **Effect of NMN concentration on OiPncC activity.** A) Measured by HPLC under the standard reaction conditions at 37 °C and increasing concentrations of NMN (0.01 mM to 1 mM). B) Measured by the enzyme-coupled spectrophotometric method under the standard reaction conditions at 37 °C and increasing concentrations of NMN (0.01 mM to 1 mM).
(TIF)

Figure S5 **Conserved blocks in the MocF domain and interdomain segment in the different lineages.** Red background indicates strictly conserved amino acids; orange background indicates conserved amino acids; “c”, a charged residue; “h”, a hydrophobic residue; “p”, a polar residue; and “x”, any residue. Alternative amino acids at a given position are shown within brackets.
(TIF)

Figure S6 **Conserved blocks in the CinA domain in the different lineages.** Red background indicates strictly conserved amino acids; orange background indicates conserved amino acids; “c”, a charged residue; “h”, a hydrophobic residue; “p”, a polar residue; and “x”, any residue. Alternative amino acids at a given position are shown within brackets.
(TIF)

Figure S7 **ESPript outputs obtained with the sequences from the different lineages.** Residues strictly conserved across NMN deamidase enzymes have a red background. Symbols above blocks of sequences represent the secondary structure of the most representative enzyme from each lineage, springs represent helices and arrows represent β-strands. Conserved blocks are marked under the corresponding sequences for each lineage.
(PDF)

Figure S8 **WEBLOGO outputs obtained from the above alignment of the sequences from the different lineages.** Conserved blocks are marked under the corresponding sequences for each lineage.
(PDF)

Table S1 **NMN deamidases used in the phylogenetic analysis.**
(PDF)
