# Supplementary figures and images for: New Insights into the Phylogeny and Molecular Classification of Nicotinamide Mononucleotide Deamidases
Source: PLoS One. 2013 Dec 5;8(12):e82705. doi: 10.1371/journal.pone.0082705 (PMC3855486; doi:10.1371/journal.pone.0082705)

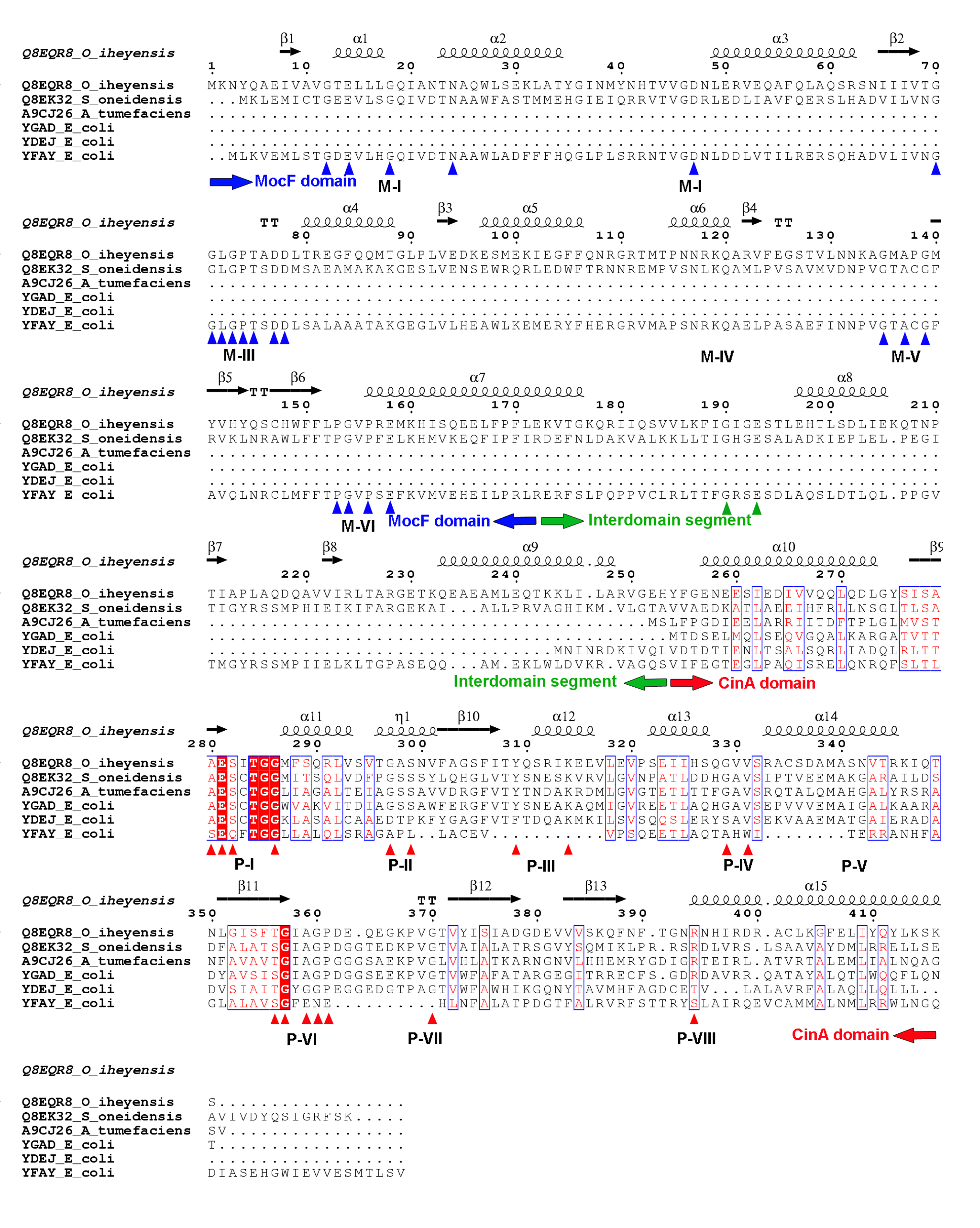

Supplement: Figure S1 — Multiple sequence alignment for O. iheyensis (OiPncC) and related nicotinamide mononucleotide deamidases. ESPript outputs [21] obtained with the sequences from Oceanobacillus iheyensis PncC (functional two-domains, UniProt code: Q8EQR8), Shewanella oneidensis PncC (functional two-domains, UniProt code: Q8EK32), Agrobacterium tumefaciens PncC (functional one-domain, UniProt code: A9CJ26), Escherichia coli YGAD PncC (functional one-domain, UniProt code: P0A6G3), E. coli YDEJ PncC (non-functional one-domain, UniProt code: P31131) and E. coli YFAY PncC (non-functional two-domains, UniProt code: P77808) were aligned with CLUSTAL-W [20]. Residues strictly conserved across NMN deamidase enzymes have a dark background. Symbols above blocks of sequences represent the secondary structure of O. iheyensis NMN deamidase, springs represent helices and arrows represent β-strands. Conserved residues of the MocF domain are marked with a blue triangle. Conserved residues of the inter-domain segment are marked with a green triangle. Conserved residues of the CinA domain are marked with a red triangle. (TIF) [file pone.0082705.s001.tif]

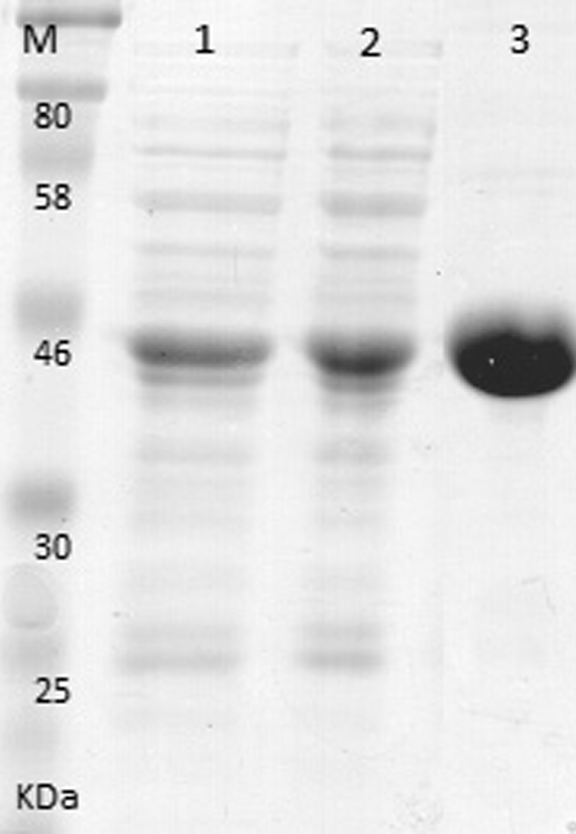

Supplement: Figure S2 — SDS-PAGE of the OiPncC purification. M: molecular weight standards (New England Biolabs: P7708S). Lane 1: cell extract after disruption. Lane 2: cell extract after 50 kDa tangential ultrafiltration. Lane 3: OiPncC after HisTrap column step (purified protein is about 47 kDa). Each lane contained 20 µg of protein. (TIF) [file pone.0082705.s002.tif]

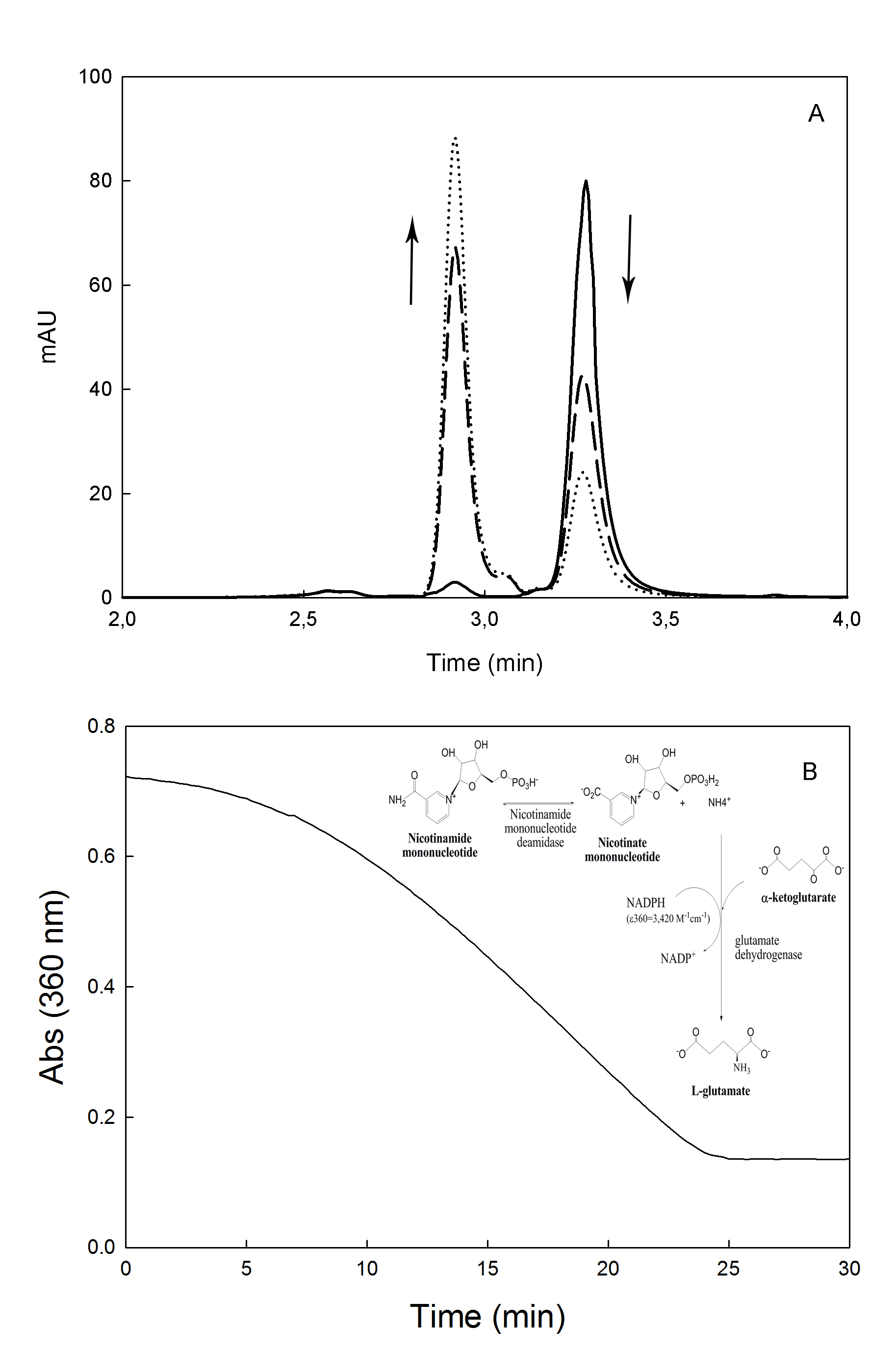

Supplement: Figure S3 — Enzymatic activity of OiPncC. A) Assayed by HPLC. Aliquots of the reaction were removed and stopped at 0 minutes (solid line), 10 minutes (dashed-line) and 15 minutes (dotted line) of the reaction course. B) Assayed by enzyme-coupled assay using glutamate dehydrogenase. The standard reaction conditions at 37 °C were used. (TIF) [file pone.0082705.s003.tif]

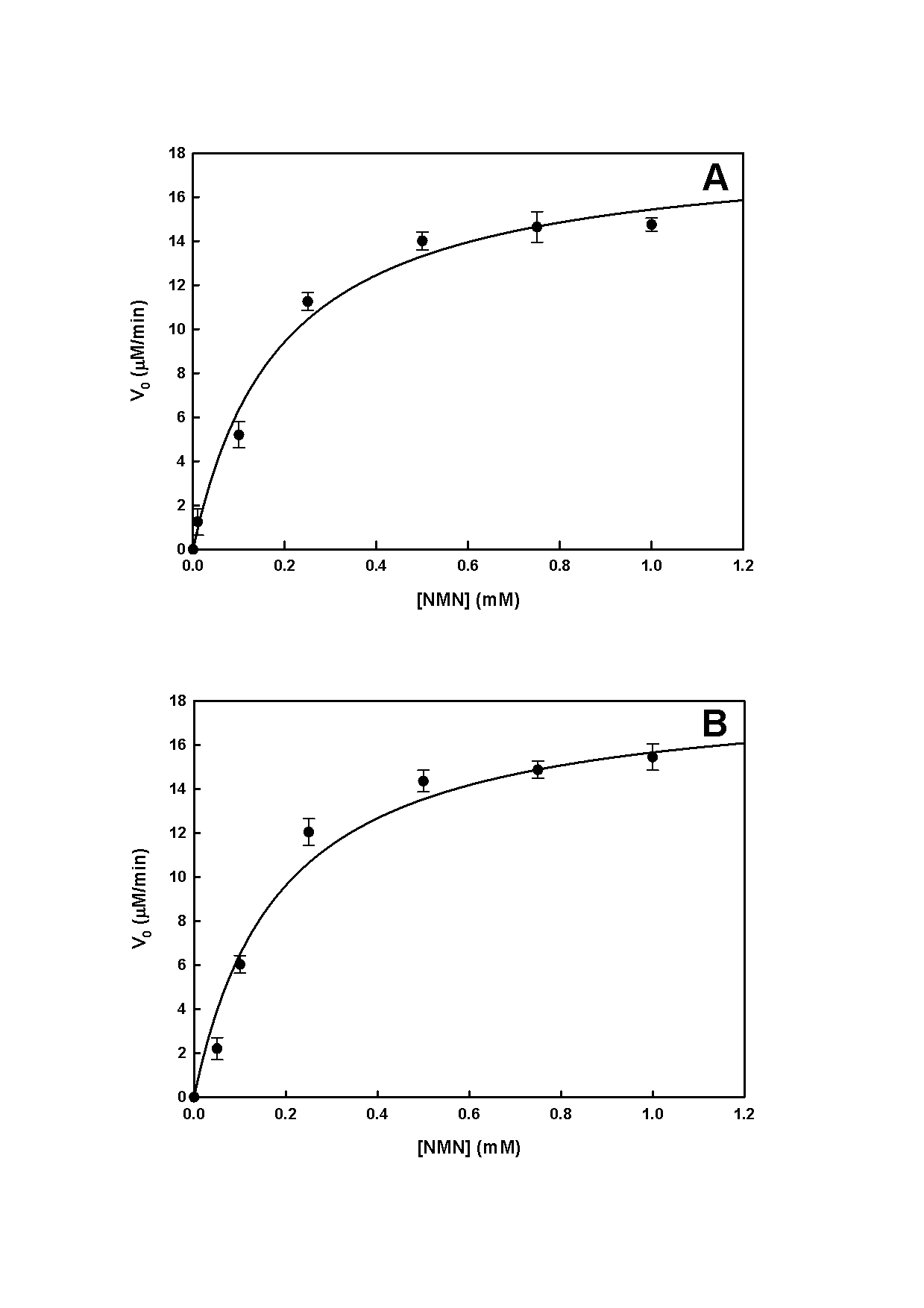

Supplement: Figure S4 — Effect of NMN concentration on OiPncC activity. A) Measured by HPLC under the standard reaction conditions at 37 °C and increasing concentrations of NMN (0.01 mM to 1 mM). B) Measured by the enzyme-coupled spectrophotometric method under the standard reaction conditions at 37 °C and increasing concentrations of NMN (0.01 mM to 1 mM). (TIF) [file pone.0082705.s004.tif]

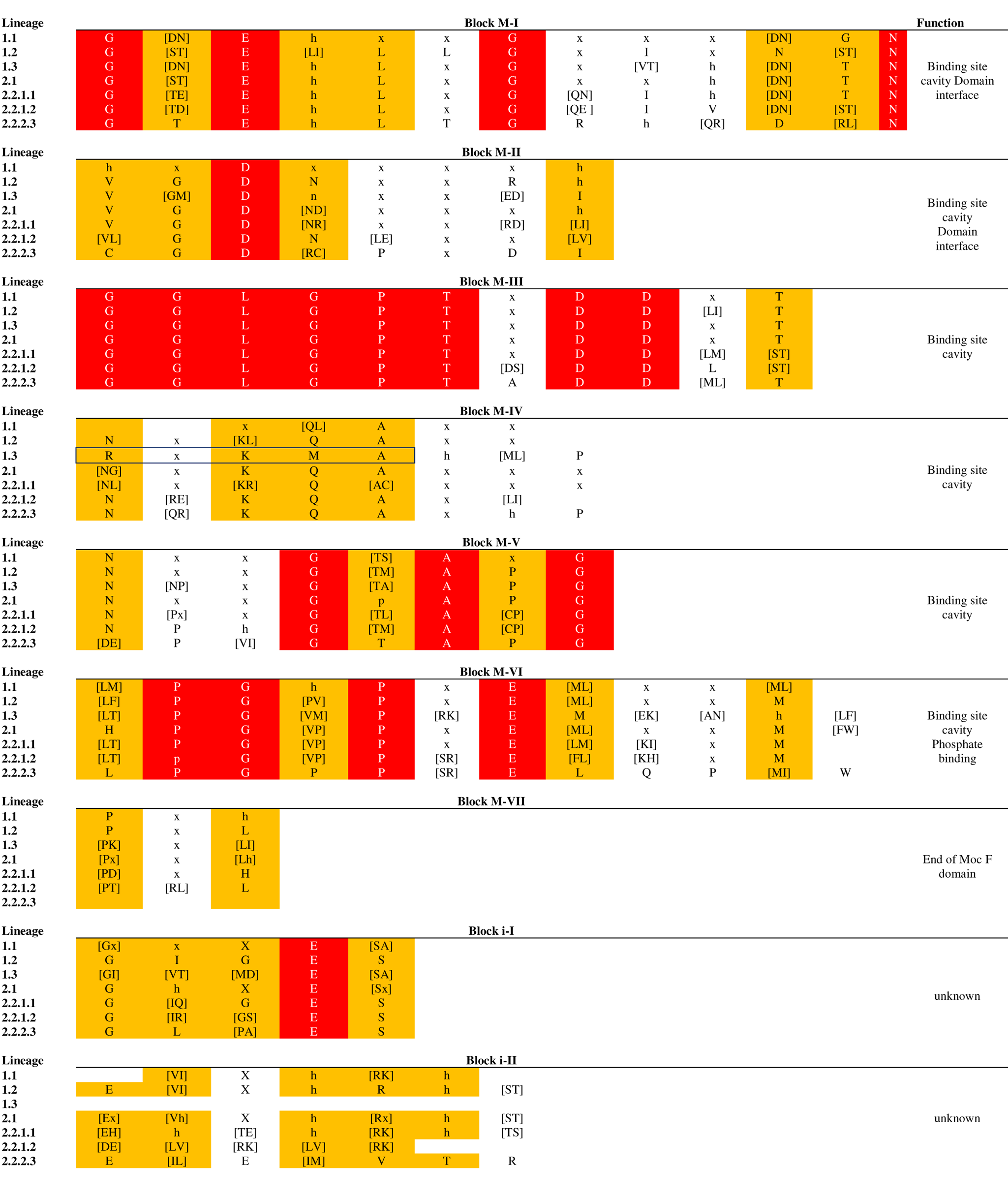

Supplement: Figure S5 — Conserved blocks in the MocF domain and interdomain segment in the different lineages. Red background indicates strictly conserved amino acids; orange background indicates conserved amino acids; “c”, a charged residue; “h”, a hydrophobic residue; “p”, a polar residue; and “x”, any residue. Alternative amino acids at a given position are shown within brackets. (TIF) [file pone.0082705.s005.tif]

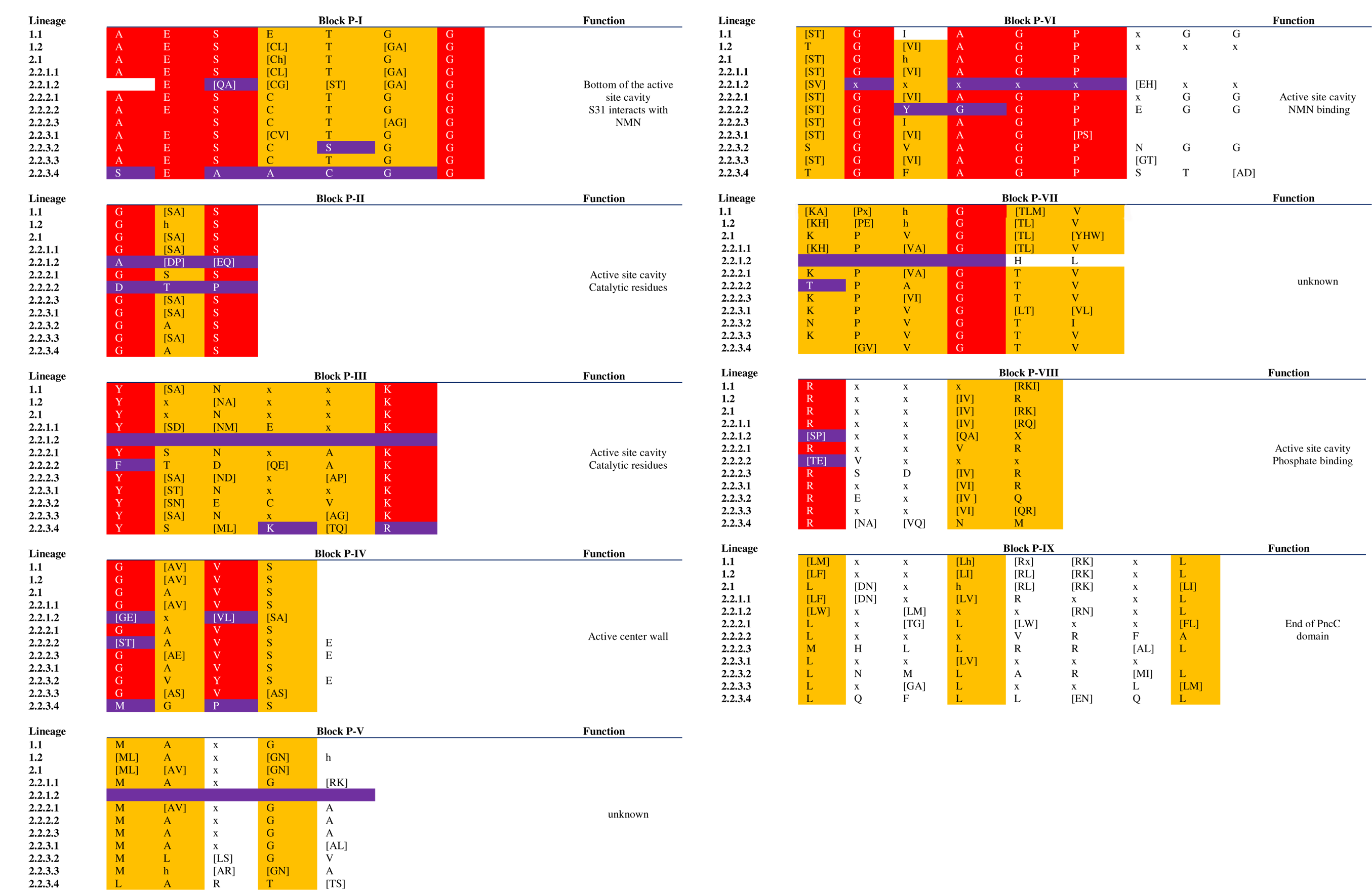

Supplement: Figure S6 — Conserved blocks in the CinA domain in the different lineages. Red background indicates strictly conserved amino acids; orange background indicates conserved amino acids; “c”, a charged residue; “h”, a hydrophobic residue; “p”, a polar residue; and “x”, any residue. Alternative amino acids at a given position are shown within brackets. (TIF) [file pone.0082705.s006.tif]
