# Supplementary material for: New Insights into the Phylogeny and Molecular Classification of Nicotinamide Mononucleotide Deamidases
Source: PLoS One. 2013 Dec 5;8(12):e82705. doi: 10.1371/journal.pone.0082705 (PMC3855486; doi:10.1371/journal.pone.0082705)
Supplement: Figure S7 — ESPript outputs obtained with the sequences from the different lineages. Residues strictly conserved across NMN deamidase enzymes have a red background. Symbols above blocks of sequences represent the secondary structure of the most representative enzyme from each lineage, springs represent helices and arrows represent β-strands. Conserved blocks are marked under the corresponding sequences for each lineage. (PDF) [file pone.0082705.s007.pdf]

Lineage 1.1

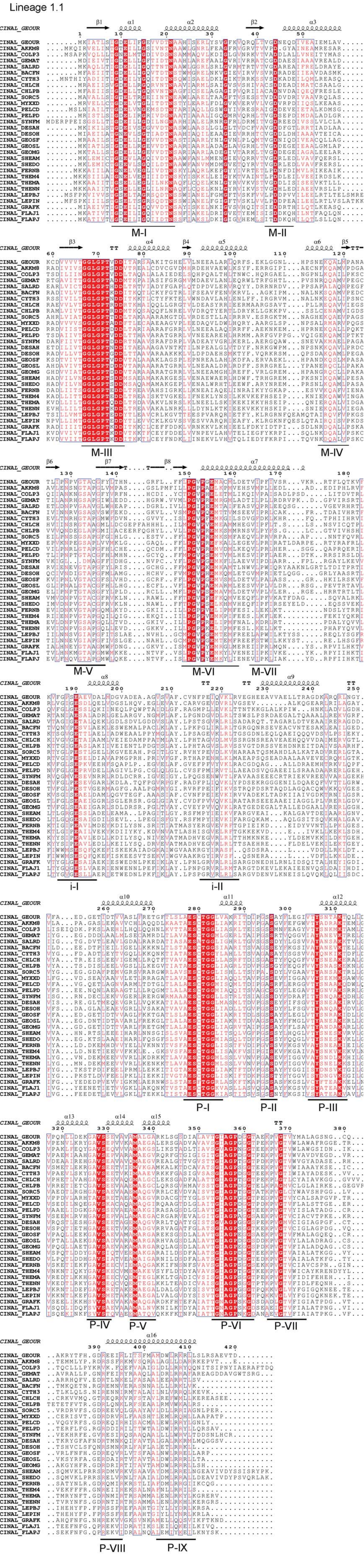

M-I
M-II
M-III
M-IV
M-V
M-VI
M-VII
i-I
i-II
P-I
P-II
P-III
P-IV
P-V
P-VI
P-VII
P-VIII
P-IX

Lineage 1.2

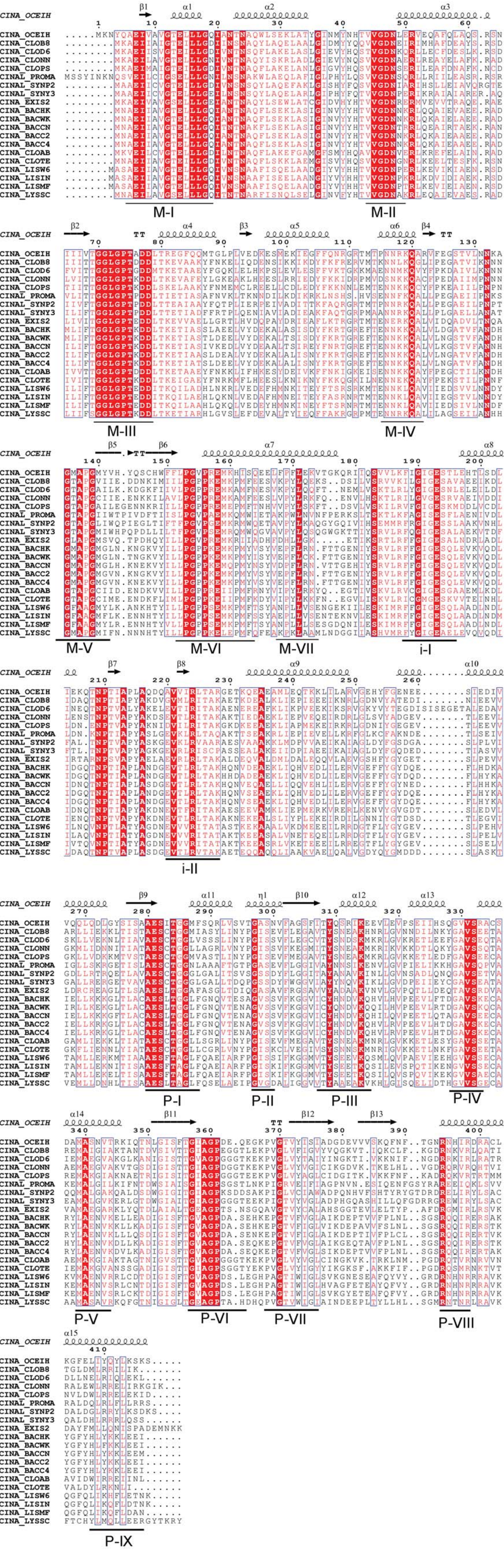
M-I
M-II
M-III
M-IV
M-V
M-VI
M-VII
i-I
i-II
P-I
P-II
P-III
P-IV
P-V
P-VI
P-VII
P-VIII
P-IX

Lineage 1.3

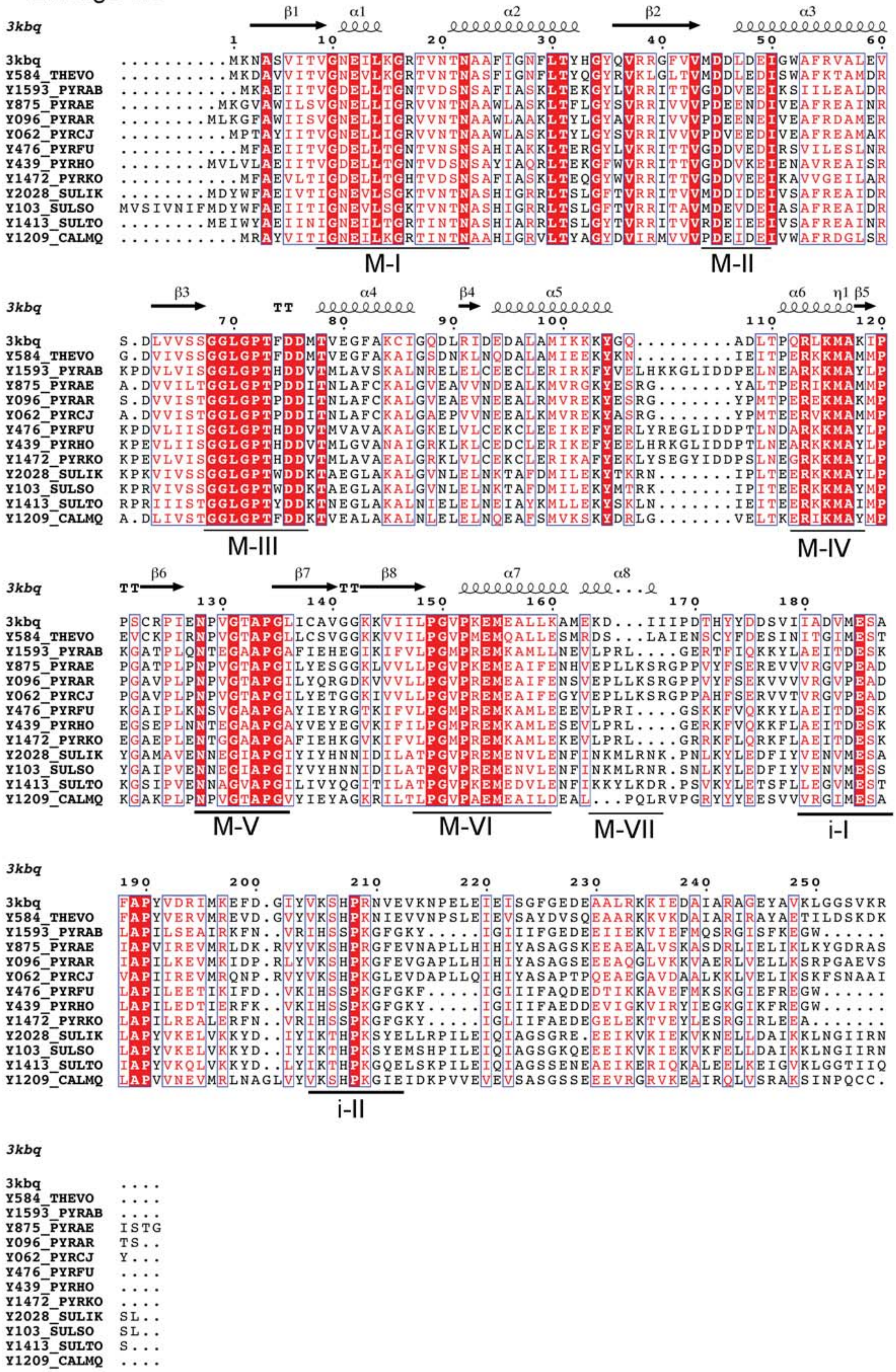
3kbq
β1 α1 α2 β2 α3
1 10 20 30 40 50 60
3kbq ..........MKNASVITVGNEILKGRTVNTNAAFIGNFLTYHGYQVRRGFVVMDDLDEIGWAFRVALEV
Y584_THEVO ..........MKDAVVITVGNEVLKGRTVNTNASFIGNFLTYQGYRVKLGLTVMDDLEDISWAFKTAMDR
Y1593_PYRAB ..........MKAEIITVGDELLTGNTVDSNSAFIASKLTEKGYLVRRITTVGDDVEEIKSIILEALDR
Y875_PYRAE .........MKGVAWILSVGNELLIGRVVNTNAAWLASKLTFLGYSVRRIVVVPDEENDIVEAFREAINR
Y096_PYRAR ........MLKGFAWIISVGNELLIGRVVNTNAAWLAAKLTYLGYAVRRIVVVPDEENEIVEAFRDAMER
Y062_PYRCJ ..........MPTAYIITVGNELLIGRVVNTNAAWLASKLTYLGYSVRRIVVVPDVEEDIVEAFREAMAK
Y476_PYRFU ...........MFAEIITVGDELLTGNTVNSNSAHIAKKLTERGYLVKRITTVGDDVEDIRSVILESLNR
Y439_PYRHO ........MVLVLAEIITVGDELLTGNTVDSNSAYIAQRLTEKGFWVRRITTVGDDVKEIENAVREAISR
Y1472_PYRKO ...........MFAEVLTIGDELLTGHTVDSNSAFIASKLTEQGYWVRRITTVGDDVEEIKAVVGEILAR
Y2028_SULIK ........MDYWFAEIVTIGNEVLSGKTVNTNASHIGRRLTSLGFTVRRITVVMDDIDEIVSAFREAIDR
Y103_SULSO MVSIVNIFMDYWFAEIITIGNEVLSGKTVNTNASHIGRRLTSLGFTVRRITAVMDEVDEIASAFREAIDR
Y1413_SULTO ........MEIWYAEIINIGNEILTGRTINTNASHIARRLTSLGYTVRRITVVRDEIEEIVSAFREAINR
Y1209_CALMQ ..........MRAYVITIGNEILKGRTINTNAAHIGRVLTYAGYDVIRMVVVPDEIDEIVWAFRDGLSR
M-I M-II
3kbq
β3 TT α4 β4 α5 α6 η1 β5
70 80 90 100 110 120
3kbq S.DLVVSSGGLGPTFDDMTVEGFAKCIGQDLRIDEDALAMIKKKYGQ.........ADLTPQRLKMAKIP
Y584_THEVO G.DVIVSSGGLGPTFDDMTVEGFAKAIGSDNKLNQDALAMIEEKYKN.........IEITPERKKMAMMP
Y1593_PYRAB KPDVLVISGGLGPTHDDVTMLAVSKALNRELELCEECLERIRKFYVELHKKGLIDDPELNEARKKMAYLP
Y875_PYRAE A.DVVILTGGLGPTPDDITNLAFCKALGVEAVVNDEALKMVRGKYESRG.......YALTPERIKMAMMP
Y096_PYRAR S.DVVISTGGLGPTPDDITNLAFCKALGVEAEVNEEALRMVREKYESRG.......YPMTPEREKMAKMP
Y062_PYRCJ A.DVVISTGGLGPTPDDITNLAFCKALGAEPVVNEEALKMVREKYASRG.......YPMTEERVKMAMMP
Y476_PYRFU KPDVLIISGGLGPTHDDVTMVAVAKALGKELVLCEKCLEEIKEFYERLYREGLIDDPTLNDARKKMAYLP
Y439_PYRHO KPEVLIISGGLGPTHDDVTMLGVANAIGRKLKLCEDCLERIKEFYEELHRKGLIDDPTLNEARKKMAYLP
Y1472_PYRKO KPEVLVISGGLGPTHDDVTMLAVAEALGRKLVLCEKCLERIKAFYEKLYSEGYIDDPSLNEGRKKMAYLP
Y2028_SULIK KPKVIVSSGGLGPTWDDKTAEGLAKALGVNLELNKTAFDMILEKYTKRN.......IPLTEERKKMAYLP
Y103_SULSO KPRVIVSSGGLGPTWDDKTAEGLAKALGVNLELNKTAFDMILEKYMTRK.......IPITEERKKMAYMP
Y1413_SULTO RPRIIISTGGLGPTYDDKTNEGLAKALNIELELNEIAYKMLLEKYSKLN.......IEITEERKKMAIMP
Y1209_CALMQ A.DLIVSTGGLGPTFDDKTVEALAKALNLELELNQEAFSMVKSKYDRLG.......VELTKERIKMAYMP
M-III M-IV
3kbq
TT β6 β7 TT β8 α7 α8
130 140 150 160 170 180
3kbq PSCRPIENPVGTAPGLICAVGGKKVIILPGVPKEMEALLKAMEKD...IIIPDTHYYDDSVIIADVMESA
Y584_THEVO EVCKPIRNPVGTAPGLLCSVGGKKVVILPGVPMEMQALLESMRDS...LAIENSCYFDESINITGIMEST
Y1593_PYRAB KGATPLQNTEGAAPGAFIEHEGIKIFVLPGMPREMKAMLLNEVLPRL....GERTFIQKKYLAEITDESK
Y875_PYRAE PGATPLPNPVGTAPGILYESGGKLVVLLPGVPKEMEAIFENHVEPLLKSRGPPVYFSEREVVVRGVPEAD
Y096_PYRAR PGAVPLPNPVGTAPGILYQRGDKVVVLLPGVPREMEAIFENSVEPLLKSRGPPVYFSEKVVVVRGVPEAD
Y062_PYRCJ PGAVPLPNPVGTAPGILYETGGKIVVLLPGVPREMEAIFEGYVEPLLKSRGPPAHFSERVVTVRGVPEAD
Y476_PYRFU KGAIPLKNSVGAAPGAYIEYRGTKIFVLPGMPREMKAMLEEVLPRI....GSKKFVQKKYLAEITDESK
Y439_PYRHO EGSEPLNNTEGAAPGAYVEYEGVKIFILPGMPREMKAMLESEVLPRL....GERKFVQKKFLAEITDESK
Y1472_PYRKO EGAEPLENTGGAAPGAFIEHKGVKIFVLPGMPREMKAMLEKEVLPRL....GRRKFLQRKFLAEITDESK
Y2028_SULIK YGAMAVENNEGIAPGIYIYHNNIDILATPGVPREMENVLENFINKMLRNK.PNLKYLEDFIYVENVMESA
Y103_SULSO YGAIPVENNEGIAPGIYVYHNNIDILATPGVPREMENVLENFINKMLRNR.SNLKYLEDFIYVENVMESS
Y1413_SULTO KGSIPVENNAGVAPGILIVYQGITILATPGVPKEMEDVLENFIKKYLKDR.PSVKYLETSFLLEGVMEST
Y1209_CALMQ KGAKPLPNPVGTAPGVYIEYAGKRILTLPGVPAEMEAILDEAL...PQLRVPGRYYYEESVVVRGIMESA
M-V M-VI M-VII i-I
3kbq
190 200 210 220 230 240 250
3kbq FAPYVDRIMKEFD.GIYVKSHPRNVEVKNPELEIEISGFGEDEAALRKKIEDAIARAGEYAVKLGGSVKR
Y584_THEVO FAPYVERVMREVD.GVYVKSHPKNIEVVNPSLEIEVSAYDVSQEAARKKVKDAIARIRAYAETILDSKDK
Y1593_PYRAB LAPILSEAIRKFN..VRIHSSPKGFGKY.....IGIIIFGEDEEIIEKVIEFMQSRGISFKEGW......
Y875_PYRAE IAPVIREVMRLDK.RVYVKSHPRGFEVNAPLLHIHIYASAGSKEEAEALVSKASDRLIELIKLKYGDRAS
Y096_PYRAR IAPILKEVMKIDP.RLYVKSHPKGFEVGAPLLHIHIYASAGSEEAQGLVKKVAERLVELLKSRPGAEVS
Y062_PYRCJ VAPIIREVMRQNP.RVYVKSHPKGLEVDAPLLQIHIYASAPTPQEAEGAVDAALKKLVELIKSKFSNAAI
Y476_PYRFU LAPILEETIKIFD..VKIHSSPKGFGKF.....IGIIIFAQDEDTIKKAVEFMKSKGIEFREGW......
Y439_PYRHO LAPILEDTIERFK..VKIHSSPKGFGKY.....IGIIIFAEDDEVIGKVIRYIEGKGIKFREGW......
Y1472_PYRKO LAPILREALERFN..VRIHSSPKGFGKY.....IGLIIFAEDEGELEKTVEYLESRGIRLEEA.......
Y2028_SULIK LAPYVKELVKKYD..IYIKTHPKSYELLRPILEIQIAGSGRE.EEIKVKIEKVKNELLDAIKKLNGIIRN
Y103_SULSO LAPYVKELVKKYD..IYIKTHPKSYEMSHPILEIQIAGSGKQEEEIKVKIEKVKFELLDAIKKLNGIIRN
Y1413_SULTO IAPYVKQLVKKYD..LYIKTHPKGQELSKPILEIQIAGSSENEAEIKERIQKALEELKEIGVKLGGTIIQ
Y1209_CALMQ LAPVVNEVMRLNAGLVYVKSHPKGIEIDKPVVEVEVSASGSSEEEVRGRVKEAIRQLVSRAKSINPQCC.
i-II
3kbq
3kbq ....
Y584_THEVO ....
Y1593_PYRAB ....
Y875_PYRAE ISTG
Y096_PYRAR TS..
Y062_PYRCJ Y...
Y476_PYRFU ....
Y439_PYRHO ....
Y1472_PYRKO ....
Y2028_SULIK SL..
Y103_SULSO SL..
Y1413_SULTO S...
Y1209_CALMQ ....

Lineage 2.1

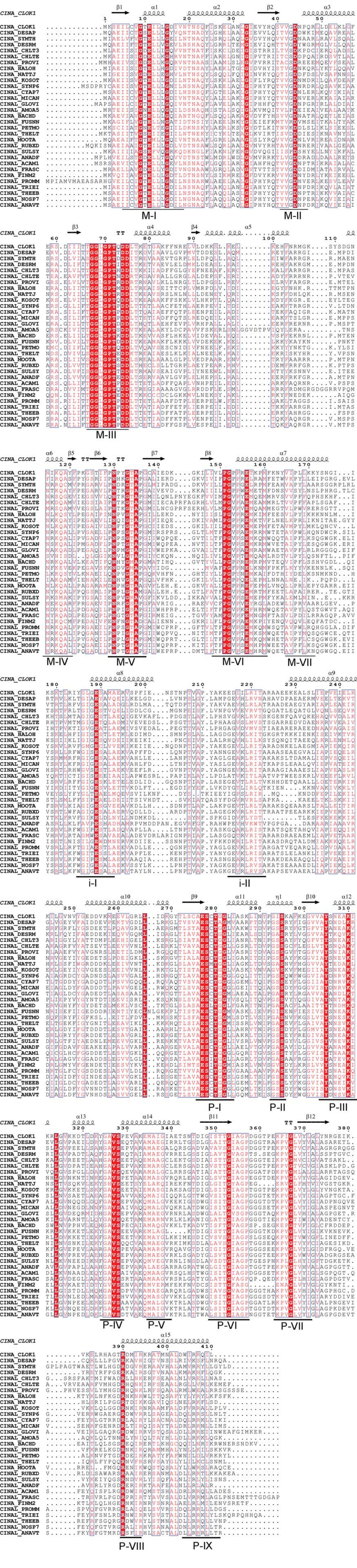
CINA_CLOK1
M-I
M-II
M-III
M-IV
M-V
M-VI
M-VII
i-I
i-II
P-I
P-II
P-III
P-IV
P-V
P-VI
P-VII
P-VIII
P-IX

Lineage 2.2.1.1

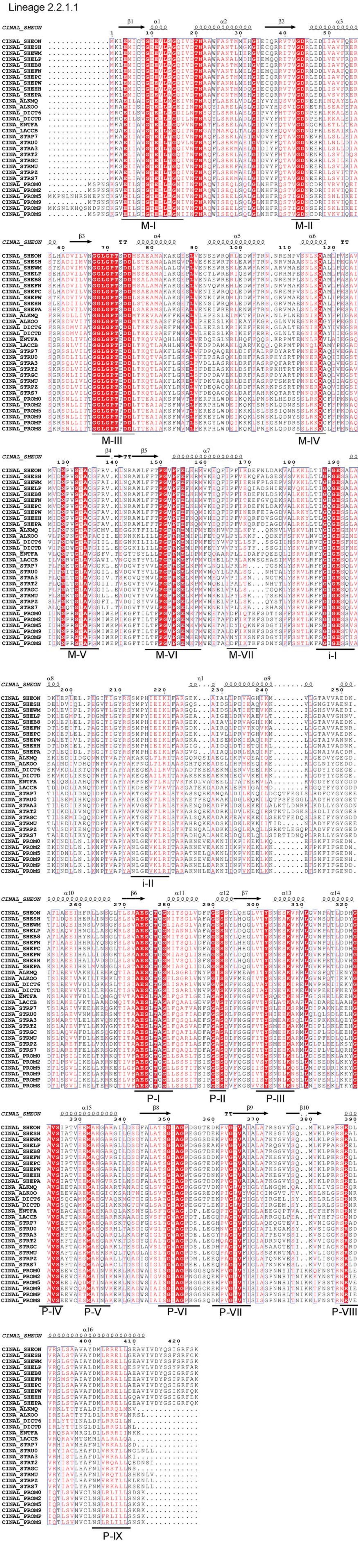

Lineage 2.2.1.2

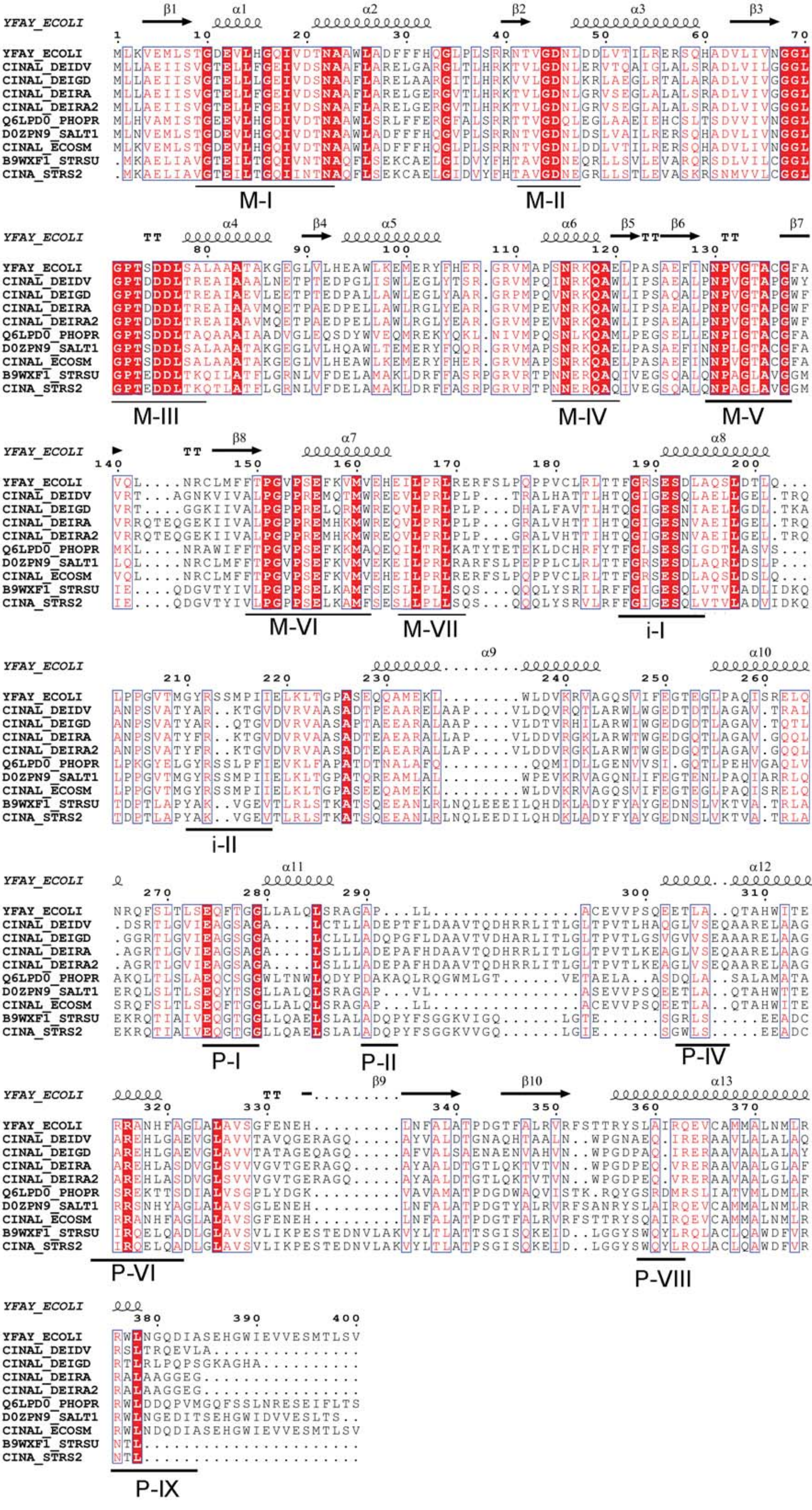

Lineage 2.2.2.1

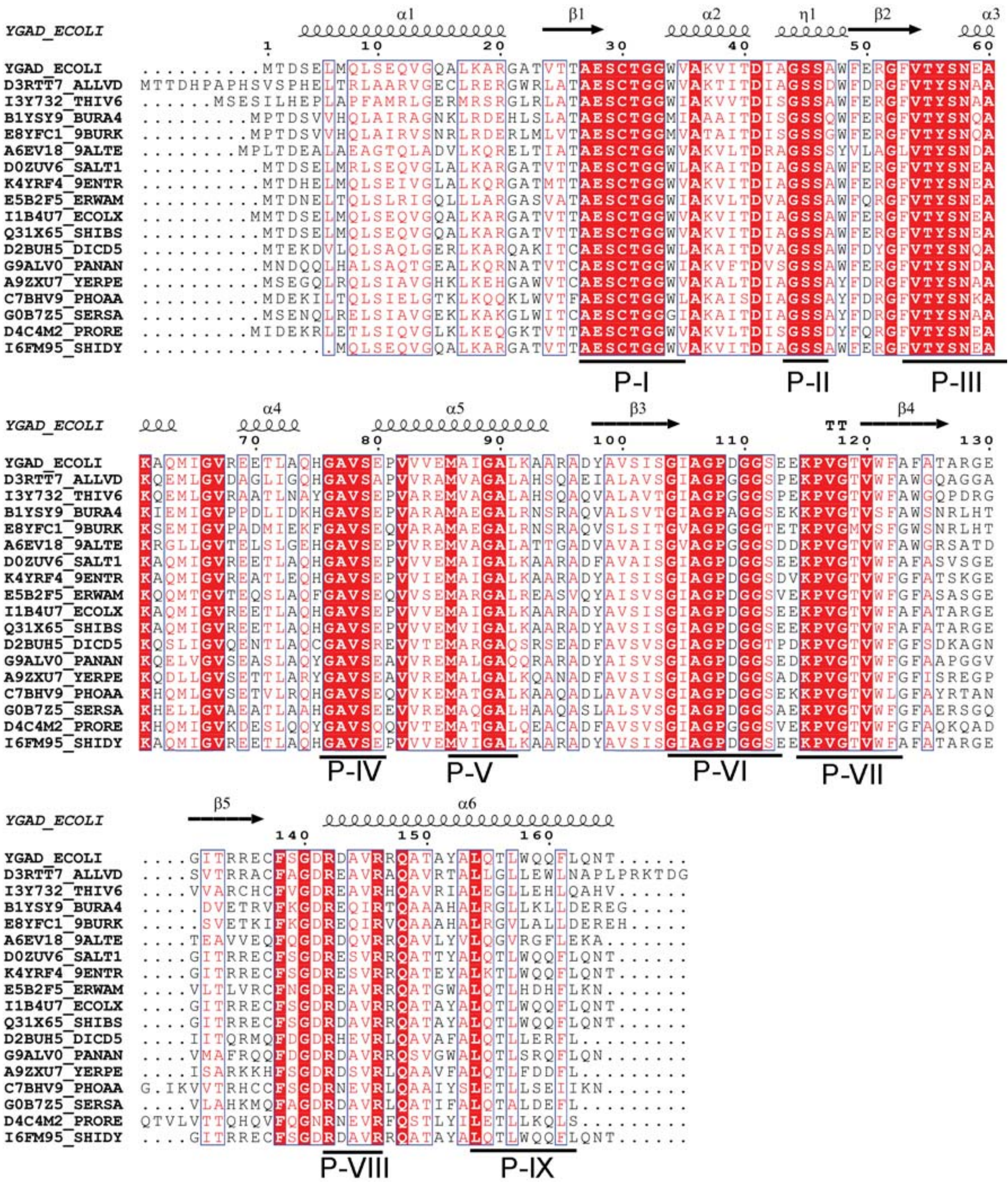

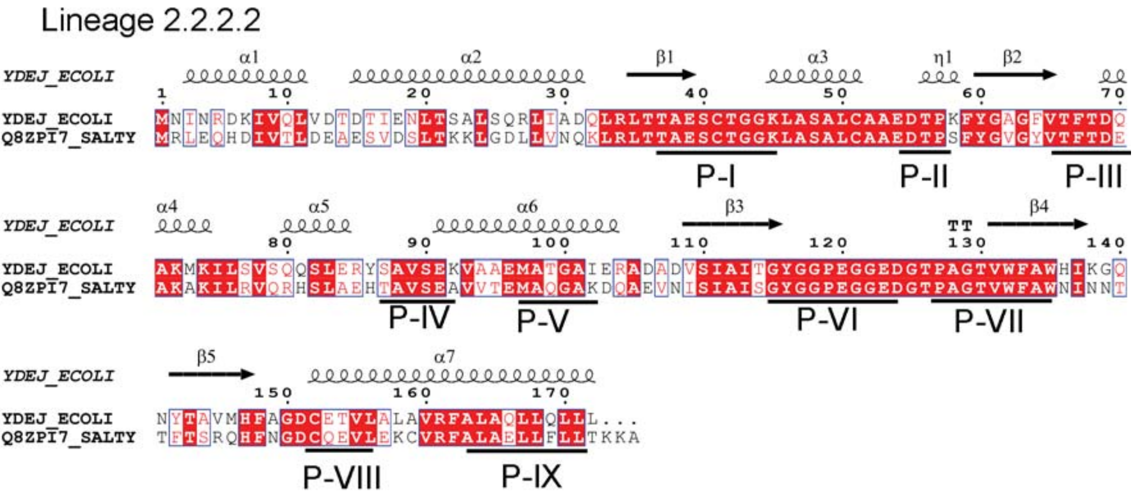

Lineage 2.2.2.2
YDEJ_ECOLI
α1
α2
β1
α3
η1
β2
1
10
20
30
40
50
60
70
YDEJ_ECOLI MNINRDKIVQLVDTDTIENLTSALSQRLIADQLRLTTAESCTGGKLASALCAAEDTPKFYGAGFVTFTDQ
Q8ZPI7_SALTY MRLEQHDIVTLDEAESVDSLTKKLGDLLVNQKLRLTTAESCTGGKLASALCAAEDTPSFYGVGYVTFTDE
P-I
P-II
P-III
α4
α5
α6
β3
TT
β4
80
90
100
110
120
130
140
YDEJ_ECOLI AKMKILSVSQQSLERYSAVSEKVAAEMATGAIERADADVSIAITGYGGPEGGEDGTPAGTVWFAWHIKGQ
Q8ZPI7_SALTY AKAKILRVQRHSLAEHTAVSEAVVTEMAQGAKDQAEVNISIAISGYGGPEGGEDGTPAGTVWFAWNINNT
P-IV
P-V
P-VI
P-VII
β5
α7
150
160
170
YDEJ_ECOLI NYTAVMHFAGDCETVLALAVRFALAQLLQLLL...
Q8ZPI7_SALTY TFTSRQHFNGDCQEVLEKCVRFALAELLFLLTKKA
P-VIII
P-IX

Lineage 2.2.2.3

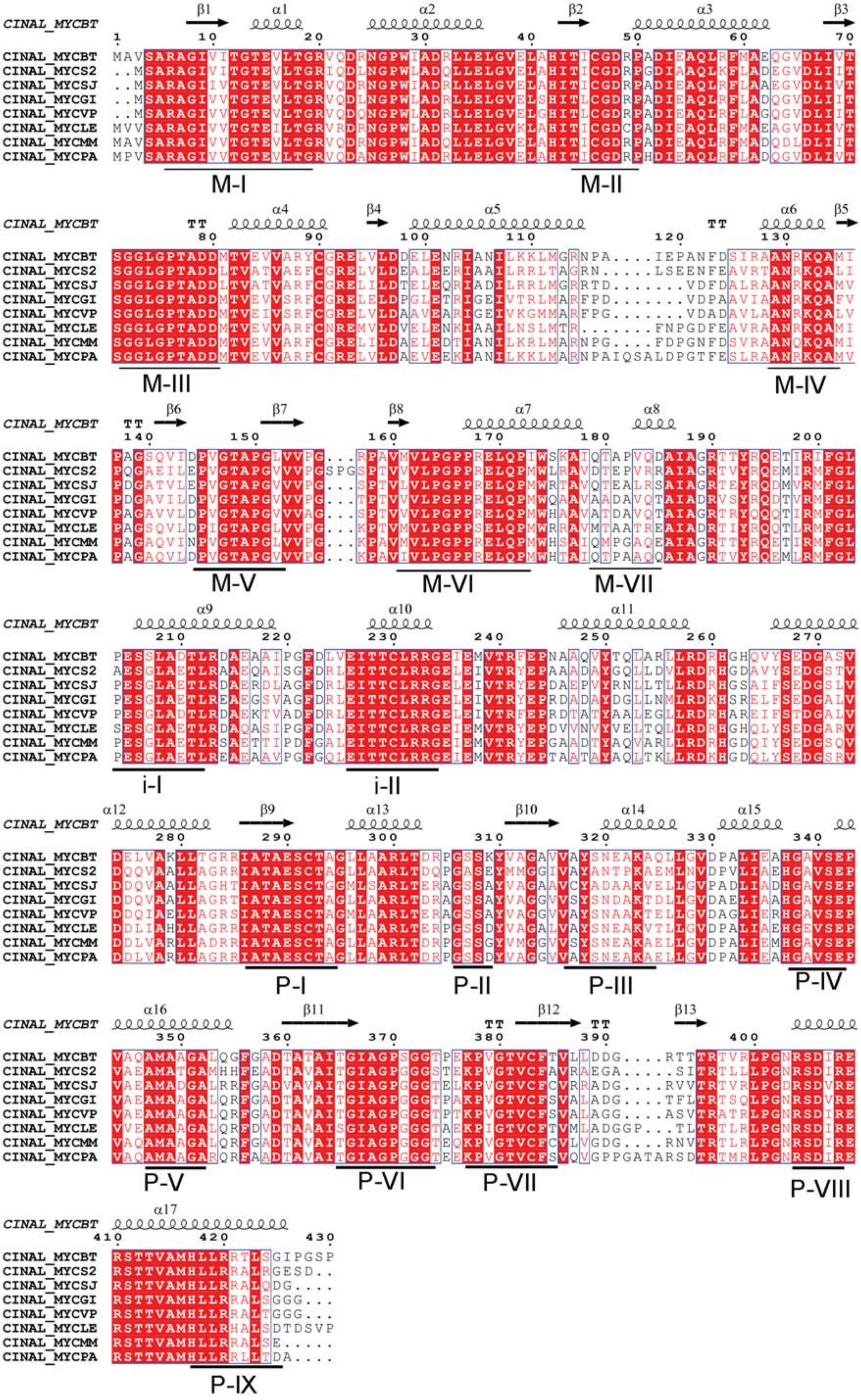

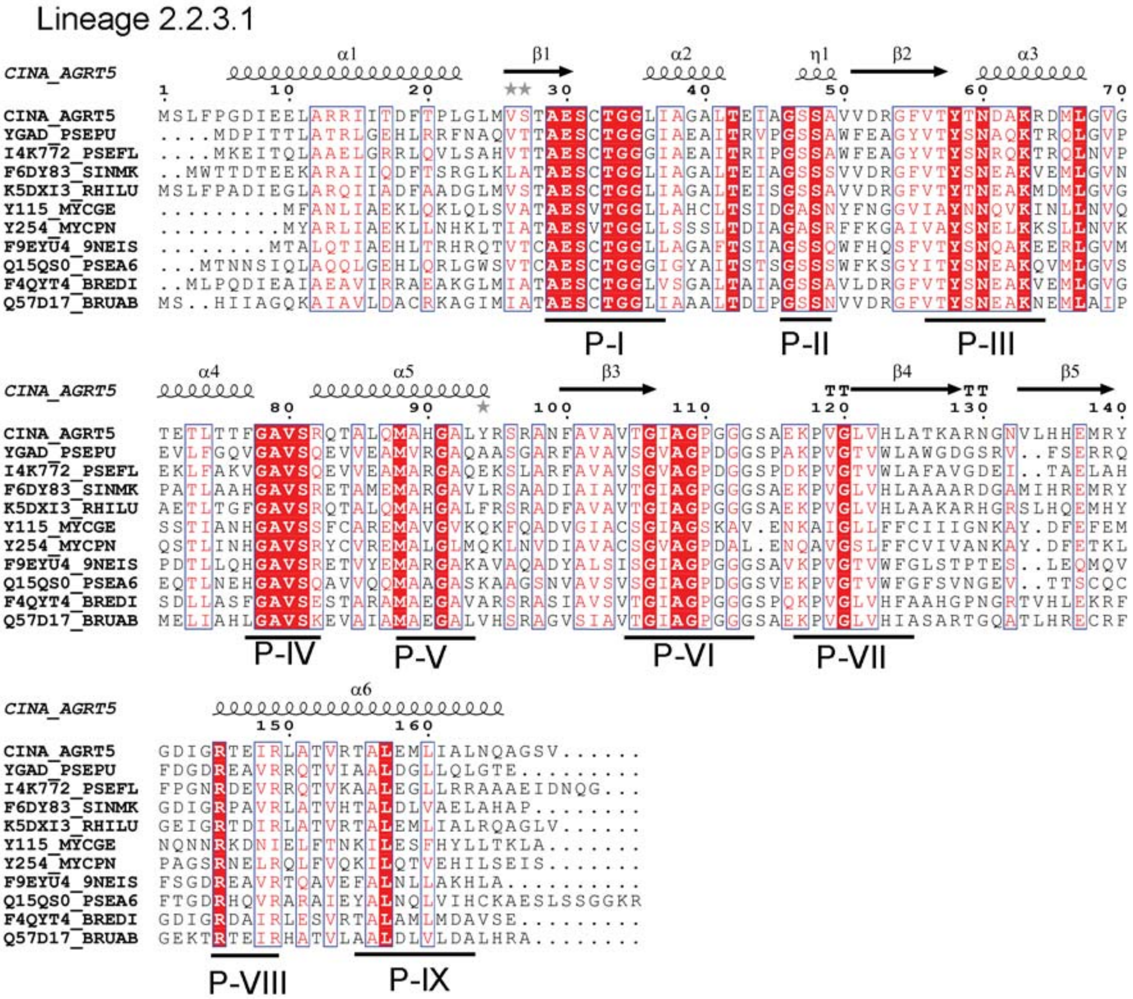
Lineage 2.2.3.1
α1 β1 α2 η1 β2 α3
CINA_AGRT5
1 10 20 30 40 50 60 70
CINA_AGRT5 MSLFPGDIEELARRIITDFTPLGLMVSTAESCTGGLIAGALTEIAGSSAVVDRGFVTYTNDAKRDMLGVG
YGAD_PSEPU ....MDPITTLATRLGEHLRRFNAQVTTAESCTGGIAEAITRVPGSSAWFEAGYVTYSNAQKTRQLGVP
I4K772_PSEFL ....MKEITQLAAELGRRLQVLSAHVTTAESCTGGIAEAITRIPGSSAWFEAGYVTYSNRQKTRQLNVP
F6DY83_SINMK ..MWTTDTEEKARAIIQDFTSRGLKLATAESCTGGLIAGALTEIAGSSSVVDRGFVTYSNEAKVEMLGVN
K5DXI3_RHILU MSLFPADIEGLARQIIADFAADGLMVSTAESCTGGLIAGALTEISGSSAVVDRGFVTYTNEAKMDMLGVG
Y115_MYCGE .........MFANLIAEKLQKLQLSVATAESVTGGLLAHCLTSIDGASNYFNGGVIAYNNQVKINLLNVQ
Y254_MYCPN .........MYARLIAEKLLNHKLTIATAESVTGGLLSSSLTDIAGASRFFKGAIVAYSNELKKSLLNVK
F9EYU4_9NEIS ........MTALQTIAEHLTRHRQTVTCAESCTGGLLAGAFTSIAGSSQWFHQSFVTYSNQAKEERLGVM
Q15QS0_PSEA6 ...MTNNSIQLAQQLGEHLQRLGWSVTCAESCTGGGIGYAITSTSGSSSWFKSGYITYSNEAKQVMLGVS
F4QYT4_BREDI ..MLPQDIEAIAEAVIRRAEAKGLMIATAESCTGGLVSGALTAIAGSSAVLDRGFVTYSNEAKVEMLGVG
Q57D17_BRUAB MS..HIIAGQKAIAVLDACRKAGIMIATAESCTGGLIAAALTDIPGSSNVVDRGFVTYSNEAKNEMLAIP
P-I P-II P-III
α4 α5 β3 TT β4 TT β5
CINA_AGRT5
80 90 100 110 120 130 140
CINA_AGRT5 TETLTTFGAVSRQTALQMAHGALYRSRANFAVAVTGIAGPGGGSAEKPVGLVHLATKARNGNVLHHEMRY
YGAD_PSEPU EVLFGQVGAVSQEVVEAMVRGAQAASGARFAVAVSGVAGPDGGSPAKPVGTVWLAWGDGSRV..FSERRQ
I4K772_PSEFL EKLFAKVGAVSQEVVEAMARGAQEKSLARFAVAVSGVAGPDGGSPDKPVGTVWLAFAVGDEI..TAELAH
F6DY83_SINMK PATLAAHGAVSRETAMEMARGAVLRSAADIAIAVTGIAGPGGGSAEKPVGLVHLAAAARDGAMIHREMRY
K5DXI3_RHILU AETLTGFGAVSRQTALQMAHGALFRSRADFAVAVTGIAGPGGGSAEKPVGLVHLAAKARHGRSLHQEMHY
Y115_MYCGE SSTIANHGAVSSFCAREMAVGVKQKFQADVGIACSGIAGSKAV.ENKAIGLLFFCIIIGNKAY.DFEFEM
Y254_MYCPN QSTLINHGAVSRYCVREMALGLMQKLNVDIAVACSGVAGPDAL.ENQAVGSLFFCVIVANKAY.DFETKL
F9EYU4_9NEIS PDTLLQHGAVSRETVYEMARGAKAVAQADYALSISGIAGPGGGSAVKPVGTVWFGLSTPTES..LEQMQV
Q15QS0_PSEA6 EQTLNEHGAVSQAVVQQMAAGASKAAGSNVAVSVSGIAGPDGGSVEKPVGTVWFGFSVNGEV..TTSCQC
F4QYT4_BREDI SDLLASFGAVSESTARAMAEGAVARSRASIAVSVTGIAGPGGGSPQKPVGLVHFAAHGPNGRTVHLEKRF
Q57D17_BRUAB MELIAHLGAVSKEVAIAMAEGALVHSRAGVSIAVTGIAGPGGGSAEKPVGLVHIASARTGQATLHRECRF
P-IV P-V P-VI P-VII
α6
CINA_AGRT5
150 160
CINA_AGRT5 GDIGRTEIRLATVRTALEMLIALNQAGSV......
YGAD_PSEPU FDGDREAVRRQTVIAALDGLLQLGTE.........
I4K772_PSEFL FPGNRDEVRRQTVKAALEGLLRRAAAEIDNQG...
F6DY83_SINMK GDIGRPAVRLATVHTALDLVAELAHAP........
K5DXI3_RHILU GEIGRTDIRLATVRTALEMLIALRQAGLV......
Y115_MYCGE NQNNRKDNIELFTNKILESFHYLLTKLA.......
Y254_MYCPN PAGSRNELRQLFVQKILQTVEHILSEIS.......
F9EYU4_9NEIS FSGDREAVRTQAVEFALNLLAKHLA..........
Q15QS0_PSEA6 FTGDRHQVRARAIEYALNQLVIHCKAESLSSGGKR
F4QYT4_BREDI GDIGRDAIRLESVRTALAMLMDAVSE.........
Q57D17_BRUAB GEKTRTEIRHATVLAALDLVLDALHRA........
P-VIII P-IX

Lineage 2.2.3.2

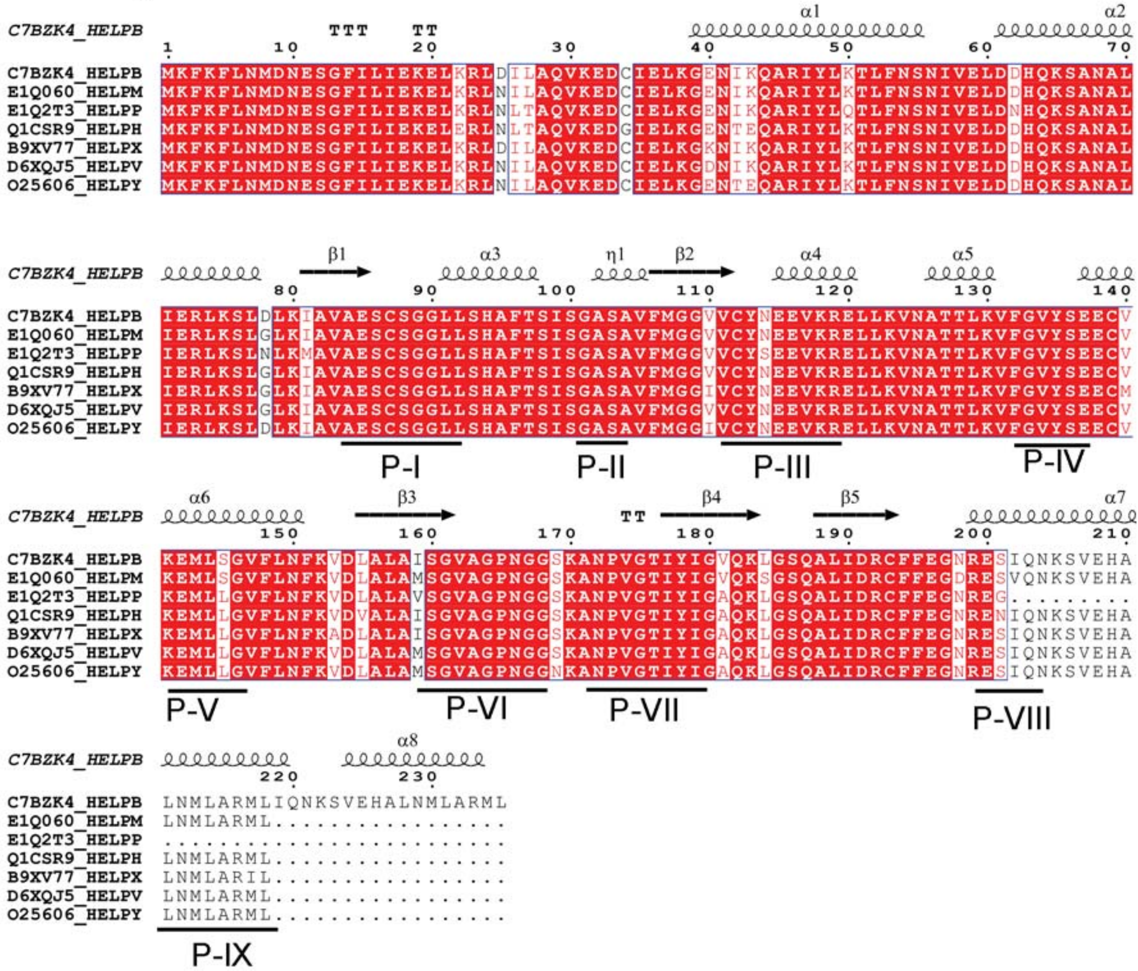

Lineage 2.2.3.3

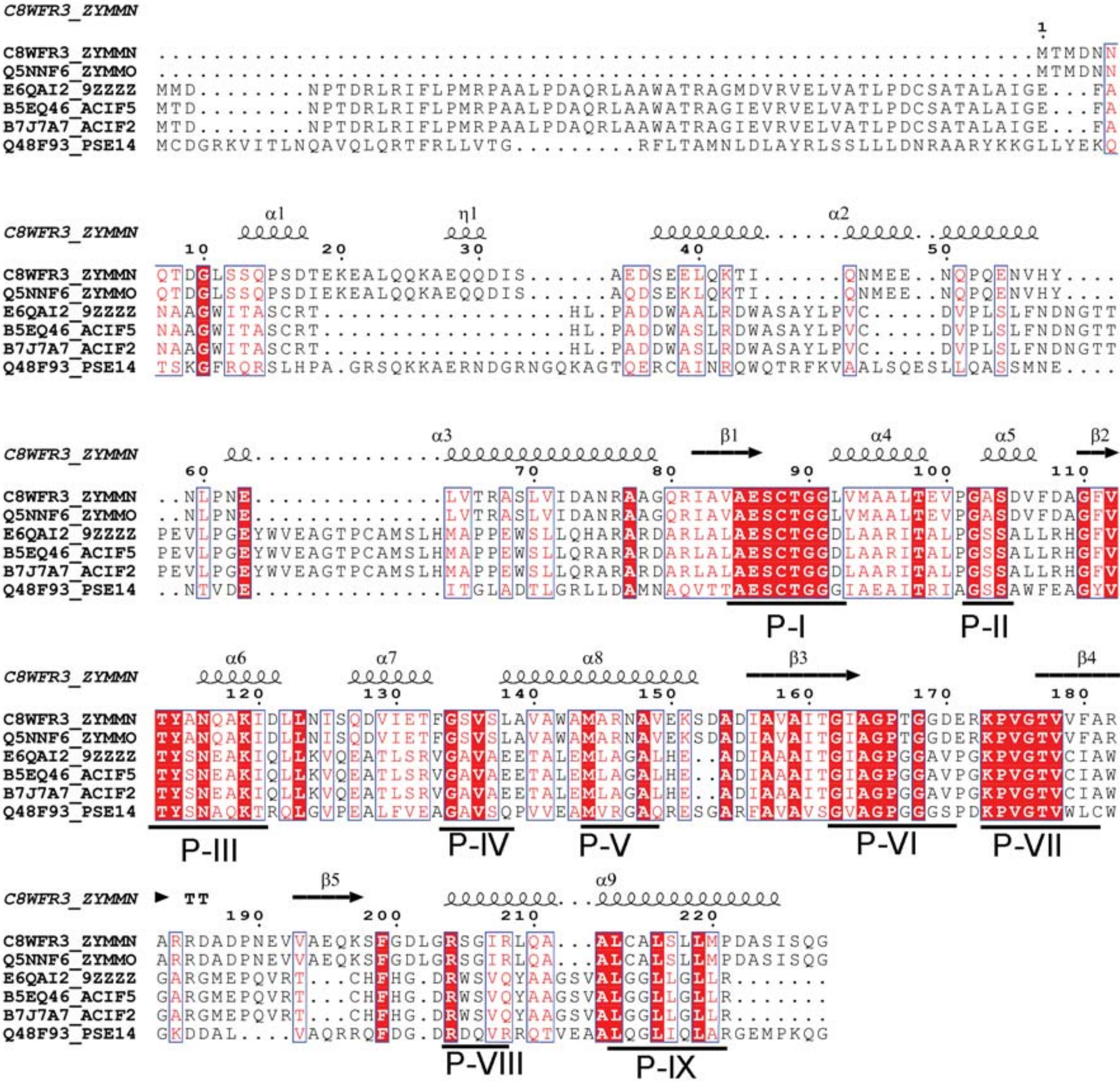

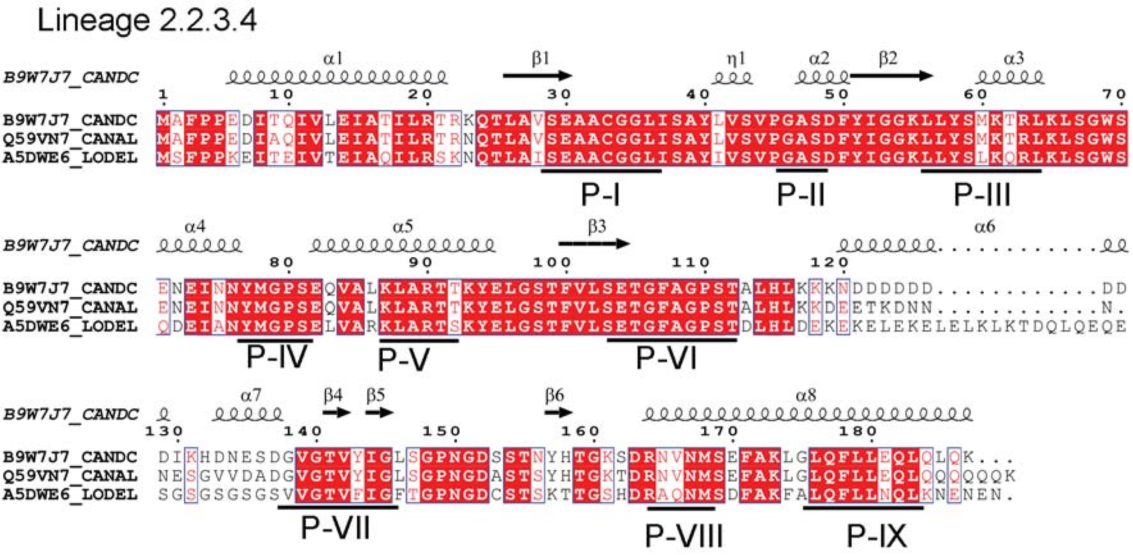

Lineage 2.2.3.4
B9W7J7_CANDC
Q59VN7_CANAL
A5DWE6_LODEL
P-I
P-II
P-III
P-IV
P-V
P-VI
P-VII
P-VIII
P-IX
