# Supplementary material for: New Insights into the Phylogeny and Molecular Classification of Nicotinamide Mononucleotide Deamidases
Source: PLoS One. 2013 Dec 5;8(12):e82705. doi: 10.1371/journal.pone.0082705 (PMC3855486; doi:10.1371/journal.pone.0082705)
Supplement: Figure S8 — WEBLOGO outputs obtained from the above alignment of the sequences from the different lineages. Conserved blocks are marked under the corresponding sequences for each lineage. (PDF) [file pone.0082705.s008.pdf]

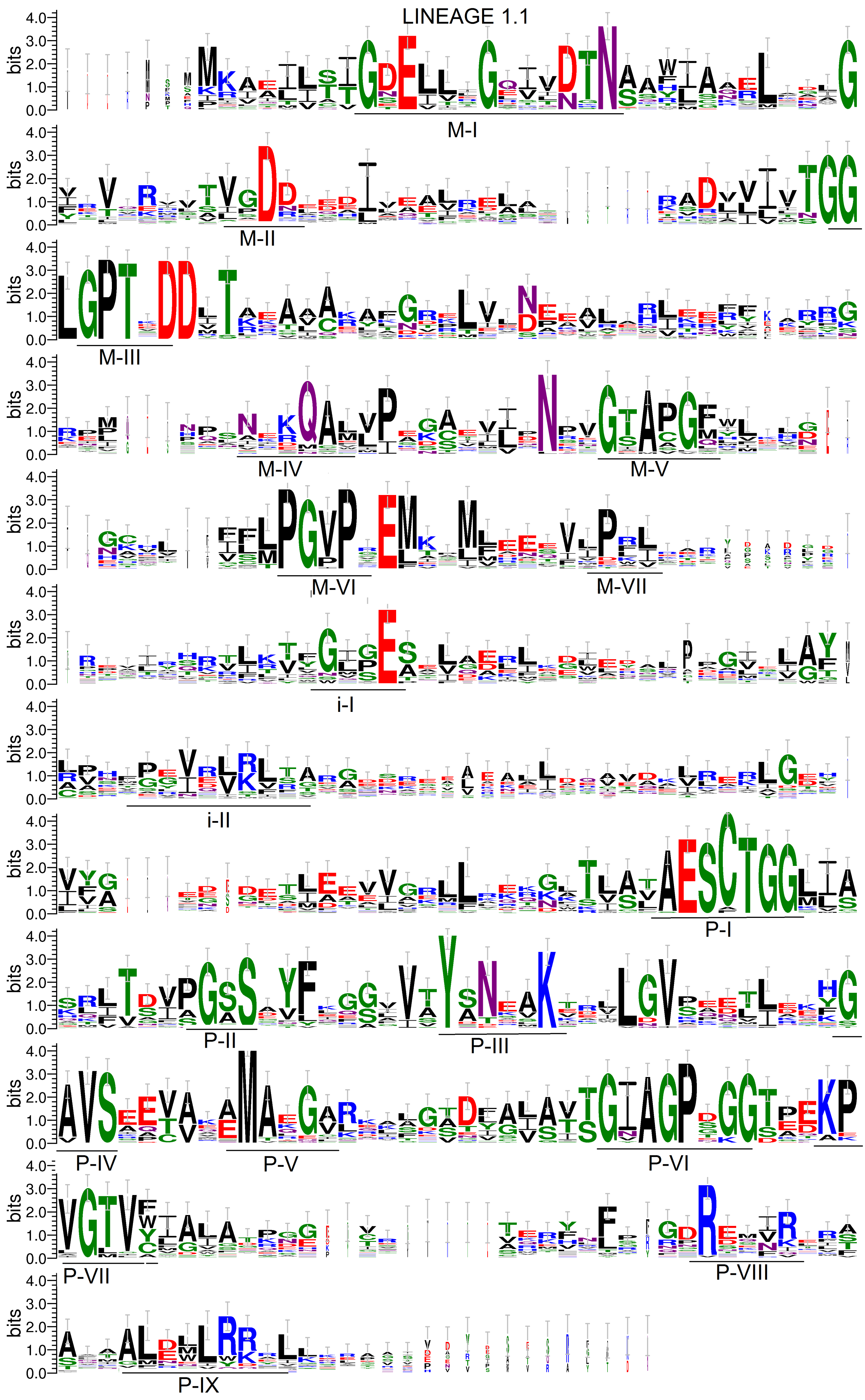
LINEAGE 1.1
bits
M-I
M-II
M-III
M-IV
M-V
M-VI
M-VII
i-I
i-II
P-I
P-II
P-III
P-IV
P-V
P-VI
P-VII
P-VIII
P-IX

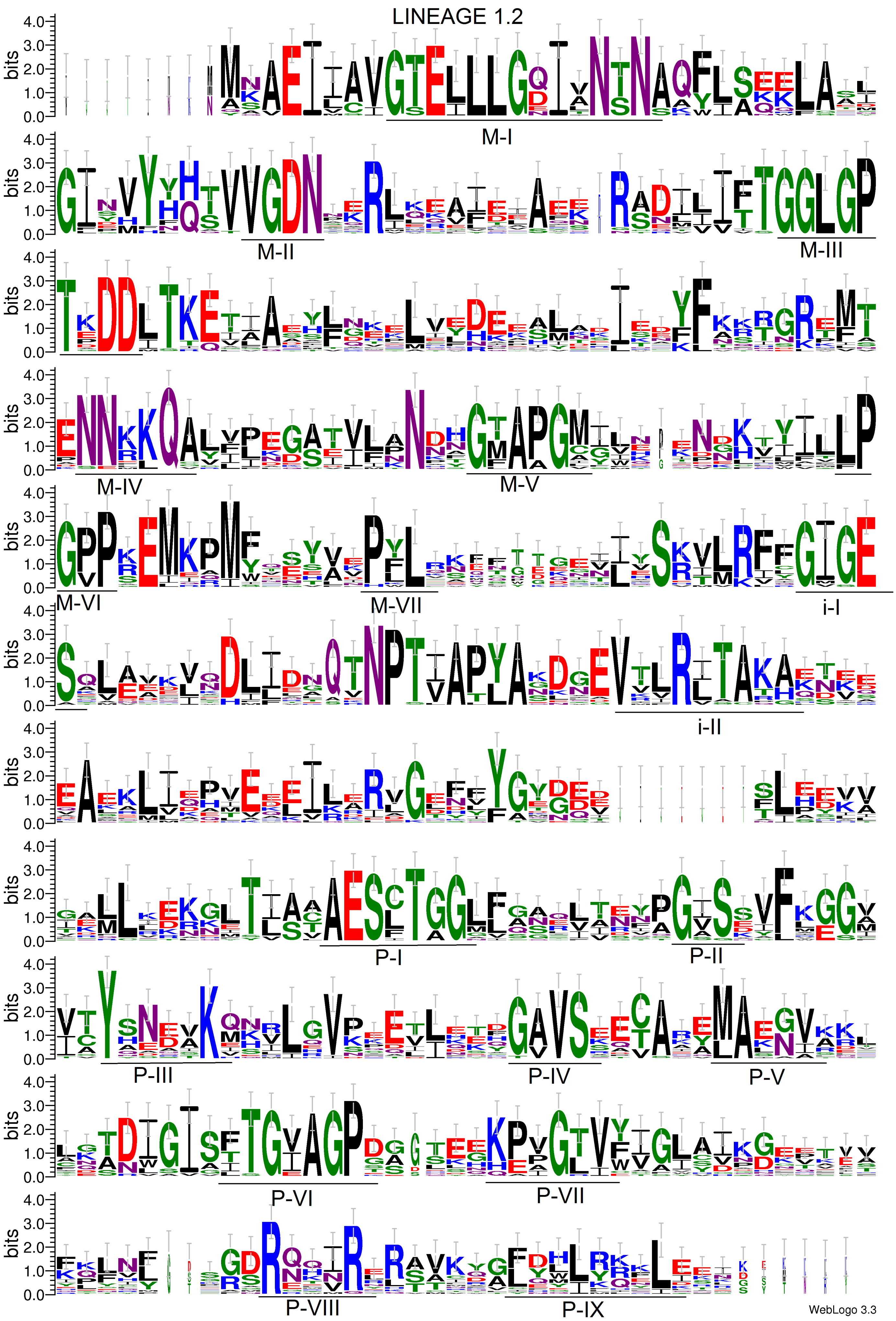

LINEAGE 1.2
bits
M-I
M-II
M-III
M-IV
M-V
M-VI
M-VII
i-I
i-II
P-I
P-II
P-III
P-IV
P-V
P-VI
P-VII
P-VIII
P-IX
WebLogo 3.3

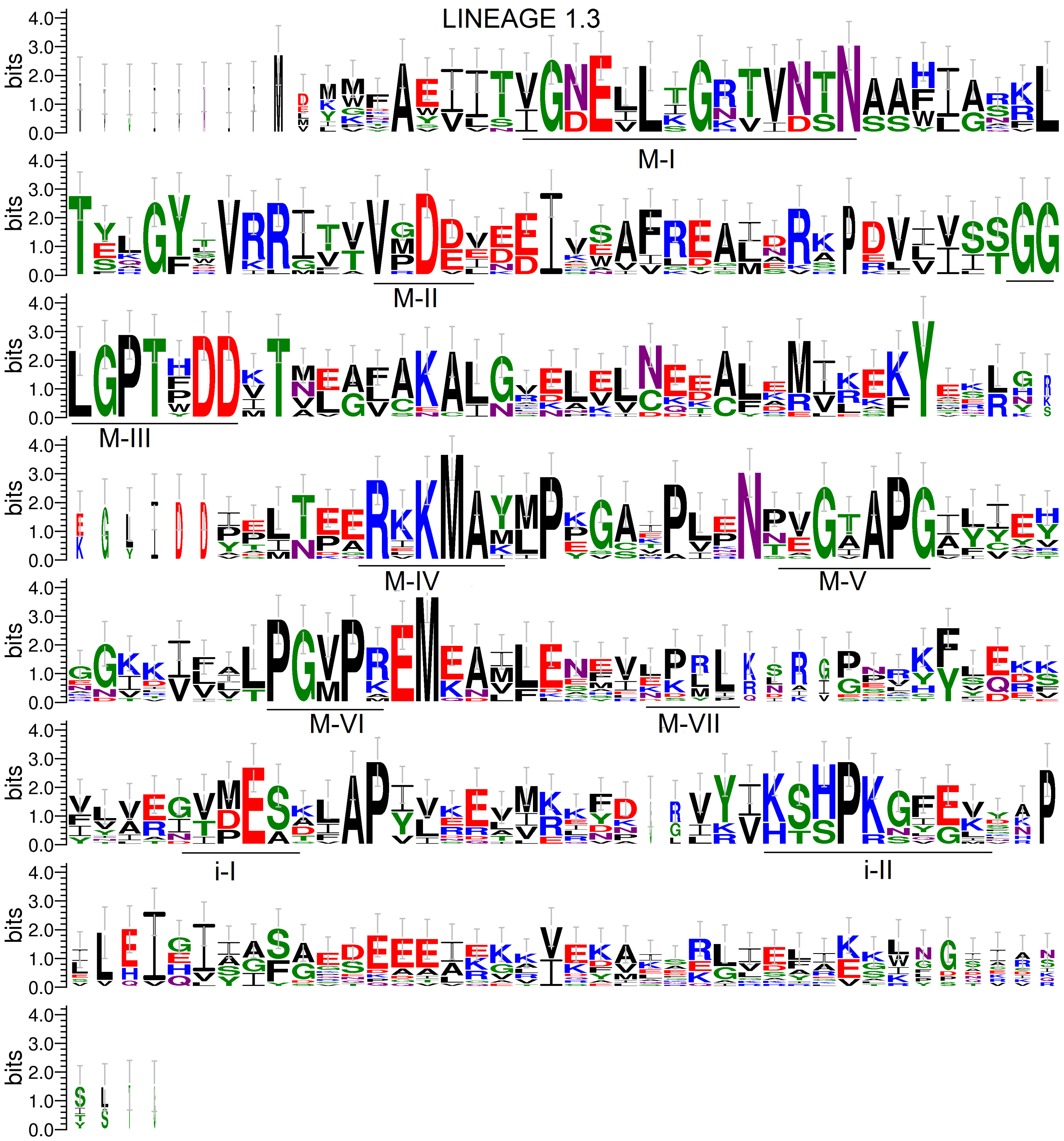
LINEAGE 1.3
bits
4.0
3.0
2.0
1.0
0.0
M-I
M-II
M-III
M-IV
M-V
M-VI
M-VII
i-I
i-II

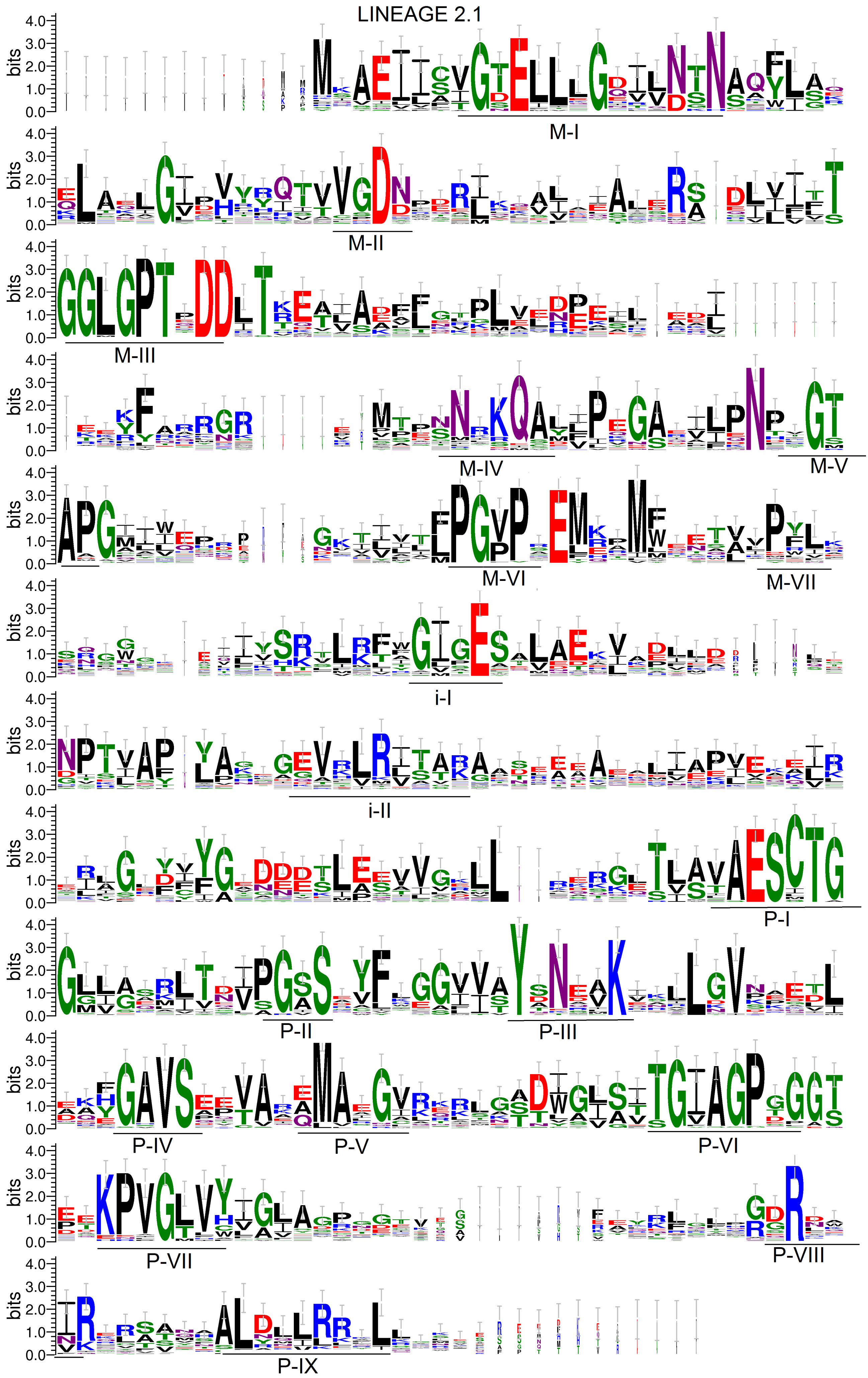

LINEAGE 2.1
bits
M-I
M-II
M-III
M-IV
M-V
M-VI
M-VII
i-I
i-II
P-I
P-II
P-III
P-IV
P-V
P-VI
P-VII
P-VIII
P-IX

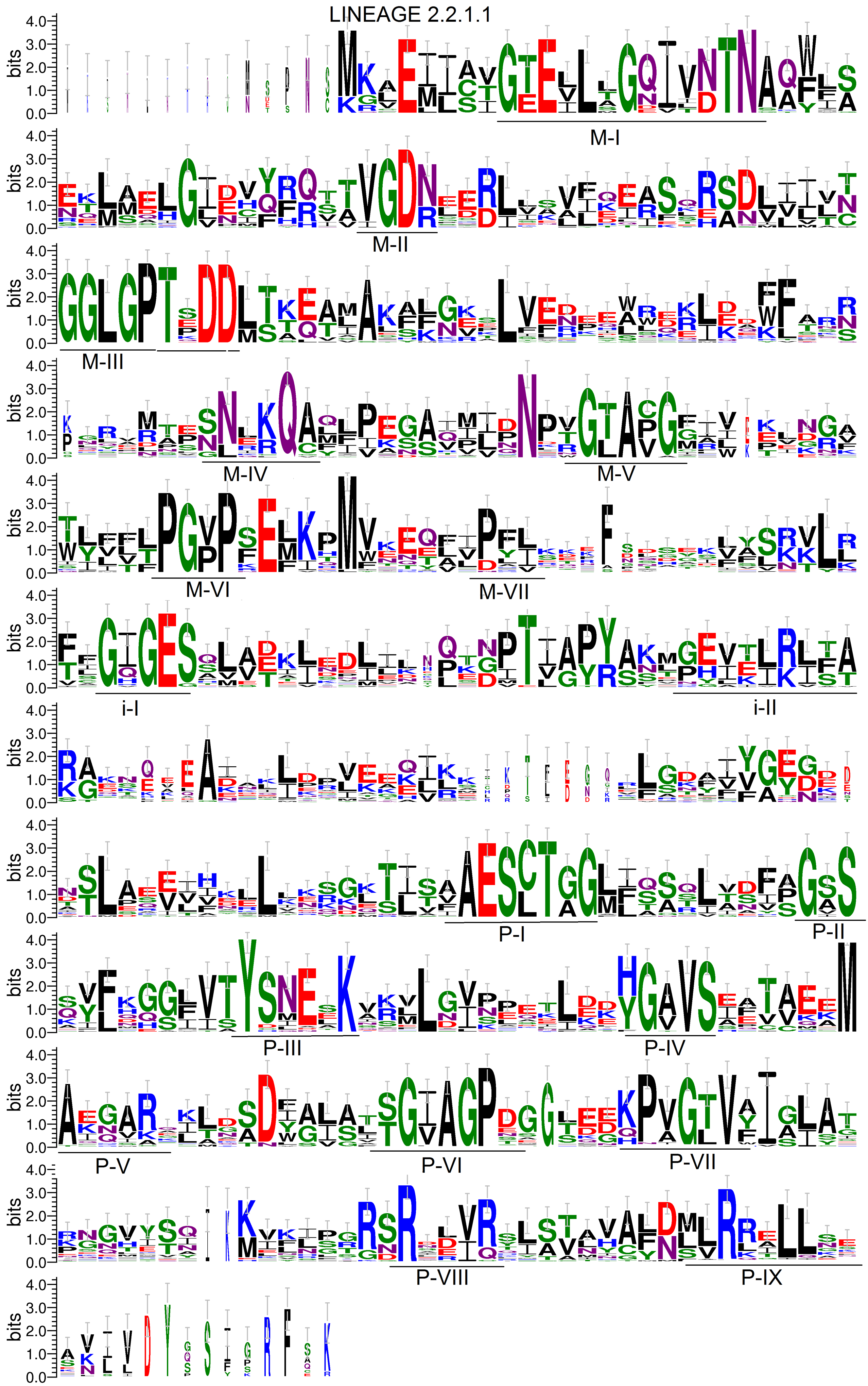
LINEAGE 2.2.1.1
bits
4.0
3.0
2.0
1.0
0.0
M-I
M-II
M-III
M-IV
M-V
M-VI
M-VII
i-I
i-II
P-I
P-II
P-III
P-IV
P-V
P-VI
P-VII
P-VIII
P-IX

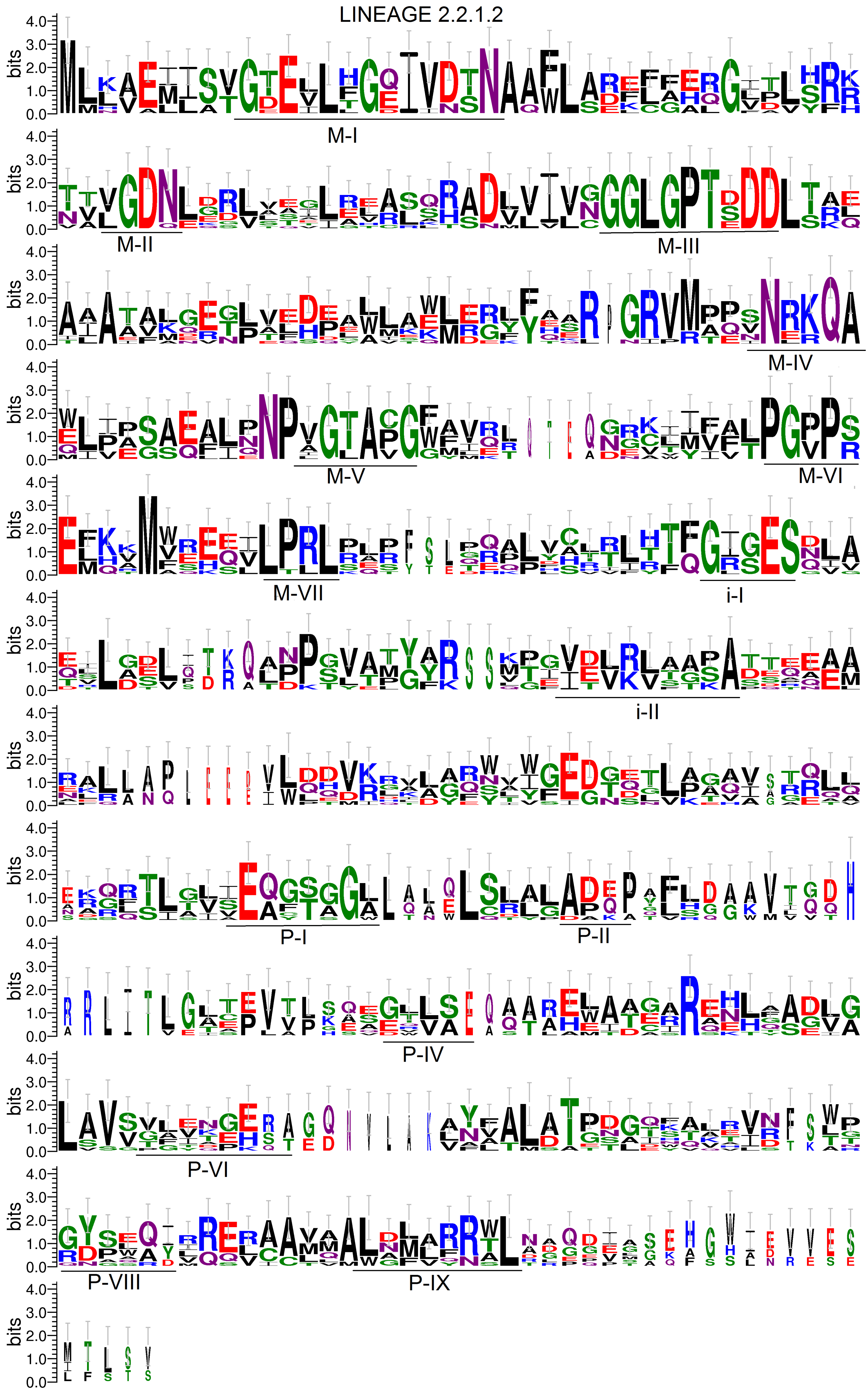
LINEAGE 2.2.1.2
bits
4.0
3.0
2.0
1.0
0.0
M-I
M-II
M-III
M-IV
M-V
M-VI
M-VII
i-I
i-II
P-I
P-II
P-IV
P-VI
P-VIII
P-IX

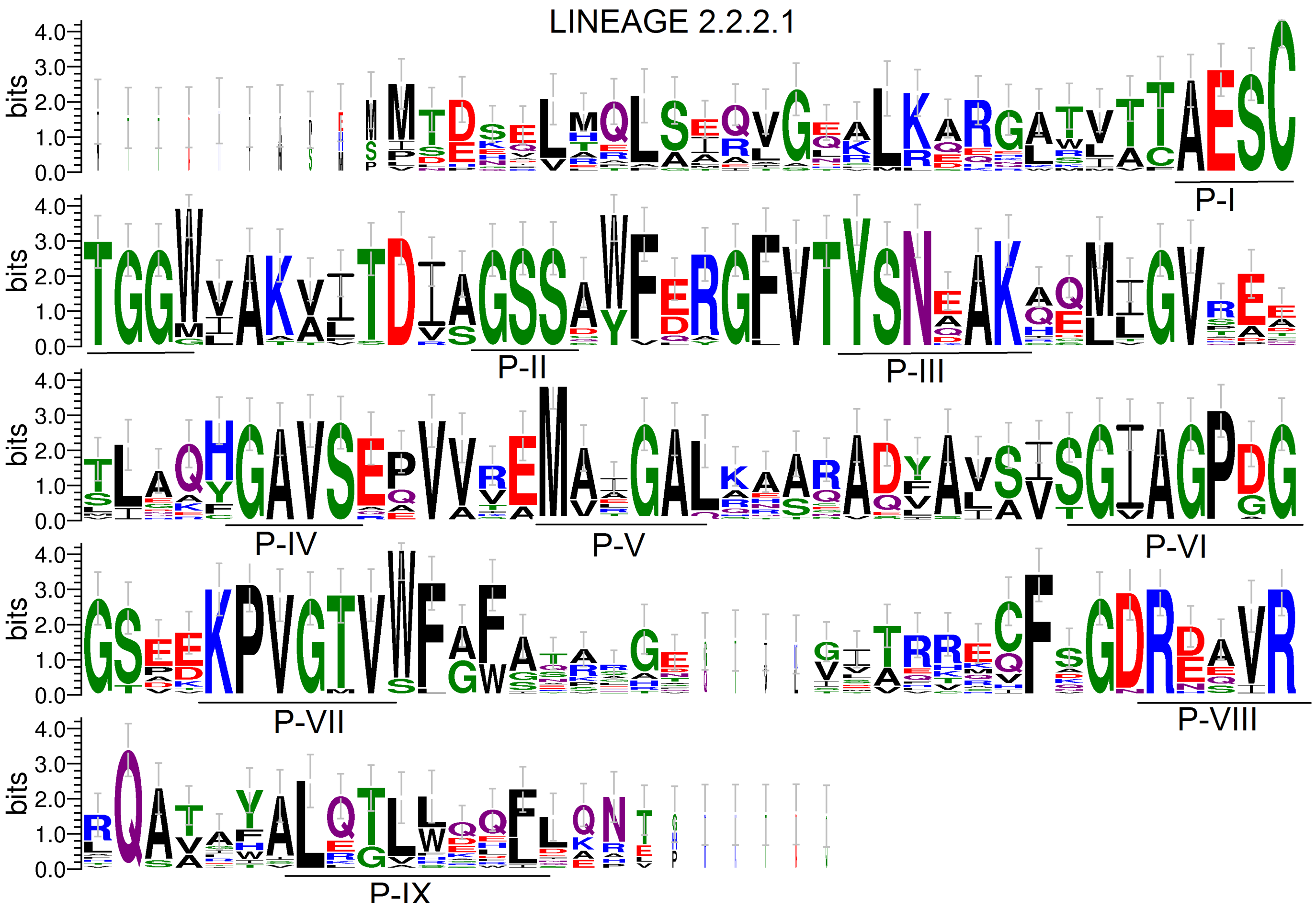
LINEAGE 2.2.2.1
bits
4.0
3.0
2.0
1.0
0.0
P-I
P-II
P-III
P-IV
P-V
P-VI
P-VII
P-VIII
P-IX

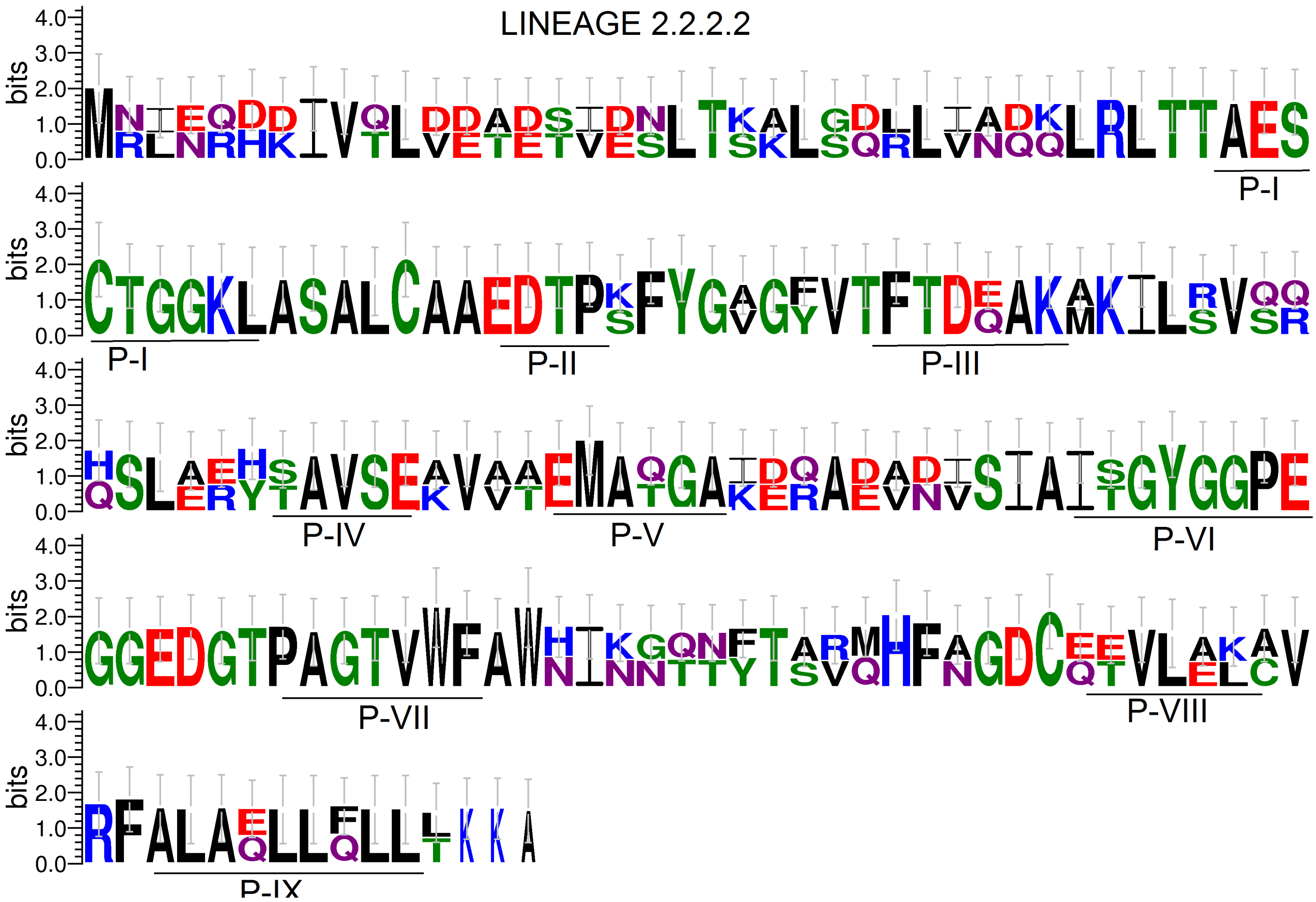
LINEAGE 2.2.2.2
bits
4.0
3.0
2.0
1.0
0.0
P-I
P-II
P-III
P-IV
P-V
P-VI
P-VII
P-VIII
P-IX

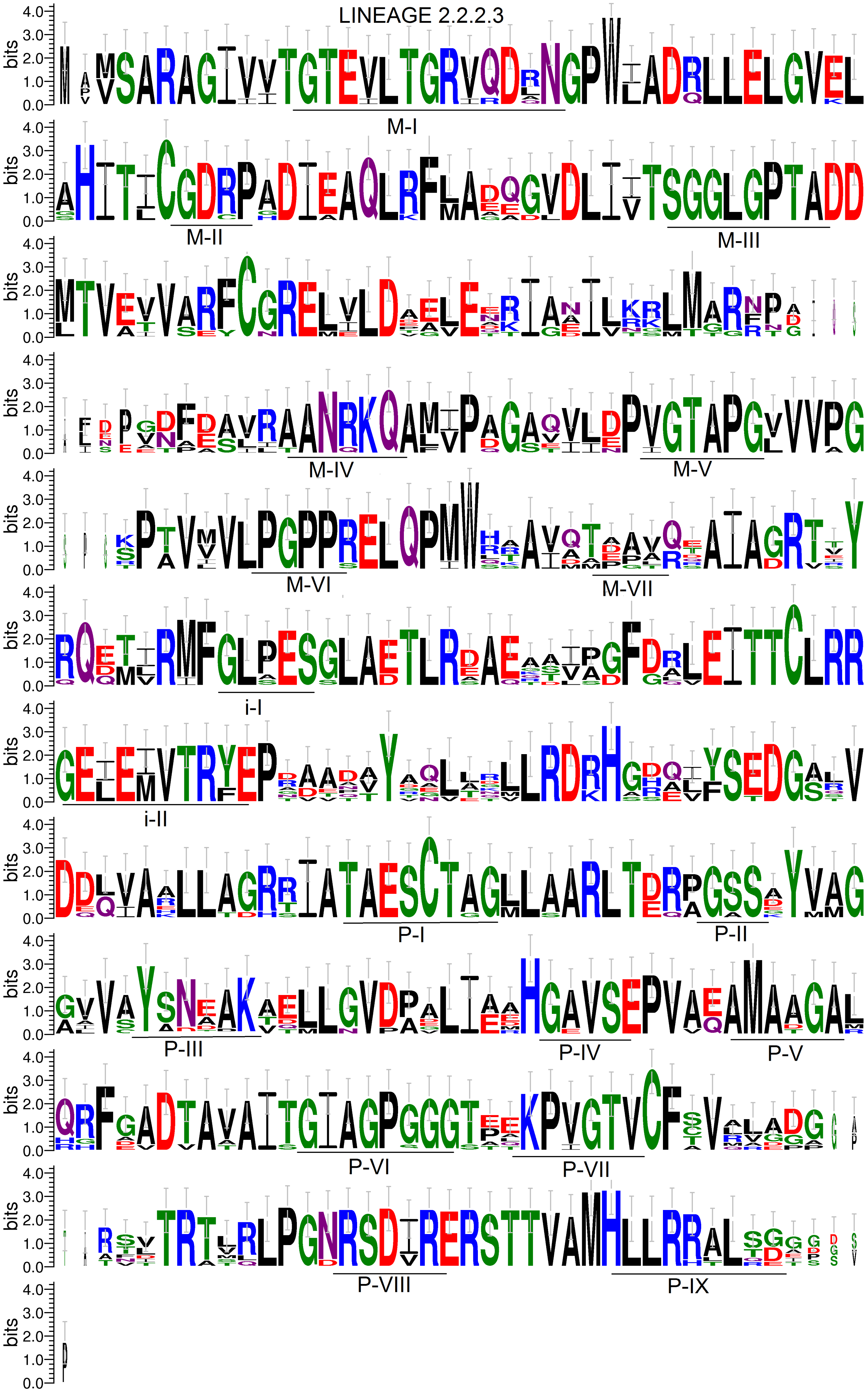
LINEAGE 2.2.2.3
bits
M-I
M-II
M-III
M-IV
M-V
M-VI
M-VII
i-I
i-II
P-I
P-II
P-III
P-IV
P-V
P-VI
P-VII
P-VIII
P-IX

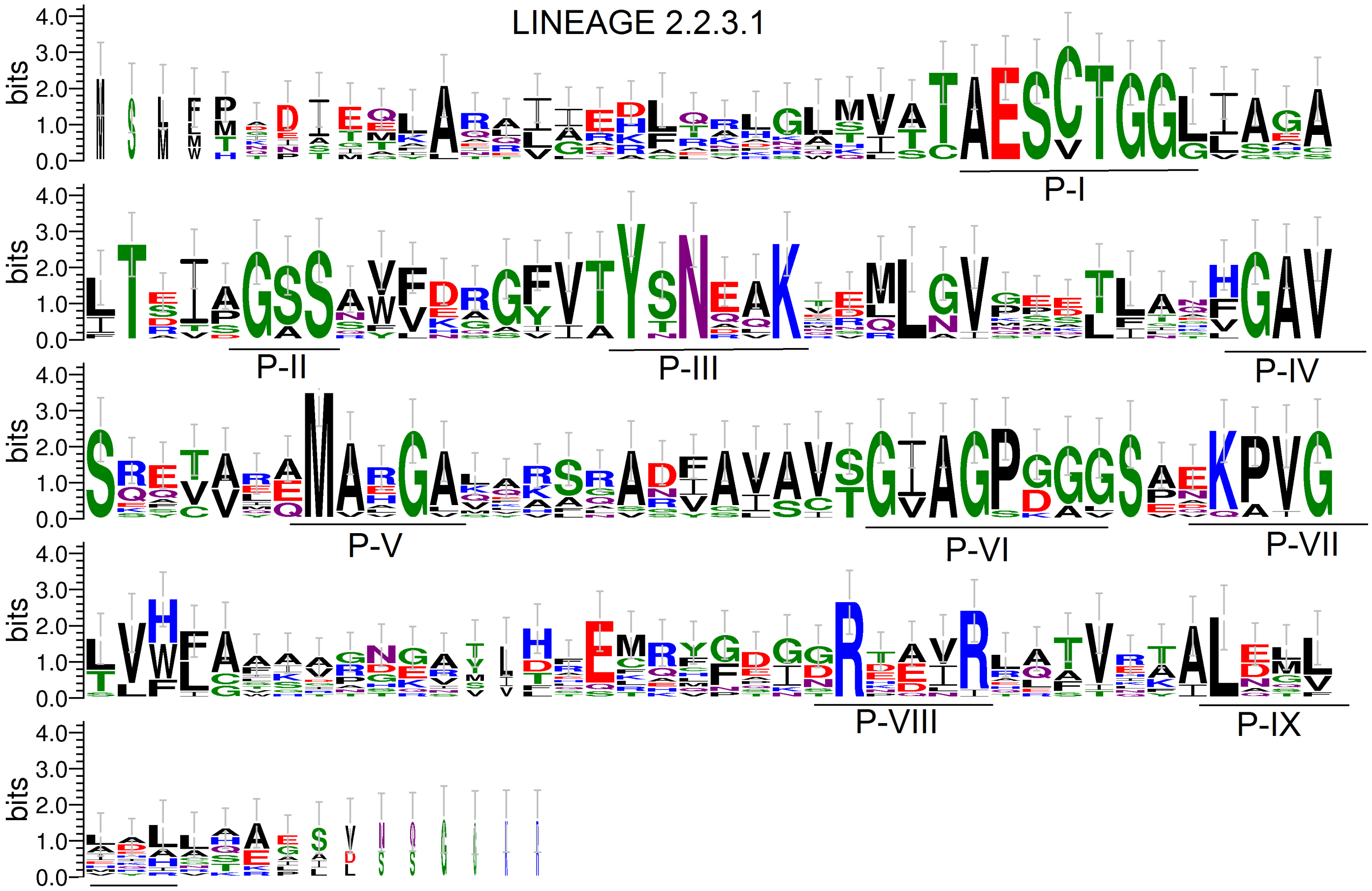
LINEAGE 2.2.3.1
bits
0.0
1.0
2.0
3.0
4.0
P-I
P-II
P-III
P-IV
P-V
P-VI
P-VII
P-VIII
P-IX

LINEAGE 2.2.3.2

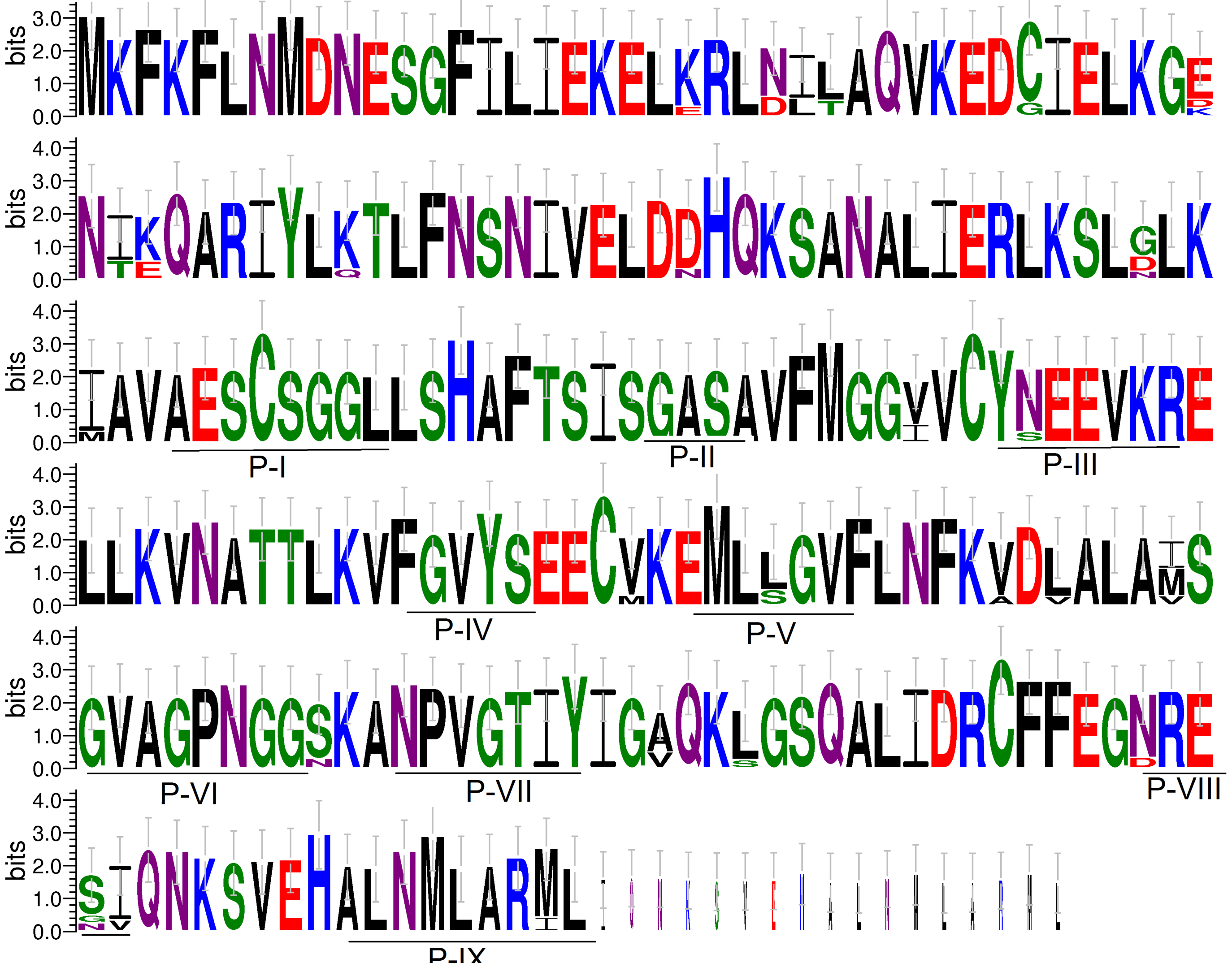

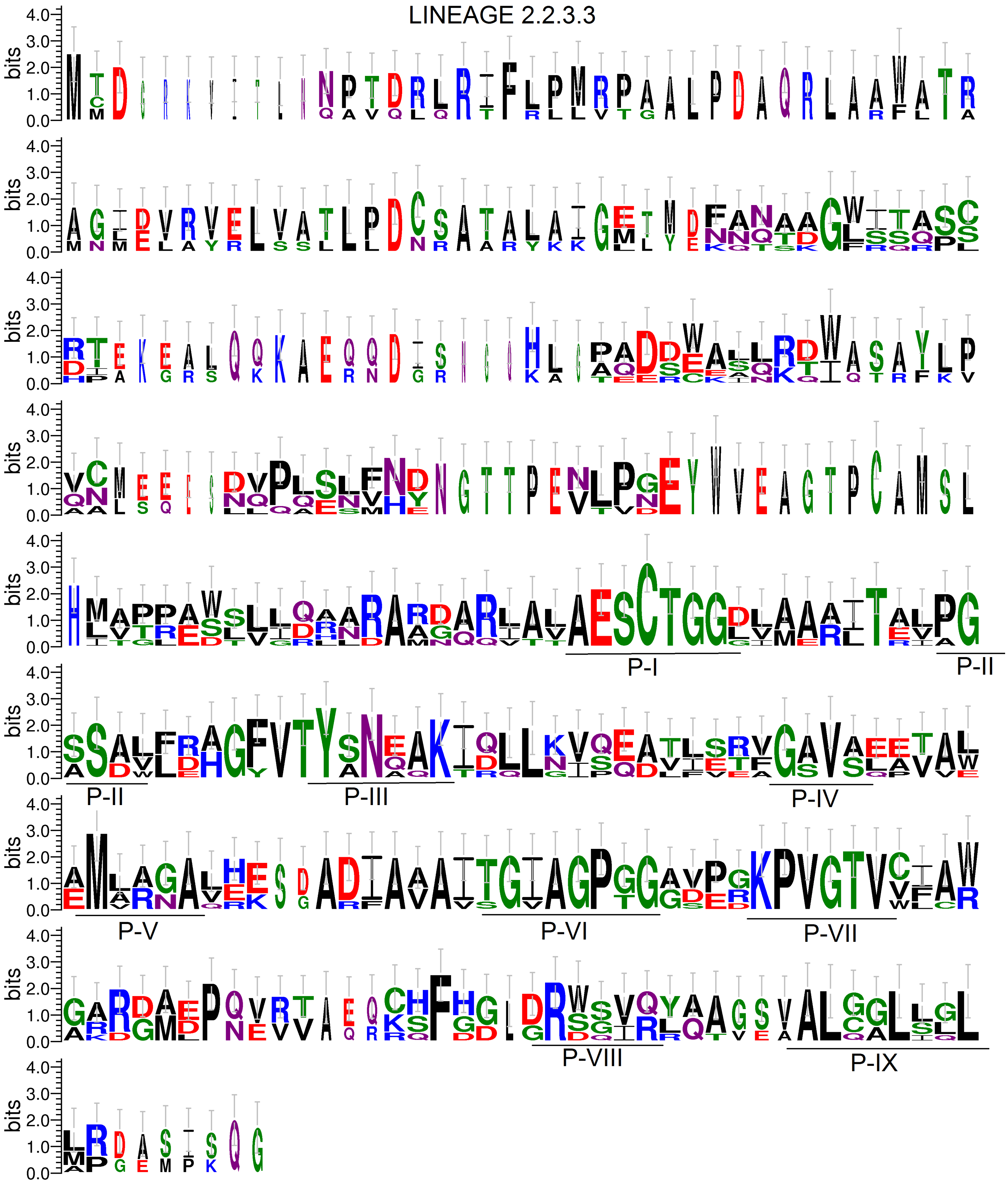
LINEAGE 2.2.3.3
bits
4.0
3.0
2.0
1.0
0.0
P-I
P-II
P-III
P-IV
P-V
P-VI
P-VII
P-VIII
P-IX

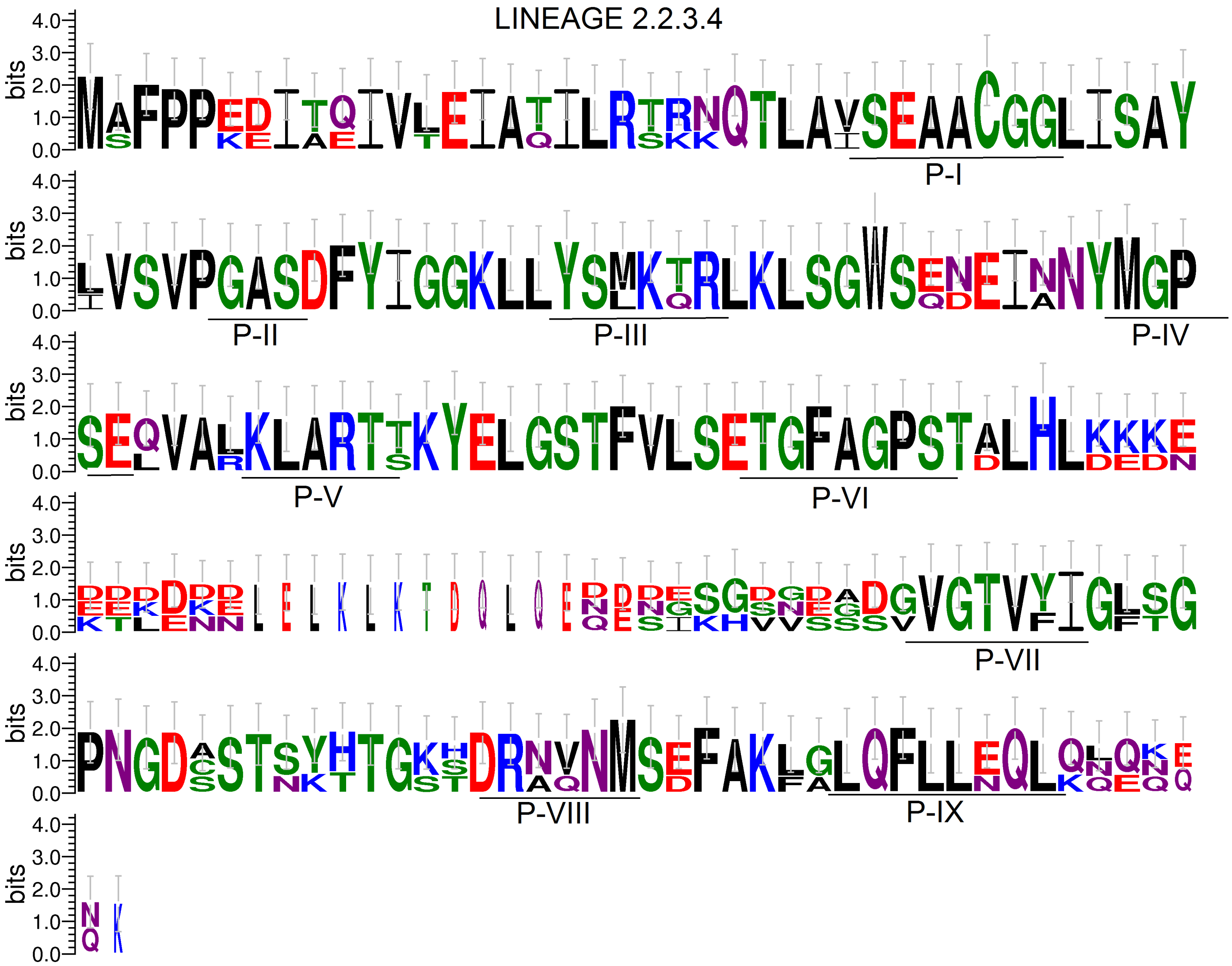
LINEAGE 2.2.3.4
bits
0.0
1.0
2.0
3.0
4.0
P-I
P-II
P-III
P-IV
P-V
P-VI
P-VII
P-VIII
P-IX
