# Supplementary material for: New Insights into the Phylogeny and Molecular Classification of Nicotinamide Mononucleotide Deamidases
Source: PLoS One. 2013 Dec 5;8(12):e82705. doi: 10.1371/journal.pone.0082705 (PMC3855486; doi:10.1371/journal.pone.0082705)
Supplement: Table S1 — NMN deamidases used in the phylogenetic analysis. (PDF) [file pone.0082705.s009.pdf]

C1F6H5	**CINAL ACIC5**	Acidobacterium capsulatum (strain ATCC 51196)	Acidobacteria
Q1IPQ9	**CINAL KORVE**	Koribacter versatilis (strain Ellin345)	Acidobacteria
Q028J4	**CINAL SOLUE**	Solibacter usitatus (strain Ellin6076)	Acidobacteria
A0LV09	**CINAL ACIC1**	Acidothermus cellulolyticus (strain ATCC 43068)	Actinobacteria
Q2J752	**CINAL FRASC**	Frankia sp. (strain CcI3)	Actinobacteria
C1API1	**CINAL MYCBT**	Mycobacterium bovis (strain BCG)	Actinobacteria
A4TAK5	**CINAL MYCGI**	Mycobacterium gilvum (strain PYR-GCK)	Actinobacteria
Q9X7D6	**CINAL MYCLE**	Mycobacterium leprae (strain TN)	Actinobacteria
B2HDA1	**CINAL MYCMM**	Mycobacterium marinum (strain ATCC BAA-535)	Actinobacteria
Q73ZH8	**CINAL MYCPA**	Mycobacterium paratuberculosis (strain ATCC BAA-968)	Actinobacteria
A0QY28	**CINAL MYCS2**	Mycobacterium smegmatis (strain ATCC 700084)	Actinobacteria
A3Q040	**CINAL MYCSJ**	Mycobacterium sp. (strain JLS)	Actinobacteria
A1T9F4	**CINAL MYCVP**	Mycobacterium vanbaalenii (strain DSM 7251)	Actinobacteria
Q1AT03	**CINAL RUBXD**	Rubrobacter xylanophilus (strain DSM 9941)	Actinobacteria
D9UE64	**D9UE64 9ACTO**	Streptomyces sp. SPB78	Actinobacteria
B2V6Y9	**CINAL SULSY**	Sulfurihydrogenibium sp. (strain YO3AOP1)	Aquificae
Q9YAF9	**Y1980 AERPE**	Aeropyrum pernix (strain ATCC 700893)	Archaea
A8ME30	**Y1209 CALMQ**	Caldivirga maquilingensis (strain ATCC 700844)	Archaea
Q8ZYA5	**Y875 PYRAE**	Pyrobaculum aerophilum (strain ATCC 51768)	Archaea
A4WH44	**Y096 PYRAR**	Pyrobaculum arsenaticum (strain DSM 13514)	Archaea
A3MS85	**Y062 PYRCJ**	Pyrobaculum calidifontis (strain JCM 11548)	Archaea
Q9UYB5	**Y1593 PYRAB**	Pyrococcus abyssi (strain GE5)	Archaea
Q8U3J0	**Y476 PYRFU**	Pyrococcus furiosus (strain ATCC 43587)	Archaea
O58186	**Y439 PYRHO**	Pyrococcus horikoshii (strain ATCC 700860	Archaea
Q5JJB4	**Y1472 PYRKO**	Pyrococcus kodakaraensis (strain ATCC BAA-918)	Archaea
C4KJ66	**Y2028 SULIK**	Sulfolobus islandicus (strain M.16.4)	Archaea
P95958	**Y103 SULSO**	Sulfolobus solfataricus (strain ATCC 35092)	Archaea
Q971E0	**Y1413 SULTO**	Sulfolobus tokodaii (strain DSM 16993)	Archaea
Q9HKV6	**Y487 THEAC**	Thermoplasma acidophilum (strain ATCC 25905)	Archaea
Q97B73	**Y584 THEVO**	Thermoplasma volcanium (strain ATCC 51530)	Archaea
B3ER86	**CINAL AMOA5**	Amoebophilus asiaticus (strain 5a2)	Bacteroidetes
Q5LCF4	**CINAL BACFN**	Bacteroides fragilis (strain ATCC 25285)	Bacteroidetes
Q11S34	**CINAL CYTH3**	Cytophaga hutchinsonii (strain ATCC 33406)	Bacteroidetes
A5F9Y3	**CINAL FLAJ1**	Flavobacterium johnsoniae (strain ATCC 17061)	Bacteroidetes
A6GZI7	**CINAL FLAPJ**	Flavobacterium psychrophilum (strain JIP02)	Bacteroidetes
A0M3L5	**CINAL GRAFK**	Gramella forsetii (strain KT0803)	Bacteroidetes
Q2S304	**CINAL SALRD**	Salinibacter ruber (strain DSM 13855)	Bacteroidetes
B3QL60	**CINAL CHLP8**	Chlorobaculum parvum (strain NCIB 8327)	Chlorobi
CQ3AUA4	**CINAL CHLCH**	Chlorobium chlorochromatii (strain CaD3)	Chlorobi
B3EHJ0	**CINAL CHLL2**	Chlorobium limicola (strain DSM 245)	Chlorobi
B3EL42	**CINAL CHLPB**	Chlorobium phaeobacteroides (strain BS1)	Chlorobi
A1BJF2	**CINAL CHLPD**	Chlorobium phaeobacteroides (strain DSM 266)	Chlorobi
Q8KAX2	**CINAL CHLTE**	Chlorobium tepidum (strain ATCC 49652)	chlorobi
B3QTC8	**CINAL CHLT3**	Chloroherpeton thalassium (strain ATCC 35110)	Chlorobi
Q3B1F7	**CINAL PELLD**	Pelodictyon luteolum (strain DSM 273)	Chlorobi
A4SGM6	**CINAL PROVI**	Prosthecochloris vibrioformis (strain DSM 265)	Chlorobi
A9WJE6	**A9WJE6 CHLAA**	Chloroflexus aurantiacus (strain ATCC 29366)	Chloroflexi
B9L1M7	**CINAL THERP**	Thermomicrobium roseum (strain ATCC 27502)	Chloroflexi
B0CEJ2	**CINAL ACAM1**	Acaryochloris marina (strain MBIC 11017)	Cyanobacteria
Q3MBD6	**CINAL ANAVT**	Anabaena variabilis (strain ATCC 29413)	Cyanobacteria

B1WSM0	**CINAL CYAA5**	Cyanothece sp. (strain ATCC 51142)	Cyanobacteria
B7KFE8	**CINAL CYAP7**	Cyanothece sp. (strain PCC 7424)	Cyanobacteria
B8HR57	**CINAL CYAP4**	Cyanothece sp. (strain PCC 7425)	Cyanobacteria
B7JYM6	**CINAL CYAP8**	Cyanothece sp. (strain PCC 8801)	Cyanobacteria
Q7NHU4	**CINAL GLOVI**	Gloeobacter violaceus (strain PCC 7421)	Cyanobacteria
B0JVG7	**CINAL MICAN**	Microcystis aeruginosa (strain NIES-843)	Cyanobacteria
B2J2A3	**CINAL NOSP7**	Nostoc punctiforme (strain ATCC 29133)	Cyanobacteria
Q8YMW6	**CINAL NOSS1**	Nostoc sp. (strain PCC 7120)	Cyanobacteria
CA2BP56	**CINAL PROMS**	Prochlorococcus marinus (strain AS9601)	Cyanobacteria
A8G2R7	**CINAL PROM2**	Prochlorococcus marinus (strain MIT 9215)	Cyanobacteria
A3PAX8	**CINAL PROM0**	Prochlorococcus marinus (strain MIT 9301)	Cyanobacteria
Q31CS5	**CINAL PROM9**	Prochlorococcus marinus (strain MIT 9312)	Cyanobacteria
Q7TUN0	**CINAL PROMM**	Prochlorococcus marinus (strain MIT 9313)	Cyanobacteria
CA2BUN8	**CINAL PROM5**	Prochlorococcus marinus (strain MIT 9515)	Cyanobacteria
A2C089	**CINAL PROM1**	Prochlorococcus marinus (strain NATL1A)	Cyanobacteria
Q7VDS9	**CINAL PROMA**	Prochlorococcus marinus (strain SARG)	Cyanobacteria
Q7TUG2	**CINAL PROMP**	Prochlorococcus marinus subsp. pastoris (strain CCMP1986)	Cyanobacteria
Q5N2P7	**CINAL SYNP6**	Synechococcus sp. (strain ATCC 27144)	Cyanobacteria
B1XKM7	**CINAL SYNP2**	Synechococcus sp. (strain ATCC 27264)	Cyanobacteria
Q3AN01	**CINAL SYNSC**	Synechococcus sp. (strain CC9605)	Cyanobacteria
Q3AW20	**CINAL SYNS9**	Synechococcus sp. (strain CC9902)	Cyanobacteria
A5GWH4	**CINAL SYNR3**	Synechococcus sp. (strain RCC307)	Cyanobacteria
A5GIG6	**CINAL SYNPW**	Synechococcus sp. (strain WH7803)	Cyanobacteria
Q7U9J5	**CINAL SYNPX**	Synechococcus sp. (strain WH8102)	Cyanobacteria
Q55760	**CINAL SYNY3**	Synechocystis sp. (strain PCC 6803)	Cyanobacteria
Q8DH31	**CINAL THEEB**	Thermosynechococcus elongatus (strain BP-1)	Cyanobacteria
Q111G9	**CINAL TRIEI**	Trichodesmium erythraeum (strain IMS101)	Cyanobacteria
C1CXY3	**CINAL DEIDV**	Deinococcus deserti (strain VCD115)	Deinococcus-Thermus
Q1IWF4	**CINAL DEIGD**	Deinococcus geothermalis (strain DSM 11300)	Deinococcus-Thermus
CINAL	**CINAL DEIRA**	Deinococcus radiodurans (strain ATCC 13939)	Deinococcus-Thermus
O32508	**CINAL DEIRA2**	Deinococcus radiodurans (strain ATCC 13939)	Deinococcus-Thermus
B5YB52	**CINAL DICT6**	Dictyoglomus thermophilum (strain ATCC 35947)	Dictyoglomi
B8DYM0	**CINAL DICTD**	Dictyoglomus turgidum (strain Z-1310)	Dictyoglomi
B1GZ34	**CINAL UNCTG**	Uncultured termite group 1 bacterium phylotype Rs-D17	Environmental sample
Q59VN7	**Q59VN7 CANAL**	Candida albicans (strain SC5314)	Eukaryota Fungi
B9W7J7	**B9W7J7 CANDC**	Candida dubliniensis (strain CD36)	Eukaryota Fungi
A5DWE6	**A5DWE6 LODEL**	Lodderomyces elongisporus (strain ATCC 11503)	Eukaryota Fungi
A6TNW2	**CINA ALKMQ**	Alkaliphilus metalliredigens (strain QYMF)	Firmicutes
A8MI49	**CINA ALKOO**	Alkaliphilus oremlandii (strain OhILAs)	Firmicutes
B7GJN4	**CINA ANOFW**	Anoxybacillus flavithermus (strain DSM 21510)	Firmicutes
A7Z4W4	**CINA BACA2**	Bacillus amyloliquefaciens (strain FZB42)	Firmicutes
B7HDQ5	**CINA BACC4**	Bacillus cereus (strain B4264)	Firmicutes
B7ITN2	**CINA BACC2**	Bacillus cereus (strain G9842)	Firmicutes
A7GRB0	**CINA BACCN**	Bacillus cereus subsp. cytotoxis (strain NVH 391-98)	Firmicutes
Q5WFW9	**CINA BACSK**	Bacillus clausii (strain KSM-K16)	Firmicutes
Q9KAA5	**CINA BACHD**	Bacillus halodurans (strain ATCC BAA-125)	Firmicutes
Q65JF3	**CINA BACLD**	Bacillus licheniformis (strain DSM 13)	Firmicutes
A8FDG2	**CINA BACP2**	Bacillus pumilus (strain SAFR-032)	Firmicutes
P46323	**CINA BACSU**	Bacillus subtilis (strain 168)	Firmicutes
Q6HF34	**CINA BACHK**	Bacillus thuringiensis subsp. konkukian (strain 97-27)	Firmicutes

A9VS24	CINA BACWK	Bacillus weihenstephanensis (strain KBAB4)	Firmicutes
B9MQY2	CINA ANATD	Caldicellulosiruptor bescii (strain ATCC BAA-1888)	Firmicutes
A4XLD7	CINA CALS8	Caldicellulosiruptor saccharolyticus (strain ATCC 43494)	Firmicutes
Q97D94	CINA CLOAB	Clostridium acetobutylicum (strain ATCC 824)	Firmicutes
A6M320	CINA CLOB8	Clostridium beijerinckii (strain ATCC 51743)	Firmicutes
B1KSY5	CINA CLOBM	Clostridium botulinum (strain Loch Maree)	Firmicutes
A7G9W4	CINA CLOBL	Clostridium botulinum (strain Langeland)	Firmicutes
B8I7D0	CINA CLOCE	Clostridium cellulolyticum (strain ATCC 35319)	Firmicutes
Q18BS8	CINA CLOD6	Clostridium difficile (strain 630)	Firmicutes
B9E6E0	CINA CLOK1	Clostridium kluyveri (strain NBRC 12016)	Firmicutes
A0PXD7	CINA CLONN	Clostridium novyi	Firmicutes
Q0SWN5	CINA CLOPS	Clostridium perfringens (strain SM101)	Firmicutes
Q898T5	CINA CLOTE	Clostridium tetani (strain Massachusetts)	Firmicutes
A3DEA5	CINA CLOTH	Clostridium thermocellum (strain ATCC 27405)	Firmicutes
B1I313	CINA DESAP	Desulforudis audaxviator (strain MP104C)	Firmicutes
A4J5U0	CINA DESRM	Desulfotomaculum reducens (strain MI-1)	Firmicutes
Q82Z98	CINA ENTFA	Enterococcus faecalis (strain ATCC 700802)	Firmicutes
B1YMB8	CINA EXIS2	Exiguobacterium sibiricum (strain DSM 17290)	Firmicutes
B0S3U9	CINA FINM2	Finegoldia magna (strain ATCC 29328)	Firmicutes
Q5L0F7	CINA GEOKA	Geobacillus kaustophilus (strain HTA426)	Firmicutes
C5D9G1	CINA GEOSW	Geobacillus sp. (strain WCH70)	Firmicutes
8CXC3	CINA HALOH	Halothermothrix orenii (strain H 168)	Firmicutes
Q03R28	CINA LACBA	Lactobacillus brevis (strain ATCC 367)	Firmicutes
B3WCM0	CINA LACCB	Lactobacillus casei (strain BL23)	Firmicutes
B2GB14	CINA LACF3	Lactobacillus fermentum (strain NBRC 3956)	Firmicutes
Q88UZ3	CINA LACPL	Lactobacillus plantarum (strain ATCC BAA-793)	Firmicutes
A5VIW6	CINA LACRD	Lactobacillus reuteri (strain DSM 20016)	Firmicutes
B1N0C1	CINA LEUCK	Leuconostoc citreum (strain KM20)	Firmicutes
Q92BV8	CINA LISIN	Listeria innocua serovar 6a (strain CLIP 11262)	Firmicutes
Q71ZS3	CINA LISMF	Listeria monocytogenes serotype 4b (strain F2365)	Firmicutes
A0AIJ9	CINA LISW6	Listeria welshimeri serovar 6b (strain ATCC 35897)	Firmicutes
B1HR35	CINA LYSSC	Lysinibacillus sphaericus (strain C3-41)	Firmicutes
Q2RKI6	CINA MOOTA	Moorella thermoacetica (strain ATCC 39073)	Firmicutes
B2A3C4	CINA NATTJ	Natranaerobius thermophilus (strain ATCC BAA-1301)	Firmicutes
Q8EQR8	CINA OCEIH	Oceanobacillus iheyensis (strain DSM 14371)	Firmicutes
Q03EQ4	CINA PEDPA	Pediococcus pentosaceus (strain ATCC 25745)	Firmicutes
CA5D2R7	CINA PELTS	Pelotomaculum thermopropionicum (strain DSM 13744)	Firmicutes
Q5HGE7	Q5HGE7 STAAC	Staphylococcus aureus (strain COL)	Firmicutes
Q8E2R9	CINA STRA3	Streptococcus agalactiae serotype III (strain NEM316)	Firmicutes
C0MGB7	CINA STRS7	Streptococcus equi subsp. zooepidemicus (strain H70)	Firmicutes
A8AZT4	CINA STRGC	Streptococcus gordonii (strain Challis)	Firmicutes
Q8DRX2	CINA STRMU	Streptococcus mutans serotype c (strain ATCC 700610)	Firmicutes
C1C9K9	CINA STRP7	Streptococcus pneumoniae (strain 70585)	Firmicutes
B5XJ05	CINA STRPZ	Streptococcus pyogenes serotype M49 (strain NZ131)	Firmicutes
A4VYN4	CINA STRS2	Streptococcus suis (strain 98HAH33)	Firmicutes
B9WXF1	B9WXF1 STRSU	Streptococcus suis 89/1591	Firmicutes
Q5M6H3	CINA STRT2	Streptococcus thermophilus (strain ATCC BAA-250)	Firmicutes
B9DW66	CINA STRU0	Streptococcus uberis (strain ATCC BAA-854)	Firmicutes
Q67NW5	CINA SYMTH	Symbiobacterium thermophilum (strain T)	Firmicutes
Q0AXI8	CINAL SYNS3	Syntrophomonas wolfei subsp. wolfei (strain Goettingen)	Firmicutes

Q0IDD6	**CINAL SYNWW**	Syntrophomonas wolfei subsp. wolfei (strain Goettingen)	Firmicutes
B0K9N3	**CINA THEP3**	Thermoanaerobacter pseudethanolicus (strain ATCC 33223)	Firmicutes
Q8RA54	**CINA THETN**	Thermoanaerobacter tengcongensis (strain DSM 15242)	Firmicutes
Q8RHR9	**CINAL FUSNN**	Fusobacterium nucleatum subsp. nucleatum (strain ATCC 25586)	Fusobacteria
D1AL40	**D1AL40 SEBTE**	Sebaldella termitidis (strain ATCC 33386)	Fusobacteria
C1A7F4	**CINAL GEMAT**	Gemmatimonas aurantiaca (strain T-27)	Gemmatimonadetes
E6QAI2	**E6QAI2 9ZZZZ**	mine drainage metagenome	Metagenome
D2R7K4	**D2R7K4 PIRSD**	Pirellula staleyi (strain ATCC 27377)	Planctomycetes
B7J7A7	**B7J7A7 ACIF2**	Acidithiobacillus ferrooxidans (strain ATCC 23270)	Proteobacteria
B5EQ46	**B5EQ46 ACIF5**	Acidithiobacillus ferrooxidans (strain ATCC 53993)	Proteobacteria
A9CJ26	**CINA AGRT5**	Agrobacterium tumefaciens (strain C58)	Proteobacteria
D3RTT7	**D3RTT7 ALLVD**	Allochromatium vinosum (strain ATCC 17899)	Proteobacteria
B8J6V6	**CINAL ANAD2**	Anaeromyxobacter dehalogenans (strain 2CP-1)	Proteobacteria
A7HGS8	**CINAL ANADF**	Anaeromyxobacter sp. (strain Fw109-5)	Proteobacteria
A5ES77	**A5ES77 BRASB**	Bradyrhizobium sp. (strain BTAi1)	Proteobacteria
F4QYT4	**F4QYT4 BREDI**	Brevundimonas diminuta ATCC 11568	Proteobacteria
Q57D17	**Q57D17 BRUAB**	Brucella abortus biovar 1 (strain 9-941)	Proteobacteria
B1YSY9	**B1YSY9 BURA4**	Burkholderia ambifaria (strain MC40-6)	Proteobacteria
E8YFC1	**E8YFC1 9BURK**	Burkholderia sp. CCGE1001	Proteobacteria
Q47XX0	**CINAL COLP3**	Colwellia psychrerythraea (strain 34H)	Proteobacteria
C0QI20	**CINAL DESAH**	Desulfobacterium autotrophicum (strain ATCC 43914)	Proteobacteria
A8ZZX4	**CINAL DESOH**	Desulfococcus oleovorans (strain DSM 6200)	Proteobacteria
Q6AIZ4	**CINAL DESPS**	Desulfotalea psychrophila (strain LSv54)	Proteobacteria
B8J0U1	**CINAL DESDA**	Desulfovibrio desulfuricans (strain ATCC 27774)	Proteobacteria
D2BUH5	**D2BUH5 DICD5**	Dickeya dadantii (strain Ech586)	Proteobacteria
P51967	**YGAD ENTAG**	Enterobacter agglomerans (Erwinia herbicola)	Proteobacteria
K4YRF4	**K4YRF4 9ENTR**	Enterobacter sp. SST3	Proteobacteria
E5B2F5	**E5B2F5 ERWAM**	Erwinia amylovora ATCC BAA-2158	Proteobacteria
YFAY	**YFAY ECOLI**	Escherichia coli (strain K12)	Proteobacteria
P0A6G3	**YDEJ ECOLI**	Escherichia coli (strain K12)	Proteobacteria
YDEJ	**YGAD ECOLI**	Escherichia coli (strain K12)	Proteobacteria
B1LLK3	**CINAL ECOSM**	Escherichia coli (strain SMS-3-5)	Proteobacteria
I1B4U7	**I1B4U7 ECOLX**	Escherichia coli AI27	Proteobacteria
B5ED03	**CINAL GEOBB**	Geobacter bemidjiensis (strain Bem)	Proteobacteria
Q39Z82	**CINAL GEOMG**	Geobacter metallireducens (strain GS-15)	Proteobacteria
B9M363	**CINAL GEOSF**	Geobacter sp. (strain FRC-32)	Proteobacteria
C6E6X4	**CINAL GEOSM**	Geobacter sp. (strain M21)	Proteobacteria
Q74GV1	**CINAL GEOSL**	Geobacter sulfurreducens (strain ATCC 51573)	Proteobacteria
A5GDB4	**CINAL GEOUR**	Geobacter uraniireducens (strain Rf4)	Proteobacteria
O25606	**O25606 HELPY**	Helicobacter pylori (strain ATCC 700392)	Proteobacteria
C7BZK4	**C7BZK4 HELPB**	Helicobacter pylori (strain B38)	Proteobacteria
Q1CSR9	**Q1CSR9 HELPH**	Helicobacter pylori (strain HPAG1)	Proteobacteria
E1Q2T3	**E1Q2T3 HELPP**	Helicobacter pylori (strain PeCan4)	Proteobacteria
E1Q060	**E1Q060 HELPM**	Helicobacter pylori (strain SJM180)	Proteobacteria
D6XQJ5	**D6XQJ5 HELPV**	Helicobacter pylori (strain v225d)	Proteobacteria
B9XV77	**B9XV77 HELPX**	Helicobacter pylori 98-10	Proteobacteria
F9U1V6	**F9U1V6 MARPU**	Marichromatium purpuratum 984	Proteobacteria
A6EV18	**A6EV18 9ALTE**	Marinobacter algicola DG893	Proteobacteria
Q1DCC8	**CINAL MYXXD**	Myxococcus xanthus (strain DK 1622)	Proteobacteria
F9EYU4	**F9EYU4 9NEIS**	Neisseria macacae ATCC 33926	Proteobacteria

D5BZJ2	**D5BZJ2 NITHN**	Nitrosococcus halophilus (strain Nc4)	Proteobacteria
G9ALV0	**G9ALV0 PANAN**	Pantoea ananatis LMG 5342	Proteobacteria
Q3A1W5	**CINAL PELCD**	Pelobacter carbinolicus (strain DSM 2380)	Proteobacteria
A1ALN8	**CINAL PELPD**	Pelobacter propionicus strain DSM 2379	Proteobacteria
Q6LPD0	**Q6LPD0 PHOPR**	Photobacterium profundum (Photobacterium sp. (strain SS9)	Proteobacteria
C7BHV9	**C7BHV9 PHOAA**	Photorhabdus asymbiotica subsp. asymbiotica (strain ATCC 43949)	Proteobacteria
K1GRG5	**K1GRG5 PROMI**	Proteus mirabilis WGLW4	Proteobacteria
D4C4M2	**D4C4M2 PRORE**	Providencia rettgeri DSM 1131	Proteobacteria
Q15QS0	**Q15QS0 PSEA6**	Pseudoalteromonas atlantica (strain T6c)	Proteobacteria
I4K772	**I4K772 PSEFL**	Pseudomonas fluorescens SS101	Proteobacteria
P72227	**YGAD PSEPU**	Pseudomonas putida	Proteobacteria
Q48F93	**Q48F93 PSE14**	Pseudomonas syringae pv. phaseolicola (strain 1448A)	Proteobacteria
H2IVB2	**H2IVB2 RAHAC**	Rahnella aquatilis (strain ATCC 33071)	Proteobacteria
K5DXI3	**K5DXI3 RHILU**	Rhizobium lupini HPC(L)	Proteobacteria
D0ZPN9	**D0ZPN9 SALT1**	Salmonella typhimurium (strain 14028s)	Proteobacteria
D0ZUV6	**D0ZUV6 SALT1**	Salmonella typhimurium (strain 14028s)	Proteobacteria
Q8ZPI7	**Q8ZPI7 SALTY**	Salmonella typhimurium (strain LT2)	Proteobacteria
G0B7Z5	**G0B7Z5 SERSA**	Serratia plymuthica (strain AS9)	Proteobacteria
A1S257	**CINAL SHEAM**	Shewanella amazonensis	Proteobacteria
A6WTS6	**CINAL SHEB8**	Shewanella baltica (strain OS185)	Proteobacteria
Q12SN4	**CINAL SHEDO**	Shewanella denitrificans (strain OS217)	Proteobacteria
Q089L6	**CINAL SHEFN**	Shewanella frigidimarina (strain NCIMB 400)	Proteobacteria
B0TL92	**CINAL SHEHH**	Shewanella halifaxensis (strain HAW-EB4)	Proteobacteria
A3Q9C2	**CINAL SHELP**	Shewanella loihica (strain ATCC BAA-1088)	Proteobacteria
Q8EK32	**CINAL SHEON**	Shewanella oneidensis (strain MR-1)	Proteobacteria
A8GZ14	**CINAL SHEPA**	Shewanella pealeana (strain ATCC 700345)	Proteobacteria
B8CNH3	**CINAL SHEPW**	Shewanella piezotolerans (strain WP3)	Proteobacteria
A4YBU5	**CINAL SHEPC**	Shewanella putrefaciens	Proteobacteria
A8G1B1	**CINAL SHESH**	Shewanella sediminis (strain HAW-EB3)	Proteobacteria
B1KNC9	**CINAL SHEWM**	Shewanella woodyi (strain ATCC 51908)	Proteobacteria
Q31X65	**Q31X65 SHIBS**	Shigella boydii serotype 4 (strain Sb227)	Proteobacteria
I6FM95	**I6FM95 SHIDY**	Shigella dysenteriae 225-75	Proteobacteria
F6DY83	**F6DY83 SINMK**	Sinorhizobium meliloti (strain AK83)	Proteobacteria
A9FML0	**CINAL SORC5**	Sorangium cellulosum (strain So ce56)	Proteobacteria
A0LLA6	**CINAL SYNFM**	Syntrophobacter fumaroxidans (strain DSM 10017)	Proteobacteria
Q2LPL3	**CINAL SYNAS**	Syntrophus aciditrophicus (strain SB)	Proteobacteria
I3Y732	**I3Y732 THIV6**	Thiocystis violascens (strain ATCC 17096)	Proteobacteria
G2E2V4	**G2E2V4 9GAMM**	Thiorhodococcus drewsii AZ1	Proteobacteria
J1N2F8	**J1N2F8 VIBCL**	Vibrio cholerae HE-25	Proteobacteria
A9ZXU7	**A9ZXU7 YERPE**	Yersinia pestis biovar Orientalis str. F1991016	Proteobacteria
Q5NNF6	**Q5NNF6 ZYMMO**	Zymomonas mobilis subsp. mobilis (strain ATCC 31821)	Proteobacteria
C8WFR3	**C8WFR3 ZYMMN**	Zymomonas mobilis subsp. mobilis (strain NCIB 11163)	Proteobacteria
B0SB28	**CINAL LEPBA**	Leptospira biflexa serovar Patoc (strain Patoc 1)	Spirochaetes
Q04TG1	**CINAL LEPBJ**	Leptospira borgpetersenii serovar Hardjo-bovis (strain JB197)	Spirochaetes
Q8F5J2	**CINAL LEPIN**	Leptospira interrogans serogr Icterohaemorrhagiae (strain 56601)	Spirochaetes
P47361	**Y115 MYCGE**	Mycoplasma genitalium (strain ATCC 33530)	Tenericutes
Q9EXC9	**Y254 MYCPN**	Mycoplasma pneumoniae (strain ATCC 29342)	Tenericutes
A7HNE6	**CINAL FERNB**	Fervidobacterium nodosum (strain ATCC 35602)	Thermotogae
C5CFW1	**CINAL KOSOT**	Kosmotoga olearia (strain TBF 19.5.1)	Thermotogae
A9BHX8	**CINAL PETMO**	Petrotoga mobilis (strain DSM 10674)	Thermotogae

A6LL44	**CINAL THEM4**	Thermosipho melanesiensis (strain BI429)	Thermotogae
A8F4W4	**CINAL THELT**	Thermotoga lettingae (strain ATCC BAA-301)	Thermotogae
Q9S5X1	**CINAL THEMA**	Thermotoga maritima (strain ATCC 43589)	Thermotogae
B9KAS5	**CINAL THENN**	Thermotoga neapolitana (strain ATCC 49049)	Thermotogae
B2UNF6	**CINAL AKKM8**	Akkermansia muciniphila (strain ATCC BAA-835)	Verrucomicrobia
